# Effects of MDMA-assisted therapy for PTSD on self-experience

**DOI:** 10.1371/journal.pone.0295926

**Published:** 2024-01-10

**Authors:** Bessel A. van der Kolk, Julie B. Wang, Rachel Yehuda, Leah Bedrosian, Allison R. Coker, Charlotte Harrison, Michael Mithoefer, Berra Yazar-Klosinki, Amy Emerson, Rick Doblin

**Affiliations:** 1 Trauma Research Foundation, Brookline, MA, United States of America; 2 MAPS Public Benefit Corporation (MAPS PBC), San Jose, CA, United States of America; 3 James J. Peters Veterans Affairs Medical Center, Bronx, NY, United States of America; 4 Center for Psychedelic Psychotherapy and Trauma Research, Icahn School of Medicine at Mount Sinai, New York, NY, United States of America; 5 University of California, San Francisco, San Francisco, CA, United States of America; 6 Multidisciplinary Association for Psychedelic Studies (MAPS), San Jose, CA, United States of America; 7 Department of Psychiatry and Behavioral Sciences, Medical University of South Carolina, Charleston, SC, United States of America; Brown University, UNITED STATES

## Abstract

**Introduction:**

There is a resurgence of interest in the therapeutic potential of psychedelic substances such as 3,4-methylenedioxymethamphetamine (MDMA). Primary findings from our randomized, double-blind, placebo-controlled, multi-site Phase 3 clinical trial of participants with severe PTSD (NCT03537014) showed that MDMA-assisted therapy induced significant attenuation in the Clinician-Administered PTSD Scale for DSM-5 compared to Therapy with placebo. Deficits in emotional coping skills and altered self-capacities constitute major obstacles to successful completion of available treatments. The current analysis evaluated the differential effects of MDMA-assisted therapy and Therapy with placebo on 3 transdiagnostic outcome measures and explored the contribution of changes in self-experience to improvement in PTSD scores.

**Methods:**

Participants were randomized to receive manualized therapy with either MDMA or placebo during 3 experimental sessions in combination with 3 preparation and 9 integration therapy visits. Symptoms were measured at baseline and 2 months after the last experimental session using the 20-item Toronto Alexithymia Scale (TAS-20), the 26-item Self Compassion Scale (SCS), and the 63-item Inventory of Altered Self-Capacities (IASC).

**Results:**

90 participants were randomized and dosed (MDMA-assisted therapy, n = 46; Therapy with placebo, n = 44); 84.4% (76/90) had histories of developmental trauma, and 87.8% (79/90) had suffered multiple traumas. MDMA-assisted therapy facilitated statistically significant greater improvement on the TAS-20, the SCS, and most IASC factors of interpersonal conflicts; idealization disillusionment; abandonment concerns; identity impairment; self-awareness; susceptibility to influence; affect dysregulation; affect instability; affect skill deficit; tension reduction activities; the only exception was identity diffusion.

**Conclusion:**

Compared with Therapy with placebo, MDMA-assisted therapy had significant positive effects on transdiagnostic mental processes of self-experience which are often associated with poor treatment outcome. This provides a possible window into understanding the psychological capacities facilitated by psychedelic agents that may result in significant improvements in PTSD symptomatology.

## Introduction

There has been a resurgence of interest in the therapeutic potential of psychedelic substances such as tryptamines (e.g., psilocybin), ketamine, and phenethylamines (e.g., 3,4-methylenedioxymethamphetamine (MDMA)) [1, 2]. Based on its positive performance with significant and sustained reductions in PTSD symptoms and acceptable safety profiles, the FDA has designated MDMA-assisted therapy as a breakthrough therapy for PTSD. A pooled analysis of six Phase 2 trials showed that 54% of patients assigned to the MDMA-assisted therapy group no longer met criteria for PTSD after 2 experimental sessions [3]. Results of the first Phase 3 multisite study of MDMA-assisted therapy were published which confirmed the safety and efficacy of MDMA-assisted therapy in individuals with severe PTSD [4]. Compared to Therapy with placebo, MDMA-assisted therapy was found to induce significant attenuation in PTSD symptom severity scores (*p* < 0.0001, *d* =  0.91), suggesting a greater therapeutic effect of MDMA-assisted therapy over Therapy with placebo. The protocol for MDMA-assisted therapy consists of a 3-month long treatment with 3 dosing sessions, as well as 3 preparation and 9 integration therapy visits. All study participants received an equal, substantial dose of manualized therapy in addition to receiving either the MDMA or placebo. This provided us with an opportunity to explore the differential effects of therapy within MDMA-assisted therapy to gain a deeper understanding of the psychological change processes induced by this therapy.

Trauma-focused psychotherapy is considered a first line treatment for PTSD [5, 6]. However, the overall success rate with psychotherapeutic treatments for PTSD has been relatively disappointing. At least one-quarter of patients drop out of trauma-focused psychotherapy, and up to one-half are left with significant lingering symptoms [7–9]. Even patients who are considered responders often remain challenged by difficulties in emotion regulation, impulse control and interpersonal functioning [10–12], all of which seem to continue relatively independent from PTSD symptomatology [13, 14].

Many trauma survivors, particularly those with histories of child abuse (developmental trauma) have been shown to experience significant defects in a variety of transdiagnostic mental processes, including loss of a sense of safety, trust and self-worth, being unable to notice internal states (alexithymia), lack of a coherent sense of self, inability to modulate or tolerate distress, difficulties negotiating interpersonal conflicts and negative self-appraisals, such as shame, self-blame and low self-compassion [15, 16]. All of these have been shown to correlate with poor treatment outcome [17, 18].

Multiple studies have shown that reduced self-capacities interfere with successful completion of psychotherapy for PTSD [19, 20]. Difficulty with emotion regulation interfere with being able to disengage from trauma-related stimuli, which increases the probability of drop out due to an inability to manage distress arising during treatment [21]. Alexithymia, deficits in being able to identify and describe emotions, is associated with posttraumatic pathology [22–24], and with a lack of habituation to emotionally distressing stimuli [25]. Persons with high alexithymia scores have been shown to display low autonomic activity in response to any task performance, regardless of the level of emotional demand, including processing traumatic material [26].

Finally, self-compassion is a core component of overall mental health and well-being [27], often lacking in trauma survivors with PTSD who frequently experience self-loathing and self-blame [28, 29]. Low self-compassion scores are associated with anxiety, depression, narcissism, self-criticism, and with poor treatment responses [30].

MDMA’s effects on emotion regulation have been studied in healthy volunteers [31, 32]. These studies, have demonstrated a positive effect of MDMA on self-regulatory capacities and self-compassion. Higher levels of emotion-regulation and self-compassion have been shown to improve treatment results for a variety of psychological interventions [33, 34], which invites an exploration of the potential for MDMA in the treatment of PTSD. MDMA may differentially alter emotion recognition, depending on the emotional valence of the stimuli [35].

In the present study, we report results of three transdiagnostic outcome measures from a MDMA-assisted therapy Phase 3 trial that was designed to test treatment effects on PTSD symptoms and associated functional impairment. Specifically, we compared treatment effects on (1) alexithymia, (2) self-compassion, and (3) an inventory of altered self-capacities. Collectively, these measures characterized participants’ self-experience levels which is known to impact treatment outcomes. The primary aim of this analysis was to examine treatment effects on self-experience measures and whether improvements occurred independently of PTSD symptoms improvements. Further, we examined whether baseline self-experience levels were associated with change in PTSD symptoms and whether there were differences between treatment groups.

## Methods

### Study design

This paper assesses exploratory data from a randomized, double-blind, placebo-controlled study comparing safety and efficacy of MDMA-assisted therapy to Therapy with placebo in participants with severe PTSD [36]. Details such as recruitment and locations of the 15 sites are described in the primary outcome paper [4]. All participants, site staff, independent raters, and the sponsor were blind to participants group assignments until after database lock. All participants provided written informed consent at eligibility screening after ethics approval from local Institutional Review Boards.

### Participants

All participants met DSM-5 criteria for current PTSD with a symptom duration of six months or greater and a Clinician-Administered PTSD Scale for DSM-5 (CAPS-5) total severity score of 35 or greater at baseline. Exclusion criteria included primary psychotic, bipolar I, dissociative identity, personality disorders, current alcohol and substance use disorders, and any medical condition for which an acute, transient increase in blood pressure or heart rate would pose a medical concern. Full eligibility criteria are described in the study protocol (https://clinicaltrials.gov/study/NCT03537014).

### Intervention

Randomization was managed via an interactive web randomization system—ITClinical IWRS, version 11.0.1 (ITClinical, LDA)—based on a centralized randomization schedule developed by an independent third-party vendor to maintain blinding. All participants underwent three 90-minute preparation therapy sessions with a co-therapist dyad to establish therapeutic alliance and prepare for experimental sessions. The treatment period consisted of three 8-hour experimental sessions of either MDMA-assisted therapy or Therapy with placebo, with sessions spaced approximately four weeks apart [4].

In each experimental session, participants were given a split-dose of MDMA or placebo, with an initial dose followed by a half-dose 1.5 to 2.5 hours later. In the first experimental session the dose was 80 mg + 40 mg MDMA HCl, and in second and third experimental sessions, the dose was escalated to 120 mg + 60 mg MDMA HCl. Manualized therapy was conducted in accordance with MAPS MDMA-assisted therapy treatment manual (https://maps.org/2014/01/27/a-manual-for-mdma-assisted-therapy-in-the-treatment-of-ptsd/). Following each experimental session, participants underwent three 90-minute integration sessions, scheduled one week apart, to provide them with the opportunity to process their experiences.

### Demographic and baseline variables

Age, gender, ethnicity, race, and education were compared between treatment groups. Other variables relevant to the transdiagnostic outcomes explored here, but not reported in this publication, included employment status, detailed trauma history, pre-study treatment, and baseline outcomes measures for the Adverse Childhood Experience Questionnaire (ACE) [37], Beck Depression Inventory II (BDI-II) [38], CAPS-5 total severity score [36], and lifetime suicidality assessment from the Columbia Suicide Severity Rating Scale (C-SSRS) [39].

### Self-experience measures

The Inventory of Altered Self Capacities (IASC), is a standardized self-reported measure of an individual’s psychological functioning and has been frequently utilized in treatment outcome studies of PTSD [15, 40, 41]. It consists of 63 items to measure difficulties with relationships, identity, and emotion regulation, rated on a 5-point Likert scale ranging from 1 (“Never”) to 5 (“Very Often”). The IASC consists of 11 factors and sub-factors: Items for sub-factors are summed to calculate factor raw scores that range from 9 to 45 [42].

The Toronto Alexithymia Scale (TAS-20), is a well-validated 20-item measure of self-reported difficulties with recognizing and verbalizing emotions [43]. Responses are reported on a 5-point Likert scale ranging from 1 (“Strongly disagree”) to 5 (“Strongly agree”). The scale is comprised of three subscales: Difficulty Describing Feelings, Difficulty Identifying Feelings, and Externally-Oriented Thinking. Total scores diagnostically indicate no alexithymia (≥50), border-line alexithymia (51–60), and alexithymia (≥61) [22].

The Self-Compassion Scale (SCS) is a valid and theoretically coherent self-reported measure of self-compassion [44]. The SCS consists of 26 items to measure how respondents perceive their own failures, suffering, or inadequacies with kindness and compassion as a part of the common human experience. Respondents indicate how they often feel for each item on a 5-point Likert scale ranging from 1 (“Almost never”) to 5 (“Almost always”). The SCS consists of six subscales: Self-Kindness, Self-Judgement, Common Humanity, Isolation, Mindfulness, and Over-Identified, in which the sum of each subscale scores serves as the total score. A total score of 1–2.4 indicates “low,” 2.5–3.4 “moderate,” and 3.5–5.0 “high” SCS [45].

Independent raters conducted the PTSD primary outcome assessment, CAPS-5, prior to the first experimental session and at the primary endpoint Visit 19, approximately eight weeks after the final experimental session (18 weeks post-baseline). TAS-20, SCS, and IASC were self-reported at baseline, during the final preparation session (Visit 4), and again approximately 18 weeks later at study termination (Visit 20).

### Statistical methods

Descriptive analyses were performed on demographic, baseline, and outcome variables. Group means (SD) were compared using *t*-tests or ANOVA/ ANCOVA and proportions were compared using chi-square tests. Shapiro Wilk W tests were performed to determine normality and non-parametric tests were performed on samples with non-normal distributions. Pearson’s correlations were conducted to examine linear relationships across variables. General Linear Models (GLM) were performed which allows to build a linear relationship between the response and predictors even when the underlying relationship is non-linear and therefore can be used to analyze non-normal data [46].

In the primary analysis, separate two-way ANCOVA models, adjusting for their respective baseline scores and CAPS-5 dissociative subtype (Yes = 1 and No = 0), compared treatment group differences in change scores for TAS-20, SCS, each IASC factor, and CAPS-5 (MDMA-assisted therapy vs. Therapy with placebo). Additional analyses were performed to also adjust for CAPS-5 change scores to assess potential independent effects of each self-experience measure on PTSD.

Separate analyses examined within-subjects differences at baseline and follow-up scores for MDMA-assisted therapy and Therapy with placebo groups. Sub-set analyses evaluated change scores stratified by baseline cutoff scores; specifically: (i) TAS-20 baseline measure of having no alexithymia (≤50) and alexithymia (>51) [47]; (ii) SCS baseline measure of low (1–2.4) and moderate (2.5–3.4) or high (3.5–5.0) self-compassion [48]; (iii) and for each IASC factor baseline scores for each factor above and below the sample median. For IASC factors, the sample median (vs. the mean) was used to account for any non-normal sample distributions and since the IASC lacks a validated composite score to define a clinical cutoff. Models tested interaction terms between treatment group (MDMA-assisted therapy vs. Therapy with placebo) and baseline categories (low vs. high TAS-20, SCS, or IASC factor) and where appropriate the main effects. All models adjusted for baseline scores and CAPS-5 dissociative subtype (Yes = 1 and No = 0). Tukey’s HSD test corrected for multiple comparisons and tables reported Least Square Means (LSMEANS) which adjusted for unequal sample sizes across group comparisons. All analyses were performed using SAS Version 9.4 (SAS Institute, Cary, North Carolina).

## Results

### Sample characteristics

The study sample consisted of 90 participants who were randomized and completed at least one experimental dosing session (MDMA-assisted therapy = 46, Therapy with placebo = 44; **S1 Fig**). Follow-up data for TAS-20, SCS, and IASC were missing for eight participants due to early study termination (discontinued due to COVID-19 = 3; declined further treatment = 4; restarted pre-study treatment = 1). All available data were used in the analysis (n = 82). In the present analysis, participants were majority women (53 of 82; 64.6%), White (65 of 81; 80.3%), non-Hispanic or Latino (76 of 82; 92.7%), college graduates (57 of 82; 69.5%) and, among 82 participants, the mean (SD) age was 41.42 (12.22) years. Sixty-nine of 82 participants (84.2%) had histories of developmental trauma (e.g., childhood physical/sexual abuse), and 74 of 82 participants (90.2%) had suffered multiple traumas. Only 4 out of 90 subjects in this study had an Adverse Childhood Experience (ACE) score of 0. Among the 8 participants with missing outcome data, no remarkable differences were observed with respect to treatment group (4 were in MDMA-assisted therapy and 4 Therapy with placebo) or sociodemographic characteristics although baseline CAPS-5 total severity scores were higher (n = 8; 46.75, SD = 6.63) compared to the modified Intent-to-treat (*mITT*) analysis set (n = 90; 44.1, SD = 6.04). There were no statistically significant group differences between MDMA-assisted therapy and Therapy with placebo groups across demographic and baseline variables. Detailed sample characteristics of the *mITT* analysis set of n = 90 participants have been described in the primary outcome paper [4].

Baseline means (SD) for the overall study sample were as follows: TAS-20, 54.33 (12.24); SCS, 2.28 (0.77); IASC interpersonal conflicts, 2.55 (0.96); idealization disillusionment, 2.13 (1.04); abandonment concerns, 2.49 (1.08); self-awareness, 3.09 (1.16); identity diffusion, 2.15 (1.13); susceptibility to influence, 1.97 (0.89); affect instability, 2.69 (1.17); affect skill deficit, 2.97 (1.20); and tension reduction activities, 1.87 (0.64). For each outcome measure, *t*-tests were performed and there were no baseline differences between treatment groups.

### Treatment effects on self-experience measures

**Fig 1:**
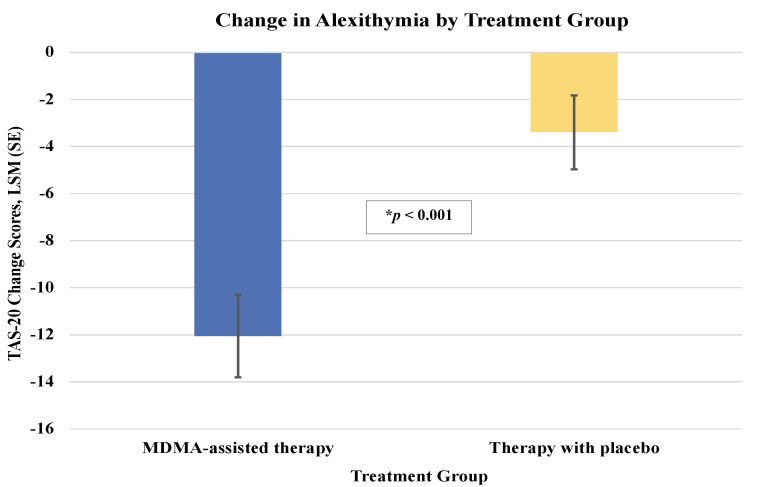
Alexithymia change scores in MDMA-assisted therapy. Least square means (SE) change in Toronto Alexithymia Scale (TAS-20) scores from baseline to follow up by treatment group: MDMA-assisted therapy = -12.06 (1.75) vs Therapy with placebo = -3.39 (1.57), *p* < .0001.

**Fig 2:**
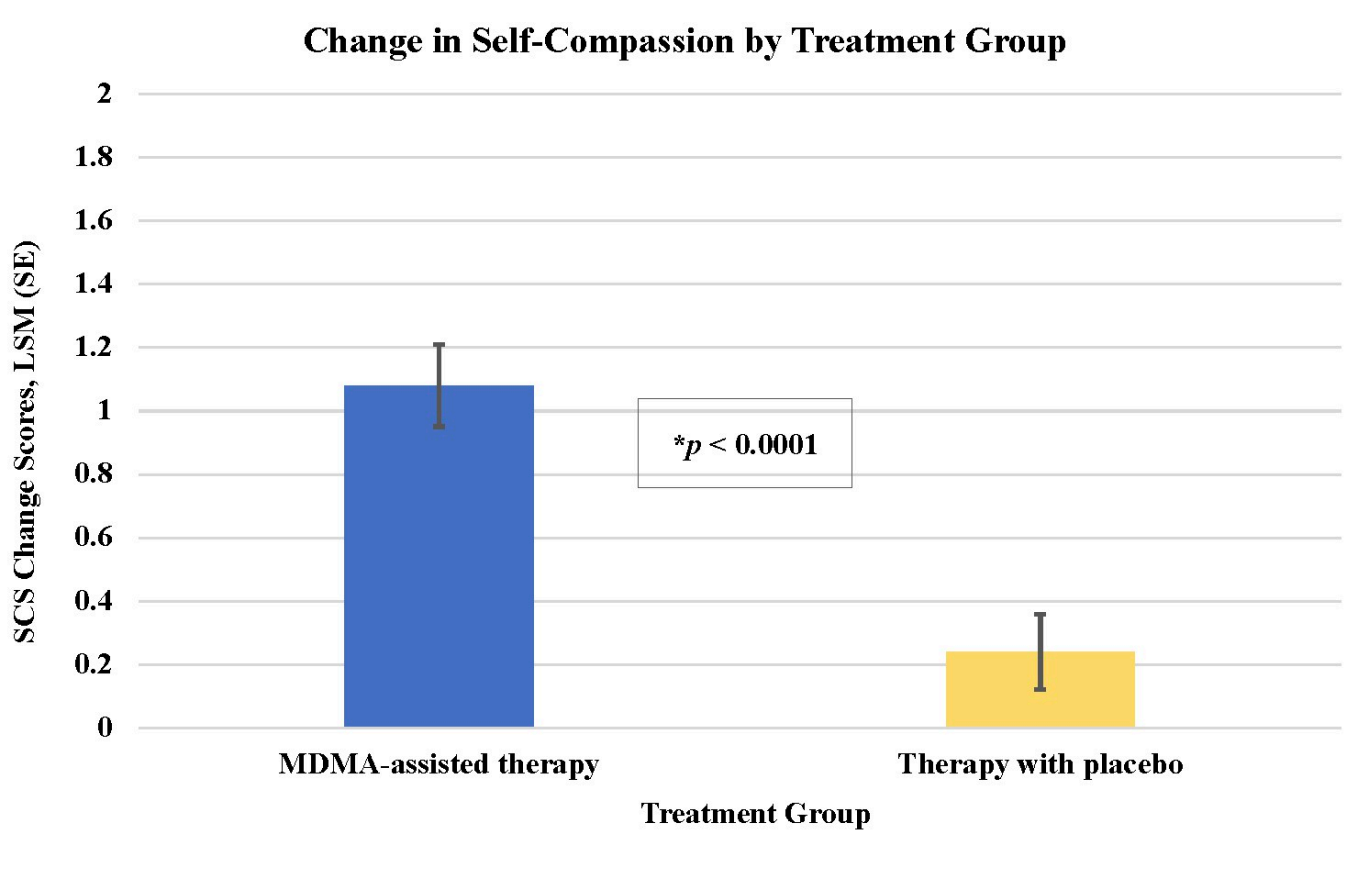
**Self-compassion change scores in MDMA-assisted therapy.** Least square means (SE) change in Self-compassion Scale (SCS) from baseline to follow-up by treatment group: MDMA-assisted therapy = 1.08 (0.13) vs. Therapy with placebo = 0.24 (0.12), *p* < .0001.

**Fig 3:**
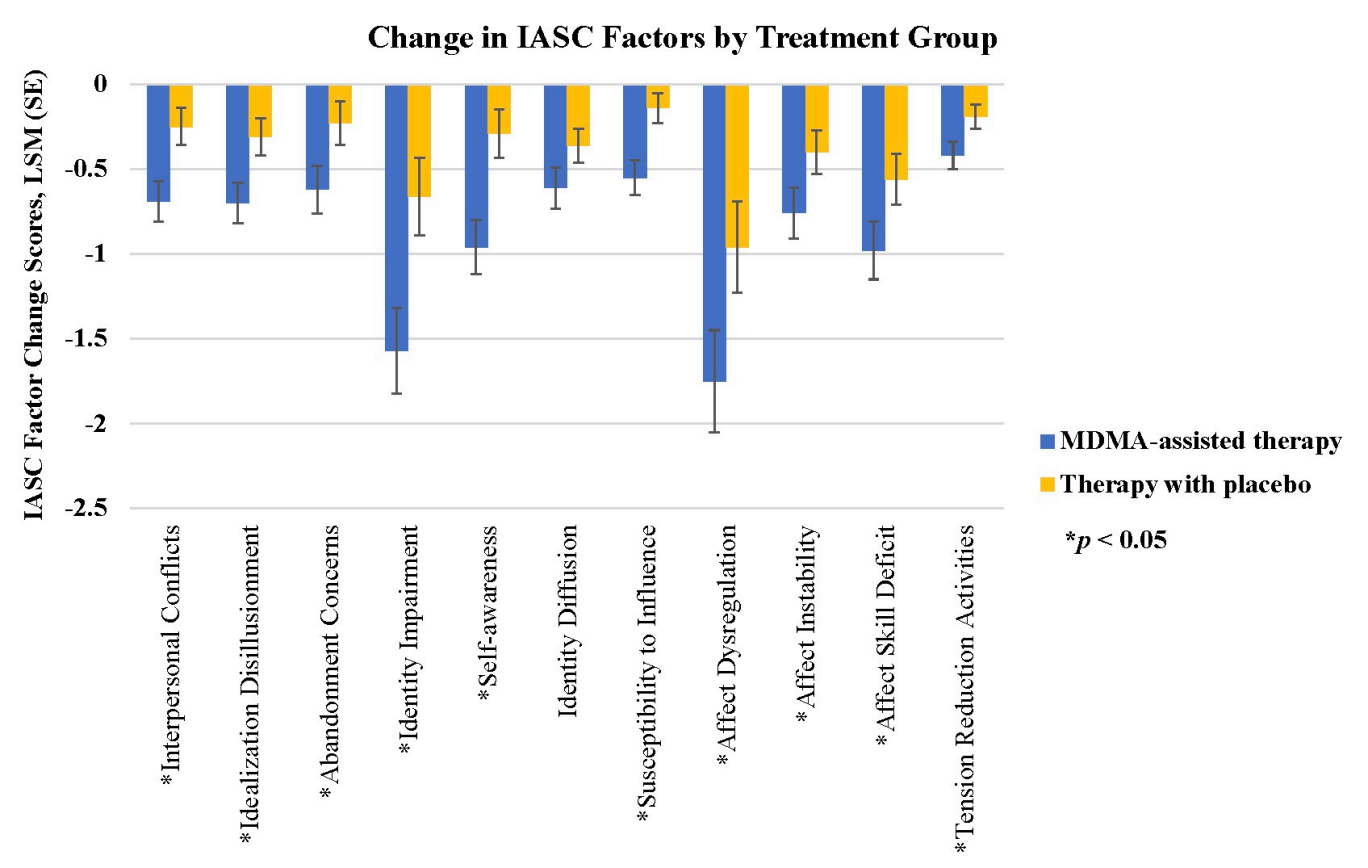
Inventory of Altered Self-capacities (IASC) change scores. Least square means (SE) change from baseline to follow-up by MDMA-assisted therapy vs. Therapy with placebo: (i) “interpersonal conflicts” -0.69 (0.12) vs. -0.25 (0.11), *p* = .0027; (ii) “idealization disillusionment” -0.70 (0.12) vs. -0.31 (0.11), *p* = .0095; (iii) “abandonment concerns” -0.62 (0.14) vs. -0.23 (0.13), *p* = .0293; (iv) “identity impairment” -1.57 (0.25) vs. -0.66 (0.23), *p* = .0036; (v) “self-awareness” -0.96 (0.16) vs. -0.29 (0.14), *p* = .0010; (vi) “identity diffusion” -0.61 (0.12) vs. -0.36 (0.10), *p* = .0757; (vii) “susceptibility to influence” -0.55 (0.10) vs. -0.14 (0.09), *p* = .0012; (viii) “affect dysregulation” -1.75 (0.30) vs. -0.96 (0.27), *p* = .0349; (ix) “affect instability” -0.76 (0.15) vs. -0.40 (0.13), *p* = .0454; (x) “affect skill deficit” -0.98 (0.17) vs. -0.56 (0.15), *p* = .0424; (xi) “tension reduction activities” -0.42 (0.08) vs. -0.19 (0.07), *p* = .0206.

The MDMA-assisted therapy group, compared to the Therapy with placebo group, had statistically significant greater improvements for all self-experience measures except for IASC factor identity diffusion **(Tables 1 and 2 and Figs 1–3)**. MDMA-assisted therapy, vs. Therapy with placebo, had greater improvements on alexithymia **(Fig 1)**, self-compassion (**Fig 2**), and most IASC factors **(Fig 3)**. These results suggest that MDMA had a strong effect on these measures of emotion regulation and self-experience, even after adjusting for potential covariates and correcting for multiple comparisons. Only results for SCS change scores were stable and statistically significant after also adjusting for CAPS-5 change score (unadjusted for CAPS-5 change, *p* < .0001; adjusted for CAPS-5 change, *p* = 0.0076) **(Table 1)**.

**Table 1:** Change in self-experience scores by treatment group–interaction terms and main effects.

	Interaction Term^1^			Main Treatment Effects^2^			Main Treatment Effects adjusted for CAPS-5 change scores^3^^,^^5^		
	*F*-statistic	η^2^ ^4^	*p*-value^6^	*F*-statistic	η^2^ ^4^	*p*-value^6^	*F*-statistic	η^2^ ^4^	*p-*value^6^
TAS-20^5^	*F* (2, 76) = 1.17	0.02	0.32	*F* (1, 76) = 15.21	0.13	0.0002*	*F* (1, 74) = 2.48	0.02	0.12
SCS^5^	*F* (2, 76) = 2.85	0.05	0.06	*F* (1, 76) = 22.75	0.18	< .0001*	*F* (1,74) = 7.53	0.04	0.0076*
IASC^5^									
Interpersonal Conflicts	*F* (2, 76) = 0.11	0.002	0.89	*F* (1, 76) = 9.59	0.06	0.003*	*F* (1, 74) = 1.15	0.0063	0.29
Idealization Disillusionment	*F* (2, 76) = 0.77	0.011	0.47	*F* (1, 76) = 6.87	0.047	0.01*	*F* (1, 74) = 1.34	0.009	0.25
Abandonment Concerns	*F* (2, 76) = 0.01	0.0002	0.99	*F* (1, 76) = 4.81	0.039	0.03*	*F* (1, 74) = 0.01	0.0001	0.92
Identity Impairment	*F* (2, 76) = 1.99	0.0267	0.14	*F* (1, 76) = 9.34	0.0624	0.003*	*F* (1, 74) = 0.51	0.0025	0.48
Self-awareness	*F* (2, 76) = 1.00	0.015	0.37	*F* (1,76) = 8.33	0.063	0.005*	*F* (1, 74) = 0.28	0.0015	0.60
Identity Diffusion	*F* (2, 76) = 0.00	0.0001	1.00	*F* (1, 76) = 3.10	0.0181	0.08	*F* (1, 74) = 0.03	0.0002	0.86
Susceptibility to Influence	*F* (2, 76) = 1.04	0.0099	0.36	*F* (1, 76) = 12.12	0.0579	0.0008*	*F* (1, 74) = 1.42	0.005	0.24
Affect Dysregulation	*F* (2, 76) = 0.52	0.0086	0.60	*F* (1, 76) = 5.09	0.0422	0.03*	*F* (1, 74) = 0.06	0.0004	0.80
Affect Instability	*F* (2, 76) = 0.79	0.0118	0.46	*F* (1, 76) = 4.68	0.035	0.03*	*F* (1, 74) = 0.04	0.0002	0.84
Affect Skill Deficit	*F* (2, 76) = 3.81	0.06	0.03*	--	--	--	--	--	--
Tension Reduction Activities	*F* (2, 76) = 0.22	0.0026	0.80	*F* (1, 76) = 5.81	0.0338	0.02*	*F* (1, 74) = 0.28	0.0014	0.60

^1^ Interaction term: treatment x baseline self-experience subgroup levels (e.g., TAS-20, SCS, IASC factor)

^2^ Main effects: reported when the interaction term was not statistically significant at alpha level .05; models adjusted for baseline self-experience levels (TAS-20, SCS, or IASC factor) and baseline CAPS-5 dissociative subtype

^3^ Model adjusted for baseline self-experience subgroup levels (TAS-20, SCS, or IASC factor), baseline CAPS-5 dissociative subtype, and CAPS-5 change scores

^4^ Variance: η^2^ = semi-partial eta squared

^5^ Abbreviations: CAPS-5 = Clinician-administered PTSD Scale for DSM-5; TAS-20 = Toronto Alexithymia Scale; SCS = Self-compassion Scale; IASC = Inventory of Altered Self-capacities

^6^ *statistical significance at *p* < .05

**Table 2:** Alexithymia, self-compassion, and altered self-capacities scores by treatment group and baseline self-experience levels.

	Therapy with placebo					MDMA-assisted therapy					Between-group difference in change scores
	N	Baseline	N	Follow-up	Change^4^^,^^5^^,^^7^	N	Baseline	N	Follow-up	Change^4^^,^^5^^,^^7^	*p*-value^6^^,^^7^
Baseline TAS-20^1^^,^^2^, mean (SD)	44	55.73 (10.64)	40	51.20 (11.47)	-3.39 (1.57)*	46	53.00 (13.75)	42	41.62 (11.71)	-12.06 (1.75)*	< .0001*
No alexithymia	18	45.56 (3.70)	17	43.41 (9.49)	-6.24 (2.76)*	19	39.84 (7.91)	17	35.12 (8.30)	-11.72 (3.22)*	0.35
Alexithymia	26	62.77 (7.71)	23	56.96 (9.29)	-1.51 (2.26)	27	62.26 (8.28)	25	46.04 (11.74)	-12.85 (2.43)*	< .0006*
Baseline SCS^1^^,^^3^, mean (SD)	44	2.18 (0.68)	40	2.51 (0.86)	0.24 (0.12)^	46	2.38 (0.83)	42	3.50 (0.81)	1.08 (0.13)*	< .0001*
Moderate or High	14	2.98 (0.45)	13	3.22 (0.84)	0.38 (0.23)	19	3.22 (0.54)	18	3.76 (0.62)	0.76 (0.23)*	.42
Low	30	1.80 (0.38)	27	2.16 (0.64)	0.23 (0.16)	27	1.79 (0.35)	24	3.30 (0.88)	1.36 (0.20)*	< .0001*
Baseline IASC^1^, mean (SD)											
Interpersonal Conflicts	44	2.57 (0.93)	40	2.27 (0.84)	-0.25 (0.11)*	46	2.53 (0.99)	42	1.81 (0.58)	-0.69 (0.12)*	.0027*
≤ median 2.33	24	1.90 (0.33)	22	2.00 (0.64)	-0.29 (0.16)	23	1.74 (0.29)	21	1.55 (0.49)	-0.68 (0.17)*	.21
> median	20	3.36 (0.78)	18	2.61 (0.95)	-0.20 (0.19)	23	3.33 (0.77)	21	2.07 (0.55)	-0.72 (0.20)*	.08
Idealization-Disillusionment	44	2.25 (1.08)	40	1.86 (0.93)	-0.31 (0.11)*	46	2.02 (0.99)	42	1.39 (0.59)	-0.70 (0.12)*	.0095*
≤ median 1.84	19	1.29 (0.22)	17	1.35 (0.57)	-0.48 (0.19)*	26	1.29 (0.23)	24	1.12 (0.36)	-0.69 (0.18)*	.71
> median	25	2.98 (0.89)	23	2.23 (0.97)	-0.20 (0.17)	20	2.97 (0.76)	18	1.75 (0.65)	-0.75 (0.21)*	.05
Abandonment Concerns	44	2.57 (1.01)	40	2.22 (0.99)	-0.23 (0.13)	46	2.41 (1.14)	42	1.70 (0.80)	-0.62 (0.14)*	.0293*
≤ median 2.28	20	1.62 (0.34)	18	1.78 (0.87)	-0.21 (0.24)	25	1.50 (0.37)	24	1.32 (0.54)	-0.59 (0.24)*	.39
> median	24	3.37 (0.62)	22	2.59 (0.94)	-0.26 (0.22)	21	3.50 (0.70)	18	2.20 (0.84)	-0.64 (0.27)*	.43
Identity Impairment	44	5.50 (2.21)	40	4.70 (2.08)	-0.66 (0.23)*	46	5.00 (1.97)	42	3.72 (1.71)	-1.57 (0.25)*	.0036*
≤ median 4.83	20	3.52 (0.79)	19	3.54 (1.00)	-1.26 (0.41)	25	3.55 (0.91)	24	2.70 (0.75)	-2.11 (0.38)*	.1594
> median	24	7.15 (1.55)	21	5.59 (1.62)	-0.10 (0.38)	21	6.73 (1.42)	18	4.53 (1.81)	-1.09 (0.41)*	.1419
Self-Awareness	44	3.22 (1.19)	40	2.85 (1.03)	-0.29 (0.14)^	46	2.97 (1.12)	42	2.08 (0.98)	-0.96 (0.16)*	.0010*
≤ median 3.00	23	2.23 (0.60)	21	2.48 (0.91)	-0.01 (0.26)	23	2.01 (0.57)	20	1.65 (0.60)	-0.66 (0.29)*	.09
> median	21	4.30 (0.56)	19	3.25 (1.01)	-0.62 (0.28)*	23	3.93 (0.55)	22	2.46 (1.10)	-1.14 (0.25)*	.28
Identity Diffusion	44	2.28 (1.21)	40	1.78 (0.85)	-0.36 (0.10)*	46	2.03 (1.05)	42	1.40 (0.69)	-0.61 (0.12)*	.0757
≤ median 1.75	22	1.30 (0.31)	21	1.38 (0.55)	-0.36 (0.18)*	25	1.20 (0.27)	23	1.09 (0.21)	-0.60 (0.18)*	.58
> median	22	3.26 (0.93)	19	2.21 (0.92)	-0.36 (0.20)	21	3.02 (0.70)	19	1.79 (0.86)	-0.63 (0.20)*	.61
Susceptibility to Influence	44	2.04 (0.92)	40	1.76 (0.73)	-0.14 (0.09)	46	1.90 (0.86)	42	1.28 (0.41)	-0.55 (0.10)*	.0012*
≤ median 1.78	23	1.37 (0.26)	21	1.49 (0.56)	-0.29 (0.14)^	23	1.22 (0.22)	20	1.17 (0.42)	-0.58 (0.16)*	.33
> median	21	2.76 (0.83)	19	2.05 (0.80)	0.02 (0.15)	23	2.58 (0.69)	22	1.38 (0.37)	-0.56 (0.15)*	.0099*
Affect Dysregulation	44	5.85 (2.14)	40	4.70 (2.08)	-0.96 (0.27)*	46	5.48 (2.28)	42	3.72 (1.71)	-1.75 (0.30)*	.0349*
≤ median 5.80	22	4.1 (1.04)	21	3.74 (1.69)	-1.30 (0.44)*	23	3.46 (0.88)	21	2.85 (1.16)	-1.92 (0.53)*	.6381
> median	22	7.61 (1.35)	19	5.76 (1.97)	-0.54 (0.51)	23	7.49 (1.16)	21	4.59 (1.74)	-1.63 (0.50)*	.1763
Affect Instability	44	2.81 (1.10)	40	2.29 (0.99)	-0.40 (0.13)*	46	2.57 (1.23)	42	1.84 (0.84)	-0.76 (0.15)*	.0454*
≤ median 2.50	22	1.92 (0.48)	21	1.82 (0.67)	-0.55 (0.22)*	26	1.62 (0.46)	24	1.52 (0.68)	-0.73 (0.25)*	.88
> median	22	3.70 (0.76)	19	2.80 (1.04)	-0.24 (0.24)	20	3.81 (0.65)	18	2.26 (0.87)	-0.84 (0.26)*	.12
Affect Skill Deficit	44	3.04 (1.24)	40	2.41 (1.24)	-0.56 (0.15)*	46	2.90 (1.17)	42	1.88 (0.92)	-0.98 (0.17)*	.0424*
≤ median 3.00	22	1.99 (0.69)	20	1.60 (0.60)	-0.41 (0.57)*	24	1.97 (0.67)	22	1.50 (0.65)	-0.43 (0.83)*	1.0
> median	22	4.09 (0.60)	20	3.22 (1.19)	-0.84 (1.45)	22	3.93 (0.59)	20	2.29 (1.00)	-1.60 (0.92)*	.02*
Tension Reduction Activities	44	1.88 (0.57)	40	1.66 (0.48)	-0.19 (0.07)*	46	1.85 (0.71)	42	1.40 (0.45)	-0.42 (0.08)*	.0206*
≤ median 1.78	20	1.43 (0.20)	18	1.54 (0.43)	-0.13 (0.12)	26	1.34 (0.26)	24	1.26 (0.41)	-0.37 (0.12)*	.30
> median	24	2.25 (0.50)	22	1.75 (0.52)	-0.24 (0.10)*	20	2.52 (0.53)	18	1.60 (0.44)	-0.47 (0.14)*	.36

^1^ Abbreviations: TAS-20 = Toronto Alexithymia Scale; SCS = Self-Compassion Scale; IASC = Inventory of Altered Self-Capacities; ASC = Altered Self-Capacities

^2^ TAS-20 cutoff scores: no alexithymia ≤50; borderline alexithymia (51–60); alexithymia (≥61) (Bagby et al. 1994)

^3^ SCS cutoff scores: low (1–2.4); moderate (2.5–3.4); high (3.5–5.0) (Neff 2003)

^4^ Change scores are Least Square Means (Standard Errors)

^5^ (*) = indicates a *p*-value of < .05 for within-subjects comparison of baseline vs. follow-up scores; (^) *p* = 0.05

^6^ (*) indicates a *p*-value of < .05 for between-group subjects’ comparison of Therapy with placebo change scores vs. MDMA-assisted therapy change scores; (^) *p* = 0.05

^7^ All models adjusted for baseline CAPS-5 Dissociative Subtype (Yes/ No), baseline self-experience score (TAS-20, SCS, or IASC factor score), and corrected for multiple comparisons using Tukey’s HSD

### Baseline self-experience measures & treatment effects on PTSD symptoms

**Fig 4:**
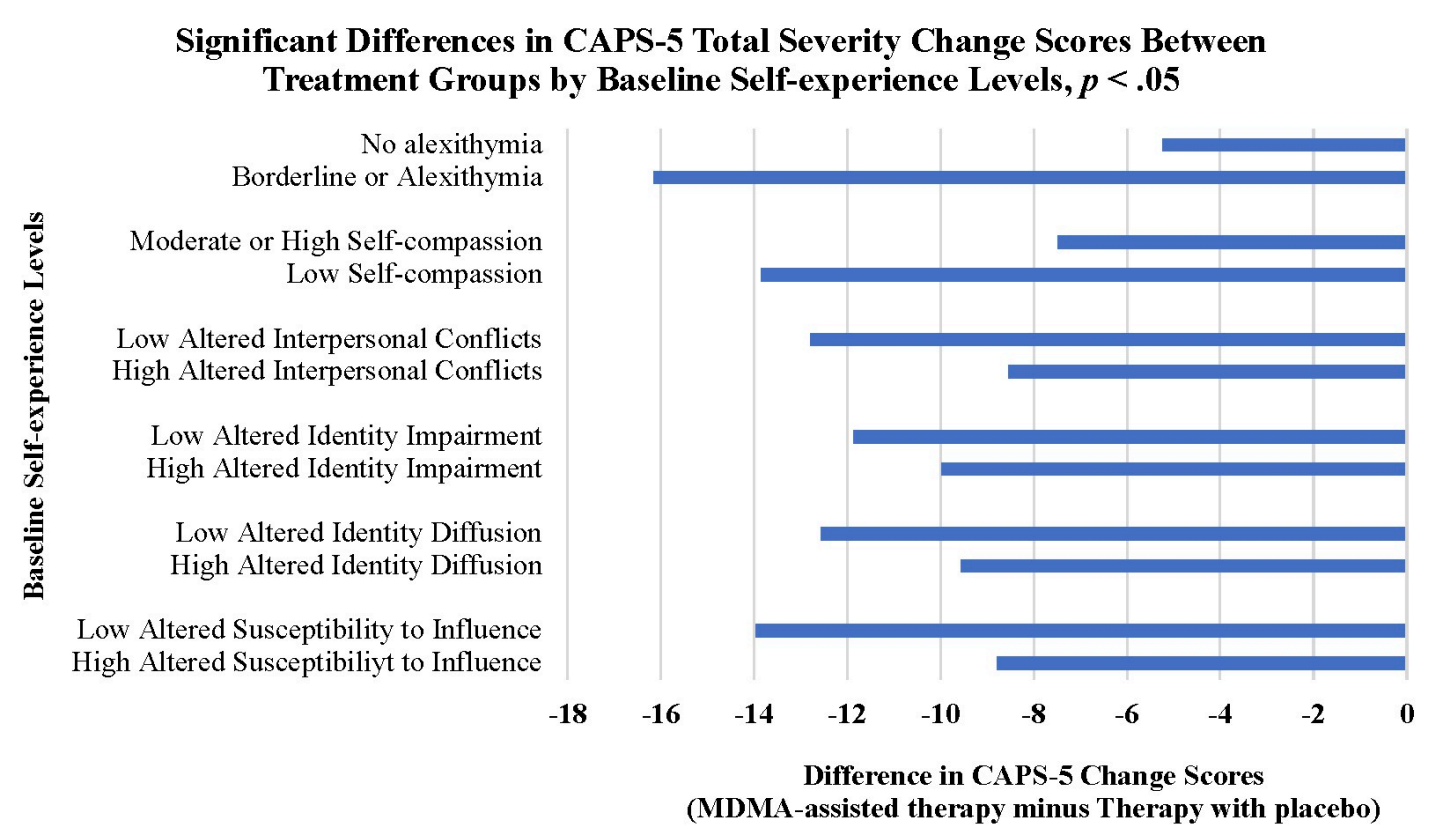
Significant differences in CAPS-5 total severity change scores between treatment groups by baseline self-experience levels. Interaction between treatment and baseline alexithymia subgroup levels was statistically significant to warrant further stratification (*p* = 0.04); and stratified results also presented for self-compassion and IASC scores. At baseline, being (i) worse-off, having borderline alexithymia/ alexithymia or low self-compassion or (ii) better-off, lower idealization disillusionment, identity impairment, identity diffusion, or susceptibility to influence were associated with statistically significant greater CAPS-5 changes scores in the MDMA-assisted therapy group compared to Therapy with placebo, at alpha *p* ≤ .05 (*). For all other IASC factors, there were no differences in CAPS-5 change scores by baseline subgroup levels.

Overall, MDMA-assisted therapy, compared to Therapy with placebo, had statistically significant greater improvement in all self-experience measures **(Tables 3 and 4)**. Results showed a significant interaction between treatment x baseline TAS-20 subgroup levels to warrant further examination of CAPS-5 change scores by baseline levels. There was a greater reduction in CAPS-5 scores in the MDMA-assisted therapy group for those who had begun the trial with greater baseline alexithymia (-16.16; 95% CI: -28.80, -7.52) **(Fig 4)**, and there were statistically significant differences between baseline subgroups in CAPS-5 change scores for (i) MDMA-assisted therapy/ low TAS-20 vs. Therapy with placebo/ high TAS-20 (*p* = 0.02) and (ii) MDMA-assisted therapy/ high TAS-20 vs. Therapy with placebo/ high TAS-20 (*p* < .0001) (all data not shown). **Table 4** reports CAPS-5 change scores for all self-experience outcomes by baseline subgroup levels.

**Table 3:** Change in CAPS-5 total severity change scores by treatment group–interaction terms and main effects.

	Interaction Term^1^			Main Treatment Effects^2^		
	*F*-statistic	η^2^ ^3^	*p*-value^5^	*F*-statistic	η^2^ ^3^	*p*-value^5^
TAS-20^4^	*F* (1, 76) = 4.61	0.0435	0.04*	--	--	--
SCS^4^	*F* (1, 76) = 1.34	0.0139	0.25	*F* (1, 76) = 14.77	0.1538	0.0003*
IASC^4^						
Interpersonal Conflicts	*F* (1, 76) = 0.34	0.0036	0.56	*F* (1, 76) = 17.30	0.1826	< 0.0001*
Idealization Disillusionment	*F* (1, 76) = 0.69	0.0070	0.41	*F* (1, 76) = 15.53	0.1583	0.0002*
Abandonment Concerns	*F* (1, 76) = 0.14	0.0015	0.71	*F* (1, 76) = 17.04	0.1803	< 0.0001*
Identity Impairment	*F* (1, 76) = 0.25	0.0025	0.62	*F* (1, 76) = 16.07	0.1618	0.0001*
Self-awareness	*F* (1, 76) = 0.02	0.0002	0.88	*F* (1, 76) = 17.75	0.1878	<0.0001*
Identity Diffusion	*F* (1, 76) = 0.36	0.0037	0.55	*F* (1, 76) = 16.70	0.1723	0.0001*
Susceptibility to Influence	*F* (1, 76) = 0.98	0.0102	0.32	*F* (1, 76) = 17.89	0.1861	< 0.0001*
Affect Dysregulation	*F* (1, 76) = 0.05	0.0005	0.83	*F* (1, 76) = 18.51	0.1875	< 0.0001*
Affect Instability	*F* (1, 76) = 0.07	0.0007	0.80	*F* (1, 76) = 17.51	0.1847	< 0.0001*
Affect Skill Deficit	*F* (1, 76) = 0.20	0.0021	0.66	*F* (1, 76) = 18.13	0.1872	< 0.0001*
Tension Reduction Activities	*F* (1, 76) = 0.09	0.0009	0.77	*F* (1, 76) = 16.37	0.1713	0.0001*

^1^ Interaction term: treatment x baseline self-experience subgroup levels (e.g., TAS-20, SCS, IASC factor)

^2^ Main effects: reported when the interaction term was not statistically significant at alpha level .05; models adjusted for baseline self-experience subgroup levels (TAS-20, SCS, or IASC factor) and baseline CAPS-5 dissociative subtype

^3^ Variance: η^2^ = semi-partial eta squared

^4^ Abbreviations: CAPS-5 = Clinician-administered PTSD Scale for DSM-5; TAS-20 = Toronto Alexithymia Scale; SCS = Self-compassion Scale; IASC = Inventory of Altered Self-capacities

^5^ (*) Statistical significance at p < .05

**Table 4:** CAPS-5 PTSD total severity scores by treatment group and baseline self-experience levels.

	Therapy with placebo			MDMA-assisted therapy					
	Baseline (n = 44)	Visit 20 (n = 40)	Change	Baseline (n = 46)	Visit 20 (n = 42)	Change	N	Between-group difference in change scores	95% CI
Overall Sample	44.23 (6.15)	30.48 (12.56)	-13.32 (1.95)*	43.98 (6.01)	19.55 (13.50)	-24.65 (2.18)*	82	-11.33	-16.59, 6.08
Baseline TAS-20, mean (SD)^1^^,^^2^^,^^8^									
No alexithymia	45.19 (2.64)	24.61 (2.79)	-20.59 (3.75)*	42.38 (2.60)	17.96 (2.73)	-24.41 (3.64)*	33	-5.24	-15.44, 4.97
Borderline or Alexithymia	44.18 (2.14)	34.98 (2.23)	-9.19 (3.09)	46.23 (2.16)	21.77 (2.25)	-24.46 (3.03)*	49	-16.16	-28.80, -7.52*
Baseline SCS, mean (SD)^1^^,^^3^^,^^8^									
Moderate or High	42.47 (2.97)	26.01 (3.07)	-16.46 (4.23)*	43.16 (2.56)	20.64 (2.62)	-22.52 (3.61)*	31	-7.48	-18.67, 3.70
Low	45.92 (2.02)	33.48 (2.13)	-12.43 (2.91)*	46.38 (2.21)	20.66 (2.35)	-25.72 (3.08)*	51	-13.85	-22.84, -4.86*
Baseline IASC, mean (SD)^1^									
Interpersonal Conflicts									
≤ median 2.33	45.00 (2.25)	31.73 (2.40)	-13.27 (3.25)*	43.43 (2.32)	18.09 (2.42)	-25.33 (3.29)*	42	-12.80	-22.38, -3.21*
> median	45.03 (2.44)	30.71 (2.51)	-14.32 (3.49)*	47.37 (2.35)	23.93 (2.47)	-23.44 (3.29)*	40	-9.72	-19.79, 0.35
Idealization-Disillusionment^8^									
≤ median 1.84	46.04 (2.52)	30.97 (2.72)	-15.07 (3.64)*	44.03 (2.15)	17.12 (2.25)	-26.91 (3.04)*	40	-12.80	-22.61, -2.98*
> median	44.33 (2.15)	31.51 (2.20)	-12.82 (3.07)*	47.40 (2.49)	26.43 (2.62)	20.96 (3.49)*	42	-8.54	-18.41, 1.34
Abandonment Concerns^8^									
≤ median 2.28	45.00 (2.47)	31.47 (2.66)	-13.54 (3.56)*	42.91 (2.23)	18.17 (2.28)	-24.72 (3.09)*	41	-12.29	-22.12, -2.46*
> median	44.87 (2.21)	30.94 (2.26)	-13.93 (3.15)*	48.04 (2.42)	24.45 (2.61)	-23.59 (3.47)*	41	-10.29	-20.34, -0.23*
Identity Impairment^8^									
≤ median 4.83	45.64 (2.43)	29.94 (2.49)	-15.70 (3.38)*	42.71 (2.17)	15.87 (2.21)	-26.84 (3.01)*	43	-11.87	-21.18, -2.56*
> median	44.49 (2.15)	32.43 (2.30)	-12.07 (3.15)*	48.61 (2.38)	27.88 (2.58)	-20.73 (3.39)*	39	-9.98	-20.41, 0.45
Self-Awareness									
≤ median 3.00	44.27 (2.35)	29.94 (2.39)	-14.33 (3.28)*	42.53 (2.36)	17.53 (2.52)	-24.99 (3.36)*	42	-11.05	-20.62, -1.48*
> median	45.45 (2.40)	32.44 (2.59)	-13.01 (3.53)*	47.61 (2.36)	23.53 (2.43)	-24.09 (3.28)*	40	-11.88	-22.21, -1.54*
Identity Diffusion^8^									
≤ median 1.75	47.00 (2.34)	32.06 (2.44)	-14.94 (3.31)*	43.73 (2.19)	17.19 (2.29)	-26.54 (3.10)*	43	-12.56	-21.92, -3.21*
> median	43.38 (2.29)	30.77 (2.40)	-12.61 (3.31)*	48.09 (2.44)	26.37 (2.55)	-21.71 (3.40)*	39	-9.57	-19.83, 0.69
Susceptibility to Influence^8^									
≤ median 1.78	44.76 (2.33)	31.25 (2.49)	-13.51 (3.34)*	43.76 (2.33)	17.13 (2.50)	-26.63 (3.34)*	40	-13.96	-23.68, -4.24*
> median	45.21 (2.39)	31.17 (2.45)	-14.05 (3.42)*	46.90 (2.37)	24.41 (2.41)	-22.50 (3.26)*	42	-8.78	-18.65, 1.09
Affect Dysregulation^8^									
≤ median 5.80	44.42 (2.34)	27.58 (2.45)	-16.83 (3.34)*	43.30 (2.34)	16.83 (2.44)	-26.47 (3.27)*	41	-10.81	-20.37, -1.24*
> median	45.07 (2.32)	34.38 (2.43)	-10.69 (3.34)	46.52 (2.31)	24.19 (2.42)	-22.33 (3.27)*	41	-12.07	-21.71, -2.44*
Affect Instability^8^									
≤ median 2.50	44.53 (2.41)	29.35 (2.52)	-15.18 (3.42)*	43.82 (2.27)	19.11 (2.35)	-24.71 (3.13)*	44	-10.66	-20.08, -1.25*
> median	45.01 (2.36)	32.67 (2.48)	-12.35 (3.42)*	46.45 (2.52)	22.50 (2.66)	-23.95 (3.60)*	38	-12.03	-22.28, -1.77*
Affect Skill Deficit									
≤ median 3.00	44.16 (2.32)	28.11 (2.37)	-16.06 (3.28)*	42.88 (2.28)	17.39 (2.37)	-25.50 (3.17)*	43	-10.18	-19.67, -0.70*
> median	45.40 (2.31)	34.24 (2.48)	-11.17 (3.37)	47.28 (2.34)	24.10 (2.46)	-23.17 (3.32)*	39	-12.70	-22.60, -2.79*
Tension Reduction Activities^8^									
≤ median 1.78	44.67 (2.48)	28.71 (2.67)	-15.96 (3.60)*	43.73 (2.22)	18.59 (2.30)	-25.14 (3.09)*	41	-10.20	-20.01, -0.39*
> median	44.95 (2.23)	32.79 (2.29)	-12.16 (3.18)*	46.77 (2.49)	23.41 (2.64)	-23.36 (3.54)*	41	-11.82	-21.73, -1.91*

^1^ Abbreviations: TAS-20 = Toronto Alexithymia Scale; SCS = Self-Compassion Scale; IASC = Inventory of Altered Self-Capacities; ASC = Altered Self-Capacities

^2^ TAS-20 cutoff scores: no alexithymia ≤50; borderline alexithymia (51–60); alexithymia (≥61) (Bagby et al. 1994)

^3^ SCS cutoff scores: low (1–2.4); moderate (2.5–3.4); high (3.5–5.0) (Neff 2003)

^4^ Change scores are Least Square Means (Standard Errors)

^5^ (*) = indicates a *p*-value of < .05 for within-subjects comparison of baseline vs. follow-up scores

^6^ (*) indicates a *p*-value of < .05 for between-group subjects’ comparison of Therapy with placebo change scores vs. MDMA-assisted therapy change scores

^7^ All models adjusted for baseline CAPS-5 Dissociative Subtype (Yes/ No), baseline self-experience score (TAS-20, SCS, or IASC score), change in TAS-20, SCS, or IASC scores, and corrected for multiple comparisons using Tukey’s HSD

^8^ Baseline levels predicted CAPS-5 change scores

## Discussion

In this study, the MDMA-assisted therapy group compared to Therapy with placebo had greater improvements in all self-experience measures, except IASC factor identity diffusion, and higher baseline alexithymia was associated with greater improvements in PTSD. Only improvements in self-compassion occurred independently of PTSD change scores. The evidence suggests alexithymia and most IASC factors likely mediated the effects of MDMA-assisted therapy treatment on PTSD symptoms (Table 1). Additional analysis showed baseline alexithymia moderated treatment effects on PTSD symptoms, which warranted examination of CAPS-5 changes scores stratified by baseline TAS-20 subgroup levels (Table 3); and those with higher alexithymia scores (those worse off) at baseline had greater PTSD symptoms improvement (Table 4). Results of this exploratory analysis show both the potential influence of baseline self-experience and MDMA-assisted therapy on self-experience to impact PTSD symptoms, which can be used to guide clinical practice. Further, results found MDMA-assisted therapy improved self-compassion independent of PTSD treatment which warrants further investigation into potential new applications.

In non-PTSD studies MDMA has been shown to promote a general sense of interpersonal “connectedness” [49] “openness” [50, 51], and to enhance positive appraisal of favorable memories, while reducing negative evaluations of painful memories [51]. It also has been shown to enhance extinction of fearful memories, modulate memory reconsolidation (possibly through an oxytocin-dependent mechanism), and to promote social behavior [52]. Moreover, MDMA inhibits habitual fear responses to emotional threats [53]. These qualities are thought to facilitate being able to put the emotional sequelae of painful past experiences into a realistic perspective.

In this study, we examined the effects of MDMA on a group of individuals with major clinical deficits in domains that are associated with treatment resistance. Our findings suggest that the therapeutic benefits of MDMA may be most pertinent for persons with clinically significant impairment in emotion regulation and self-capacities.

The vast majority (84%) of traumatized individuals in this study reported having suffered chronic early childhood trauma, i.e. physical or sexual abuse by their caregivers. Only 4 out of 90 subjects in this study had an Adverse Childhood Experience (ACE) score of 0. Histories of child maltreatment are associated with poorer responses to psychotherapy in individuals diagnosed with PTSD [50, 54]. Abuse at the hands of one’s early caregivers has been shown to put individuals at risk for deficits in emotional coping skills /altered self-capacities, major obstacles to successful completion of currently available evidence-based treatments [50, 55].

Being able to emotionally process traumatic experiences is an important element of successful treatment [56, 57]. Identifying feelings, describing them and recognizing their triggers are thought to allow an individual to reflect on the situation and to respond appropriately to the context, rather than acting solely on their emotional arousal [58]. An inability to do so, as expressed in alexithymia, avoidance of distressing wishes, feelings or experiences, and trouble recalling distressing experiences, are associated with impaired affect regulation [23–25].

Alexithymia has frequently been observed in the context of invalidating or abusive early environments where children learn that communicating emotional experiences is inappropriate, ineffective, or potentially dangerous [59, 60]. Unable to escape physically from chronic abuse, alexithymic individuals are thought to have learned to disengage from both their external reality as well as their internal experiences [61].

Even though the MDMA-assisted therapy experimental sessions often occurred in relative silence as participants focus largely on their inner experience, MDMA-assisted therapy, but not Therapy with placebo, was associated with a significant improvement in emotional self-awareness and loss of alexithymia. This suggests that MDMA-assisted therapy can facilitate accessing painful memories and experiences that under ordinary conditions are too overwhelming and terrifying to confront, even in the presence of trained therapists.

Problems with emotion regulation (ER) influence both the development and the maintenance of PTSD symptoms after exposure to potentially traumatizing experiences [8, 62, 63], and predict both functional impairment and symptom complexity [50]. Adaptive emotion regulation is essential for effective treatment of PTSD: trauma-focused treatments for PTSD require both activation and modification of fearful memories. This activation depends on two processes: physiological reactivity to trauma-related stimuli and being able to tolerate the subjective distress generated by these traumatic memories [64]. Being able to tolerate physiological arousal to trauma-related stimuli predicts improvement in exposure treatment, supporting a gradual diminution in the distress experienced in response to trauma recall (habituation) within- and between-sessions [65].

Emotion regulation deficits are major contributors to the development of a large variety of psychopathological conditions [66], including interference with being able to resolve the impact of traumatizing experience(s) [67–70]. Whereas healthy, flexible ER capacities are key factors underlying well-being, ER difficulties comprise a transdiagnostic risk factor for mental health problems in general, including the development and/or maintenance of symptoms of PTSD [71], by interfering with being able to disengage from trauma-related stimuli and inhibiting maladaptive emotion regulation strategies [72].

Self-compassion is another core component of overall mental health and well-being. Individuals suffering from traumatic stress often suffer from shame, self-blame and self-loathing [28, 29]. Appraisals of mental defeat and permanent change have a profound and debilitating effect on an individual’s identity and sense of self [73]. Low self compassion scores have consistently been associated with symptoms such as anxiety, depression, narcissism, self criticism and avoidance [27, 30, 65]. Self-compassion has been shown to boost the efficacy of cognitive reappraisals [74]: Being caring and kind to oneself, rather than critical, even under stress, can mitigate the negative effects of trauma exposure by increasing resilience and by decreasing avoidance-oriented coping [75, 76]. Notably, in this study, improvement in self-compassion occurred independently of improvements in PTSD symptoms, which confirms previous studies that have demonstrated a powerful effect of MDMA-assisted therapy on self-compassion per se [77].

The finding that participants in the placebo condition, who received a total of 36 hours of therapy during the course of the study, had significantly less improvement in the dimensions of alexithymia and self compassion is interesting and deserves further research. After all, an important focus in psychotherapy is to help individuals to become more self aware and self-accepting. The therapists in this study were experienced clinicians with previous trainings in various trauma focused therapies, including the MAPS method. Yet, participants in the MDMA condition, who, in the presence of these supportive and validating therapists, spent three experimental sessions with an internal focus on deep emotional encounters with the residues of their traumatic past, developed significantly more self compassion and self-awareness than those who only received Therapy with placebo.

Recently, questions have been raised about the optimal way to ensure safety and support for vulnerable people engaged in a psychedelic induced encounter with past trauma [78]. The role and specifics of psychotherapeutic assistance during the administration of MDMA are important research questions that should be approached with great caution since visiting devastating traumatic experiences of one’s past can be very distressing and require a delicate therapeutic approach focused on careful attention to set and setting; over fifty years of clinical experience suggest that set and setting are critical in achieving positive outcomes in psychedelic therapies [79].

### Summary

MDMA may be particularly effective for enhancing treatment efficacy by improving a range of problems with self-experience that are associated with treatment resistance. Assessment of self-capacities may be as relevant for treatment planning and outcome research as measuring PTSD severity, because, as this study suggests, therapy alone may not sufficiently compensate for the debilitating effects of deficient self-experience on being able to deal with traumatic material and thus, on treatment outcome.

### Limitations

This study had a strict protocol of psychological intervention supplemented with history taking, debriefing and integration sessions. Several study participants expressed a desire for further MDMA-assisted therapy sessions beyond the study protocol, particularly individuals with chronic interpersonal trauma who experienced considerable distress around issues of abandonment and separation at study termination. Many subjects in this study had experienced trauma at the hands of their early caregivers, which raised major issues during the therapy sessions around trust and security of attachment relationships. This study did not measure the effect of MDMA-assisted therapy on trust and intimacy in interpersonal relationships. Many study participants experienced considerable somatic distress while accessing traumatic material during the MDMA sessions, which was not assessed, nor investigated further, but potential shifts in somatic self-experience are hypothesized to be of considerable interest for future MDMA-assisted therapy research.

This study was a secondary analysis of exploratory outcome measures and did not control for age and nature of trauma exposure and may not reflect an epidemiologically representative PTSD population. Some of this disparity can be attributed to a lower percentage of non-White participants seeking treatment [80], which warrants systematic changes to reduce cultural barriers to increase engagement in clinical research [81]. A total of 8 participants were missing follow-up data for TAS-20, SCS, and IASC. As stated in the results section, all available data were used; no imputations were carried out. We used sample medians to set cutoff scores for baseline IASC factors since there were no referenced categories from the published literature; and this binary threshold could have missed to capture any statistically significant results. More studies are needed to examine the capacity of MDMA to ameliorate post-traumatic symptomatology in a variety of trauma populations, including whether MDMA-assisted therapy is capable of permanently altering a host of psychological processes associated with having been traumatized, including shame, self-blame, the capacity for emotional intimacy, executive functioning and affect regulation.

## Supporting information

S1 Checklist CONSORT checklist. The CONSORT Checklist confirms where in the publication CONSORT components can be found.
(DOC)

S1 Fig CONSORT diagram. The CONSORT flow for this analysis aligns with the primary publication for this study [4].
(PDF)

S1 Protocol MAPP1 study protocol. The MAPP1 Study Protocol summarizes the rationale and study design for the MAPP1 study.
(PDF)

S1 File MAPP1 statistical analysis plan. The MAPP1 Statistical Analysis Plan summarizes the statistical analyses used to evaluate the MAPP1 study findings.
(PDF)
