# Supplementary figures and images for: Effects of MDMA-assisted therapy for PTSD on self-experience
Source: PLoS One. 2024 Jan 10;19(1):e0295926. doi: 10.1371/journal.pone.0295926 (PMC10781106; doi:10.1371/journal.pone.0295926)

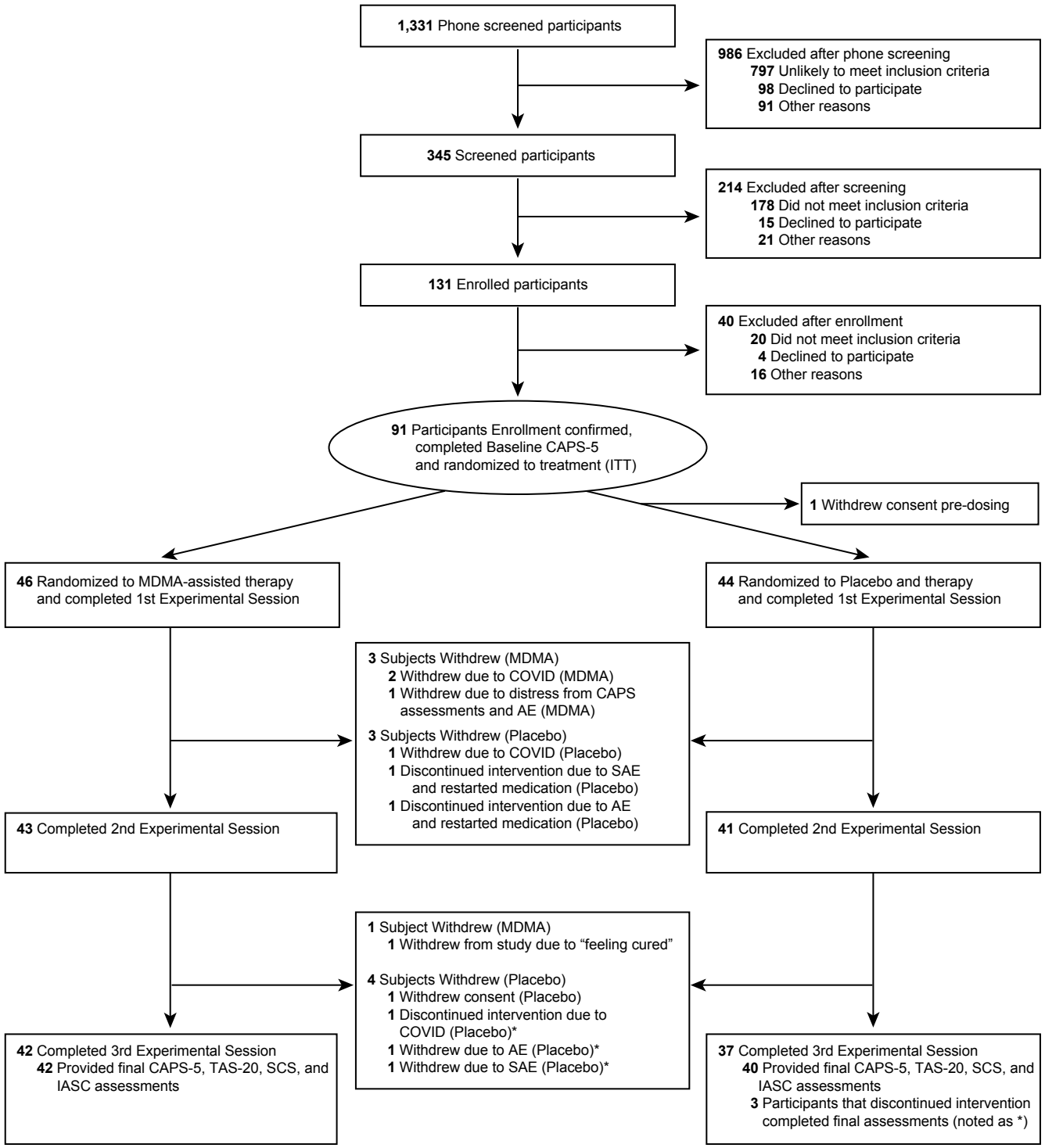

Supplement: S1 Fig — The CONSORT flow for this analysis aligns with the primary publication for this study [4]. (PDF) [file pone.0295926.s002.pdf]
