# Supplementary material for: Effects of MDMA-assisted therapy for PTSD on self-experience
Source: PLoS One. 2024 Jan 10;19(1):e0295926. doi: 10.1371/journal.pone.0295926 (PMC10781106; doi:10.1371/journal.pone.0295926)
Supplement: S1 Protocol — The MAPP1 Study Protocol summarizes the rationale and study design for the MAPP1 study. (PDF) [file pone.0295926.s003.pdf]

Protocol MAPP1
IND #063384

Original Protocol Version 1: 16 January 2017
Original Protocol Version 2: 07 June 2017
Amendment 1 Version 1: 26 February 2018
Amendment 2 Version 1: 12 September 2019
Amendment 3 Version 1: 13 November 2019
Amendment 4 Version 1: 22 May 2020

A Randomized, Double-Blind, Placebo-Controlled, Multi-Site Phase 3 Study of the Efficacy and Safety of Manualized MDMA-Assisted Psychotherapy for the Treatment of Severe Posttraumatic Stress Disorder

SPONSOR	Multidisciplinary Association for Psychedelic Studies (MAPS)<br>1115 Mission Street<br>Santa Cruz, CA 95060
SPONSOR REPRESENTATIVE	Rick Doblin, Ph.D.<br>Executive Director<br>MAPS
SPONSOR DELEGATE<br>&TRIAL ORGANIZER	MAPS Public Benefit Corporation<br>1115 Mission Street<br>Santa Cruz, CA 95060
SPONSOR DESIGNEE	Amy Emerson<br>Executive Director<br>MAPS Public Benefit Corporation
CLINICAL INVESTIGATOR	Multi-site Study
MEDICAL MONITOR	Michael C. Mithoefer, M.D.<br>Alia Lilienstein, M.D., M.P.H.
USE	In conjunction with relevant Food and Drug Administration (FDA), Health Canada (HC), and Israeli Ministry of Health (MOH) guidance

Public MAPP1 Protocol Synopsis

Full protocol begins on Page 11.

Rationale

The Multidisciplinary Association for Psychedelic Studies (MAPS) is a non-profit research and educational organization working as a clinical trial sponsor to obtain approval for the prescription use of 3,4-methylenedioxymethamphetamine (MDMA) as an adjunct to psychotherapy in patients with posttraumatic stress disorder (PTSD). PTSD is a serious debilitating disorder that negatively impacts a person's daily life, and can result in diminished cognitive and psychosocial functioning, fractured relationships, inability to maintain employment, substance abuse, high-cost healthcare utilization, increased depression, and suicide risk. People who suffer from PTSD relive their traumatic experience(s) through nightmares and flashbacks, have difficulty sleeping, and feel detached or estranged. Symptoms can be severe and long lasting.

MDMA is a monoamine releaser and re-uptake inhibitor with indirect effects on neurohormone release. The combined neurobiological effects reduce defenses and fear of emotional injury, enhance communication and introspection, and increase empathy and compassion. MDMA may enhance fear extinction learning in humans. The subjective effects of MDMA create a productive psychological state that enhances the therapeutic process. Data from an international series of Phase 2 pilot studies of MDMA-assisted psychotherapy conducted by the sponsor provide preliminary evidence that chronic PTSD, independent of cause, is treatable with two to three sessions of MDMA-assisted psychotherapy and associated non-drug preparatory and integrative psychotherapy. This Phase 3 study is intended to confirm the efficacy and safety of manualized MDMA-assisted psychotherapy as a treatment for PTSD.

Study Design

This multi-site, randomized, double-blind study assesses the efficacy and safety of MDMA-assisted psychotherapy versus psychotherapy with placebo control in participants diagnosed with at least severe PTSD. A flexible dose of MDMA hydrochloride salt (referred to as MDMA throughout) or placebo, followed by a supplemental half-dose unless contraindicated, is administered during the Treatment Period with manualized psychotherapy in three blinded monthly Experimental Sessions. This ~12-week Treatment Period is preceded by three Preparatory Sessions. During the Treatment Period, each Experimental Session is followed by three Integrative Sessions of non-drug psychotherapy. Experimental Sessions are followed by an overnight stay; a sub-study (Appendix A) will assess feasibility of Experimental Sessions without an overnight stay. The Primary Outcome measure, the change in Clinician Administered PTSD Scale (CAPS-5), is assessed by a blinded centralized Independent Rater (IR) pool multiple times throughout the study.

Any sub-studies other than what is outlined in Appendix A will be detailed in separate documents.

For each participant, the study will consist of:

- **Screening Period**: phone screen, informed consent, eligibility assessment, and enrollment of eligible participants
- **Preparatory Period with Enrollment Confirmation**: medication tapering, Preparatory Sessions and Baseline assessments leading to Enrollment Confirmation
- **Treatment Period**: three monthly Experimental Sessions and associated Integrative

Sessions over ~12 weeks

- **Follow-up Period and Study Termination**: 4 weeks with no study visits, followed by Study Termination visit
- **Invitation to participate in Long-term Follow-up (LTFU) extension study**: 12 months after last Experimental Session.

After study unblinding which occurs following database lock, participants in the placebo group, regardless of their CAPS-5 scores, will be offered the opportunity to enroll at no cost in an open-label safety extension study to receive treatment with the same method of MDMA-assisted psychotherapy as in the initial study. Data will be included in the sponsor's overall safety database.

Study Design Overview

**Screening Period**<br>From Consent to Enrollment ~4 weeks (+/-2 weeks)			
**Study Visit**		**Visit Duration/ Visit Timing**	**Brief Description of Events**
Screening	Screening	Multiple visits over 7 to 28 days/ After phone screen/	At initial visit, obtain Informed Consent and assess all screening measures (including PCL-5, Lifetime C-SSRS), medical history, and pre-study medications. Contact outside providers and order medical records, physical exam, labs (including pregnancy and drug tests), ECG, and 1-minute rhythm strip. Once all results and records are obtained, review along with notes from all screening visits and measures. If eligible, send results of LEC-5 & SCID-5-SPQ to IR. Screening may take place over 7 to 28 days at multiple visits.
	Independent Rater Screening	1 hour/<br>2 to 9 days after initial eligibility established during Screening	After PCL-5 and initial eligibility are reviewed, an IR will conduct the Since Last Visit C-SSRS, SCID-5-PD, DDIS, and MINI via telemedicine. Results will be confirmed by clinical observation during the Preparatory Period, but the SCID-5-PD, DDIS, and MINI will not be repeated.
Enrollment	Enrollment	1.5 hours/<br>2 to 14 days after Independent Rater Screening	Prior to enrolling: review all screening measures, medical history, discussion with outside providers and sponsor, and any clarification phone calls with participant. Visit is 1.5 hours to review eligibility and medical tapering plan. If enrolled, begin taper, (5 half-lives plus 7 days for stabilization). Adverse Event (AE) collection begins.

**Preparatory Period with Enrollment Confirmation**<br>~6 weeks (+5/-5 weeks)			
**Study Visit**		**Visit Duration/ Visit Timing**	**Brief Description of Events**
Preparatory Period	Preparatory Session 1	1.5 hours/<br>0 to 12 days after Enrollment	90-minute Preparatory Session. Target visit timing on tapering needs. If needed, schedule calls between Visits if indicated for tapering, safety, or further questions about medical history.
	Preparatory Session 2	1.5 hours/<br>2 to 21 days after Prep Session 1	90-minute Preparatory Session/ongoing assessment. If tapering is complete or not needed, check eligibility and schedule upcoming visits. If tapering is ongoing, schedule post taper call for ongoing assessment.

	Phone Call<br>End Taper	1 hour/<br>0 to 7 days after taper and stabilization end; up to 56 days after Enrollment; prior to Prep 3	If needed, confirm medication taper and stabilization is complete and participant is eligible for Baseline CAPS-5. Schedule Baseline CAPS-5 and Prep Session.
Baseline & Enrollment Confirmation	Baseline<br>CAPS-5	1.5 hours/<br>Post Prep Session 2 & Medication Taper; before Prep Session 3	CAPS-5, SDS, and DSP-I completed by an IR via telemedicine after taper is complete. CAPS scores sent ASAP to the therapy team/PI.<br><br>Due to possibilities of negative side effects of withdrawal from psychiatric medications, the Baseline CAPS-5 should be scheduled as close to the end of tapering as clinically appropriate so that participants who do not meet eligibility criteria can resume their previously prescribed medications as quickly as required for symptom management and participants who do meet criteria can be enrolled and treated as quickly as possible.
	Preparatory Session 3<br>& Enrollment Confirmation	3 hours (90-minute measures, 90-minute therapy)/<br>3 to 6 days after Baseline CAPS-5	Complete Baseline self-report measures. Complete 3-hour Preparatory Session (~90-minute measures, 90-minute therapy) and schedule Exp. Session 1. If enrollment is not confirmed, do not randomize; complete Study Termination.

**Treatment Period**<br>From Randomization to Integrative Session 3.3 12 weeks (-3/+4)			
**Study Visit**		**Visit Duration/**<br>**Visit Timing**	**Brief Description of Events**
Treatment 1	Randomization	.5 hours/<br>Within 2 week of Baseline CAPS-5/<br>24 to 48 hours before Exp. Session 1	Complete after enrollment and scheduling Exp. Session 1. Enter demographics in Medrio for use in randomization. The participant does not need to be present for this.
	Experimental Session 1	8 hours + overnight/<br>Within 2 weeks of Baseline CAPS-5	8 hours with overnight stay. Dose is 80 mg with supplemental half-dose of 40 mg unless contraindicated.
	Integrative Session 1.1	1.5 hours/<br>Morning after Experimental Session 1	90-minute Integrative Session, followed by phone check-ins that includes Since Last Visit C-SSRS assessment on Days 2, 4, 6, 7, 8, 10, 12, 14 post Experimental Session.
	Integrative Session 1.2	1.5 hours/<br>3 to 14 days after Experimental Session 1 at least 2 days after Integrative Session 1.1	Between 3 and 14 days after Experimental Session, a 90-minute Integrative Session is completed.
	Integrative Session 1.3	1.5 hours/<br>20 to 34 days after Experimental Session 1;<br>at least 2 days after Integrative Session 1.2;<br>1 to 7 days before Experimental Session 2	90-minute Integrative Session. Measures include LEC-5 and C-SSRS.
Treatment 2	Experimental Session 2	8 hours + overnight/<br>21 to 35 days after Experimental Session 1	The second Experimental Session lasts 8 hours with an overnight stay. Dose is 80 or 120 mg plus supplemental half-dose unless contraindicated.
	Integrative Session 2.1	1.5 hours/<br>Morning after Experimental Session 2	90-minute Integrative Session. Followed by phone check-ins that includes Since Last Visit C-SSRS assessment on Days 2, 4, 6, 7, 8, 10, 12, 14 post Experimental Session.
	Integrative Session 2.2	1.5 hours/<br>3 to 14 days after Experimental Session 2;<br>at least 2 days after Integrative Session 2.1	90-minute Integrative Session.
	Integrative Session 2.3	1.5 hours/<br>20 to 34 days after Experimental Session 2;<br>at least 2 days after Integrative Session 2.2<br>1 to 7 days before Experimental Session 3	90-minute Integrative Session . Measures include LEC-5 and C-SSRS.

Study Visit/ Visit #		Visit Duration/ Visit Timing	Brief Description of Events
Treatment 3	Experimental Session 3	8 hours + overnight/ 21 to 35 days after Experimental Session 2	The third Experimental Session lasts 8 hours with an overnight stay. Dose is 80 or 120 mg plus supplemental half-dose unless contraindicated.
	Integrative Session 3.1	1.5 hours/ Morning after Experimental Session 3	90-minute Integrative Session. Followed by phone check-ins that includes Since Last Visit C-SSRS assessment on Days 2, 4, 6, 7, 8, 10, 12, 14 post Experimental Session.
	Integrative Session 3.2	1.5 hours/ 3 to 14 days after Experimental Session 3; at least 2 days after Integrative Session 3.1	90-minute Integrative Session
	Integrative Session 3.3	1.5 hours/ 21 to 35 days after Experimental Session 3	90-minute Integrative Session. Measures include LEC-5 and C-SSRS.

**Follow-up Period and Study Termination** From Integrative Session 3.3 until Study Termination: 4 weeks (±2). After Integrative Session 3.3			
**Study Visit/ Visit #**		**Visit Duration/ Visit Timing**	**Brief Description of Events**
Study Termination	Study Termination	2 hours; 1 to 9 days after Integrative Session 3.3	Occurs 105 to 147 days post Baseline. Complete self-reported and safety measures; create an exit plan for participant. Invite participant to the extension study for LTFU. Let participant who completed the protocol know how they will be informed of unblinding and at that time, if in the placebo group, regardless of CAPS-5 scores, they will be offered the opportunity to enroll at no cost in an open-label safety extension study.

Qualified, blinded IRs selected based on availability from the IR Pool will perform the CAPS-5 assessments.

Dose Selection

This study will compare the effects of three blinded manualized Experimental Sessions of psychotherapy assisted by flexible doses of MDMA or placebo as described in the table below, along with associated non-drug preparatory and integrative psychotherapy. Similar MDMA doses to those proposed in this study have been safely used in previous Phase 2 studies sponsored by MAPS.

Dose Regimen of MDMA or Placebo

Experimental Session	Initial Dose	Supplemental Dose*	Min-Max Cumulative Dose
1	80 mg	40 mg	80 mg to 120 mg
2	80 or 120 mg	40 or 60 mg	80 mg to 180 mg
3	80 or 120 mg	40 or 60 mg	80 mg to 180 mg
		**Total Cumulative Dose**	240 mg to 480 mg

* Unless contraindicated

Protocol Objective

The overall objective of this study is to use standard clinical measures to confirm the efficacy and safety of manualized MDMA-assisted psychotherapy with a flexible dose of MDMA in reducing PTSD and associated symptoms in comparison to the same psychotherapy combined with an inactive placebo control.

Primary Objective

The primary objective of this study is to evaluate the efficacy of MDMA-assisted psychotherapy for PTSD compared to the identical psychotherapy with inactive placebo, based on change in CAPS-5 Total Severity.

Secondary Objective

The secondary objective of this study is to evaluate the efficacy of MDMA-assisted psychotherapy for PTSD compared with identical psychotherapy with inactive placebo in clinician-rated functional impairment, as measured by the mean change in Sheehan Disability Scale (SDS).

Safety Objectives

The overall safety objective is to assess differences between groups in severity, incidence and frequency of AEs, Treatment Emergent AEs (TEAEs), AEs of Special Interest (AESIs), and Serious Adverse Events (SAEs), concomitant medication use, suicidal ideation and behavior, and vital signs to support the package insert for MDMA-assisted psychotherapy.
The following safety objectives will evaluate the safety of MDMA-assisted psychotherapy compared to identical psychotherapy with inactive placebo:

1. Compare relative incidence of AEs during Experimental Sessions that may be indicative of a medical complication of the Investigational Medicinal Product (IMP), such as clinical signs and symptoms of chest pain, shortness of breath, or neurological symptoms or any other signs or symptoms that prompt additional vital sign measurements.
2. Compare relative incidence of AEs by severity.
3. Compare relative incidence of TEAEs, to determine relationship to the IMP based on relative incidence in the MDMA group.
4. Compare relative incidence of TEAEs by severity reported during an Experimental Session, 1 day, and 2 days after IMP administration.
5. Compare relative incidence of AESIs, defined as AEs specified in the protocol related to cardiac function and abuse liability.
6. Compare relative incidence of AEs by severity categorized as leading to discontinuation of IMP, resulting in death or hospitalization, and continuing at Study Termination.
7. Compare relative incidence of SAEs.
8. Compare relative incidence of psychiatric concomitant medications taken during an Experimental Session, 1 day, and 2 days after IMP administration.
9. Compare relative incidence of any psychiatric concomitant medications taken during the Treatment Period.
10. Compare relative incidence of positive or serious suicidal ideation and positive suicidal behavior assessed with the Columbia Suicide Severity Rating Scale (C-SSRS) by treatment group.

11. Compare mean changes in blood pressure, heart rate, and body temperature from pre-IMP administration to end of each Experimental Session by treatment group.

Recruitment and Participant Population

Participants will be recruited through print and internet advertisements, referrals from other treatment providers, and by word of mouth. The sponsor will monitor demographics on an ongoing basis and encourage diversity (representative of PTSD population) in enrollment of study participants by communicating with sites. Randomization will be stratified by clinical site.

Participants will be persons aged 18 or older, with a confirmed diagnosis of at least severe PTSD per the PCL-5 (PTSD Checklist for DSM-5) at Screening with symptoms present for at least 6 months. Participants would not be excluded for having more than one traumatic event or for having tried, not tolerated, or refused a selective serotonin reuptake inhibitor (SSRI) or serotonin norepinephrine reuptake inhibitor (SNRI) prescribed for PTSD. Participants with confirmed diagnosis of specific psychological and personality disorders will be excluded. Participants must be in good physical health and without major medical disorders that could affect the safety or tolerability of MDMA.

Statistical Analysis of Efficacy

The statistical power calculations were made by fitting a Mixed-Effect Model Repeated Measure (MMRM) model to CAPS-4 (Clinician-Administered PTSD Scale for DSM-4) data from the Phase 2 study MP1 to obtain covariance parameter estimates. The Phase 2 MP1 study followed a similar design with outcomes measured by CAPS-4 outcome scores, which allowed for estimation of within-subject covariance parameters. With a sample size of 50 participants per treatment group in the modified Intent-to-Treat (mITT) population, this study has 90% power to detect a treatment effect, using a two-sided test with an alpha of 0.0499. Additional participants may be enrolled, depending on sample size re-estimation by an independent DMC, with conditional power analysis conducted at a group-unblinded interim analysis for efficacy when 100 participants are enrolled and at least 60% of the blinded participants (N=60) have completed their final CAPS-5 assessment and terminated treatment. Under no circumstances will the number of participants enrolled (at V0) be reduced below the initial planned 100, in order to obtain additional safety data.

Primary Efficacy Analysis

All efficacy analyses will be based on the mITT analysis set. The primary treatment comparison will be made at a 2-sided, 0.0499 level of significance. The reduction from Baseline in CAPS-5 Total Severity Scores between groups will be compared at Primary Outcome with least squares means from an MMRM model. The mITT analysis set will include all randomized participants who receive IMP in at least one blinded Experimental Session and have at least one follow-up CAPS-5 assessment.

Protocol MAPP1
IND #063384

Original Protocol Version 1: 16 January 2017
Original Protocol Version 2: 07 June 2017
Amendment 1 Version 1: 26 February 2018
Amendment 2 Version 1: 12 September 2019
Amendment 3 Version 1: 13 November 2019
Amendment 4 Version 1: 22 May 2020

A Randomized, Double-Blind, Placebo-Controlled, Multi-Site Phase 3 Study of the Efficacy and Safety of Manualized MDMA-Assisted Psychotherapy for the Treatment of Severe Posttraumatic Stress Disorder

SPONSOR	Multidisciplinary Association for Psychedelic Studies (MAPS)<br>1115 Mission Street<br>Santa Cruz, CA 95060
SPONSOR REPRESENTATIVE	Rick Doblin, Ph.D.<br>Executive Director<br>MAPS
SPONSOR DELEGATE<br>&TRIAL ORGANIZER	MAPS Public Benefit Corporation<br>1115 Mission Street<br>Santa Cruz, CA 95060
SPONSOR DESIGNEE	Amy Emerson<br>Executive Director<br>MAPS Public Benefit Corporation
CLINICAL INVESTIGATOR	Multi-site Study
MEDICAL MONITOR	Michael C. Mithoefer, M.D.<br>Alia Lilienstein, M.D., M.P.H.
USE	In conjunction with relevant Food and Drug Administration (FDA), Health Canada (HC), and Israeli Ministry of Health (MOH) guidance

Table of Contents

List of Tables **13**
List of Figures **14**
1.0 List of Abbreviations **15**
2.0 Introduction **18**
2.1 Rationale 18
2.2 Background 18
2.2.1 PTSD 18
2.2.2 MDMA 20
2.2.3 MDMA-Assisted Psychotherapy for PTSD 20
2.2.4 Previous Clinical Experience with MDMA 21
3.0 Protocol Objectives **22**
3.1 Primary Objective 22
3.2 Key Secondary Objective 23
3.3 Safety Objectives 23
3.4 Exploratory Objectives 23
4.0 Eligibility Criteria **24**
4.1 Inclusion Criteria 24
4.2 Exclusion Criteria 25
4.3 Lifestyle Modifications 25
5.0 Protocol Design **26**
5.1 Study Design Overview 26
5.2 Planned Duration of Study 31
5.2.1 Interruptions and Accommodations Due to COVID-19 Pandemic or Any Other Unforeseen Emergency at Clinic Locations 32
5.3 Discontinuation and Completion Criteria 32
5.3.1 Complete or Evaluable Participants 32
5.3.2 Screen Failures 33
5.3.3 Pre-Randomization Early Terminations 33
5.3.4 Early Termination from the Study 33
5.3.5 Lost to Follow-up 34
5.4 End of Study Definition and Premature Discontinuation 35
5.5 Rationale of Dose Selection 35
6.0 Psychotherapy **36**
6.1 Description of Therapeutic Method 36
6.2 Therapy Team Qualifications 36
6.3 Training 37
6.4 Adherence to Therapeutic Method 37
7.0 Measures and Reliability **37**
7.1 Primary Outcome Measure and Reliability 39
7.1.1 CAPS-5 (Clinician-Administered PTSD Scale for DSM-5) 39
7.2 Secondary Measure 39
7.2.1 SDS (Sheehan Disability Scale) 39
7.3 Safety Measures 39
7.3.1 C-SSRS (Columbia Suicide Severity Rating Scale) 39
7.4 Screening Measures and Reliability 40
7.4.1 MINI (Mini-International Neuropsychiatric Interview) 40
7.4.2 SCID-5-PD (Structured Clinical Interview for DSM-5 for Personality Disorders) 40
7.4.3 LEC-5 (Life Events Checklist for DSM-5) 40
7.4.4 PCL-5 (PTSD Checklist) 40

7.4.5 DDIS (Dissociative Disorders Interview Schedule for DSM-5) 41
7.5 Exploratory Measures 41
7.5.1 DSP-I (The Dissociative Subtype of PTSD Interview) 41
7.5.2 ACE (Adverse Childhood Experience Questionnaire) 41
7.5.3 BDI-II (Beck Depression Inventory II) 41
7.5.4 CPGS (Chronic Pain Grade Scale) 42
7.5.5 EQ-5D-5L (EuroQol Five Dimensions-Five Levels Questionnaire) 42
7.5.6 IASC (Inventory of Altered Self Capacities) 42
7.5.7 IPF (Inventory of Psychosocial Functioning) 42
7.5.8 SCS (Self-Compassion Scale) 43
7.5.9 TAS-20 (Toronto Alexithymia Scale) 43
7.5.10 AUDIT (Alcohol Use Disorders Identification Test) 43
7.5.11 DUDIT (Drug Use Disorders Identification Test) 43
7.5.12 SRNU (Self-reported Nicotine Use) 44
7.5.13 EAT-26 (Eating Attitudes Test) 44
7.5.14 HPQSF (Health and Work Performance Absenteeism and Presenteeism Short Form) 44
7.5.15 UFEC (Utilization of Facility-based and Emergent Care) 44
8.0 Study Procedures 44
8.1 Screening Period 45
8.1.1 Screening 45
8.1.2 Independent Rater Screening 47
8.1.3 Enrollment 47
8.2 Preparatory Period with Enrollment Confirmation 48
8.2.1 Preparatory Sessions 1 and 2 48
8.2.2 Baseline: CAPS-5 by Independent Rater 49
8.2.3 Baseline: Preparatory Session 3 & Enrollment Confirmation 49
8.3 Treatment Period 50
8.3.1 Experimental Sessions 50
8.3.2 Telephone Contact After Experimental Sessions 52
8.3.3 Integrative Sessions 53
8.3.4 Independent Rater Assessments 54
8.4 Follow-up Period and Study Termination 54
8.4.1 Follow-up Period 54
8.4.2 Study Termination 54
8.4.2.1 Extension Studies 55
8.4.2.2 Exit Plan 55
9.0 Investigational Product 56
9.1 Description of Active Compounds 56
9.1.1 Doses 56
9.1.2 Dose Modifications 56
9.1.3 Stability 56
9.2 Handling 56
9.2.1 Encapsulation, Packaging, and Labeling 56
9.2.2 Accountability 57
9.2.3 Storage 57
9.2.4 Administration 57
9.2.5 Treatment Compliance 57
9.3 Randomization and Participant Numbering 57
9.4 Blinding and Bias Minimization 58
10.0 Risks 59
10.1 Non-drug Related Risks 59

10.1.1 Medical Assessments 59
10.1.2 PTSD, Suicide Risk, and Psychotherapy 59
10.1.3 Recorded Content 60
10.2 Risks of Receiving MDMA 61
10.2.1 High Level Risks 61
10.2.2 Medium Level Risks 61
10.2.2.1 Cardiovascular and Cerebrovascular Risks and Mitigation 61
10.2.2.2 Psychological Risks and Mitigation 62
10.2.3 Low Level Risks 64
10.2.3.1 Thermoregulatory Risks and Mitigation 64
10.2.3.2 Osmoregulatory Risk and Mitigation 64
10.2.3.3 Genotoxicity Risk and Mitigation 64
10.2.3.4 Reproductive and Developmental Risks and Mitigation 64
10.2.4 Minimal Risks 65
10.2.4.1 Common AEs 65
10.2.4.2 Potential Neurotoxicity Associated with Ecstasy Use 65
10.2.4.3 Abuse Liability 65
11.0 Safety 66
11.1 Adverse Events 66
11.1.1 Adverse Events of Special Interest 67
11.1.2 Serious Adverse Events 67
11.2 Other Significant Events 68
11.3 Pregnancy 68
11.3.1 Definition of Childbearing Potential 68
11.3.2 Contraception Guidelines 69
11.3.3 Follow-up Requirements 69
11.4 Medical Monitor 69
12.0 Concomitant Medications 70
12.1 Tapering Instructions 70
12.2 Allowed Concomitant Medications 70
12.3 Prohibited Medications 71
13.0 Clinical Laboratory Assessments 71
14.0 Statistical Considerations 72
15.0 Study Governance 72
15.1 Ethics 72
15.1.1 Financial Disclosure 73
15.1.2 Informed Consent 73
15.2 Study Monitoring, Auditing, and Documentation 74
15.2.1 Source Records 74
15.3 Confidentiality 74
15.4 Costs to Participants 75
15.5 Treatment and Compensation for Study Related Injury 75
15.6 Record Retention 76
15.7 Publication Policy 76
16.0 References 77
Appendix A: Sub-study without Overnight Stays 83

List of Tables

Table 1: Study Design Overview....28
Table 2: Primary Outcome (CAPS-5) Data Collection by Visit 38
Table 3: Dose Regimen of MDMA or Placebo 36
Table 4: Protocol Objectives and Assessment Tools....37
Table 5: Time and Events-Study Procedures....52
Table 6: Time and Events-Study Measures....55
Table 7: Schedule of Procedures for Experimental Sessions 50

List of Figures

No table of figures entries found.

1.0 List of Abbreviations

°C	Degrees Celsius
A:G	Albumin:Globulin
ACE	Adverse Childhood Experiences Questionnaire
ADHD	Attention Deficit/Hyperactivity Disorder
AE	Adverse Event
AESI	Adverse Event of Special Interest
ALT	Alanine Aminotransferase
AMI	Acute Myocardial Infarction
API	Active Pharmaceutical Ingredient
AST	Aspartate Aminotransferase
AUDIT	Alcohol Use Disorders Identification Test
BDI-II	Beck Depression Inventory-II
BMI	Body Mass Index
BP	Blood Pressure
BUN	Blood Urea Nitrogen
CAPS-4	Clinician-Administered PTSD Scale for DSM-4
CAPS-5	Clinician-Administered PTSD Scale for DSM-5
CBC	Complete Blood Count
%CDT	%Carbohydrate-deficient Transferrin
CMC	Chemistry Manufacturing and Control
COVID-19	Coronavirus Disease 2019
CPGS	Chronic Pain Grade Scale
CRA	Clinical Research Associate
CRP	C-Reactive Protein
C-SSRS	Columbia-Suicide Severity Rating Scale
DDIS	Dissociative Disorders Interview Schedule
DID	Dissociative Identity Disorder
dIGPP	Cohen’s d Independent Groups Pre-test Post-test
DMC	Data Monitoring Committee
DSM-5	Diagnostic and Statistical Manual of Mental Disorders, 5th edition
DSP-I	Dissociative Subtype of PTSD Interview
DUDIT	Drug Abuse Disorders Identification Test
EAT-26	Eating Attitudes Test
ECG	Electrocardiogram
eCRF	Electronic Case Report Form
ECT	Electroconvulsive Therapy
ED	Emergency Department
EDC	Electronic Data Capture
EMDR	Eye Movement Desensitization and Reprocessing
EMS	Emergency Medical Services
ePRO	Electronic Participant Reported Outcome
EQ-5D-5L	EuroQol Five Dimensions-Five Levels Questionnaire
FDA	Food and Drug Administration
GCP	Good Clinical Practice
GMP	Good Manufacturing Practice
HCV	Hepatitis C Virus
HIV	Human Immunodeficiency Virus
HIPAA	Health Insurance Portability and Accountability
HPA	Hypothalamic-pituitary-adrenal

HPQSF	Health and Work Performance Absenteeism and Presenteeism Short Form
IASC	Inventory of Altered Self-Capacities
IB	Investigator's Brochure
ICD	International Classification of Disease
ICF	Informed Consent Form
ICH	International Conference on Harmonisation
IND	Investigational New Drug
IMP	Investigational Medicinal Product
IPF	Inventory of Psychosocial Functioning
IR	Independent Rater
IRDB	Independent Rater Database
IRB	Institutional Review Board
ISF	Investigator Site File
ITT	Intent-to-Treat
IUD	Intrauterine Device
IUS	Intrauterine Hormone-releasing System
IWRS	Interactive Web Randomization System
kg	Kilogram
LEC-5	Life Events Checklist
LTFU	Long-term Follow-up
MAPS	Multidisciplinary Association for Psychedelic Studies
MAOI	Monoamine Oxidase Inhibitor
MCH	Mean Corpuscular Hemoglobin
MCHC	Mean Corpuscular Hemoglobin Concentration
MCV	Mean Corpuscular Volume
MDMA	3,4-methylenedioxymethamphetamine
mg	Milligram
mITT	Modified Intent-to-Treat
mmHg	Milligrams of Mercury
MMRM	Mixed Model Repeated Measure
MPBC	MAPS Public Benefit Corporation
ms	Millisecond
PCL-5	PTSD Checklist for DSM-5
PTCA	Percutaneous Transluminal Coronary Angioplasty
PTSD	Posttraumatic Stress Disorder
RACT	Risk Assessment and Categorization Tool
RBC	Red Blood Cell
RDW	Red Cell Distribution Width
SAE	Serious Adverse Event
SCID-5-PD	Structured Clinical Interview for DSM-5 Personality Disorders
SCID-5-SPQ	SCID-5 Self-report Personality Questionnaire
SCS	Self-compassion Scale
SDS	Sheehan Disability Scale
SGOT	Serum Glutamic Oxaloacetic Transaminase
SNRI	Serotonin-norepinephrine Reuptake Inhibitor
SRNU	Self-reported Nicotine Use
SSRI	Selective serotonin reuptake inhibitor
TAS-20	Toronto Alexithymia Scale
TEAE	Treatment Emergent Adverse Event
TSH	Thyroid-stimulating Hormone
UFEC	Utilization of Facility-based and Emergent Care
U.S.	United States

VA	U.S. Department of Veterans Affairs
VAS	Visual Analog Scale
WBC	White Blood Cell
WHO	World Health Organization

2.0 Introduction

The Multidisciplinary Association for Psychedelic Studies (MAPS) is a non-profit research and educational organization working as a clinical trial sponsor to obtain marketing approval for the prescription use of 3,4-methylenedioxymethamphetamine (MDMA) as an adjunct to psychotherapy in patients with posttraumatic stress disorder (PTSD). Controlled Phase 1 studies, nonclinical studies, and investigator-initiated studies formed the basis for the Clinical Development Program of MDMA under Investigational New Drug (IND) #063384. MAPS-sponsored studies are implemented through MAPS' wholly owned subsidiary and delegate, the MAPS Public Benefit Corporation (MPBC).

2.1 Rationale

PTSD is a serious debilitating disorder that negatively impacts a person's daily life. MDMA has been shown to reduce defenses and fear of emotional injury, enhance communication, and increase empathy. MDMA may enhance fear extinction learning in humans. These subjective effects of MDMA create a productive psychological state that enhances the therapeutic process for the treatment of PTSD and other anxiety disorders. This is supported by data from an international series of Phase 2 pilot studies of MDMA-assisted psychotherapy conducted by the sponsor that provide preliminary evidence that chronic PTSD, independent of cause, is treatable with two to three sessions of MDMA-assisted psychotherapy and associated non-drug preparatory and integrative psychotherapy. This pivotal Phase 3 study is intended to confirm the efficacy and safety of manualized MDMA-assisted psychotherapy as a treatment for PTSD.

The results from multiple independent studies in Phase 2 efficacy analyses demonstrate superiority of MDMA-assisted psychotherapy over psychotherapy with placebo and low dose MDMA. The acceptable risk-benefit ratio in early trials justifies confirmation in a Phase 3 program. To further assess the efficacy and safety of this treatment for participants with at least severe PTSD, the sponsor is conducting this pivotal Phase 3 randomized, placebo-controlled, two-arm, double-blind, multi-site study which will include an interim analysis of CAPS-5 data for sample size re-estimation. This novel treatment package consists of three once-monthly Experimental Sessions of psychotherapy combined with either a flexible dose of MDMA or inactive placebo, along with non-drug preparatory and integrative psychotherapy. The Primary Outcome measure, the Clinician Administered PTSD Scale (CAPS-5), evaluates changes in PTSD symptom severity and is assessed by a blinded centralized Independent Rater (IR) pool.

2.2 Background

2.2.1 PTSD

PTSD is a serious debilitating disorder associated with increased mortality and cardiometabolic morbidity. PTSD is a stress-related psychiatric condition that may occur following a traumatic event such as war, disaster, sexual abuse, violence, terrorism, and accidents. The four main symptom categories described in the Diagnostic and Statistical Manual of Mental Disorders, 5th edition (DSM-5), include arousal and reactivity, avoidance of triggers, negative thoughts and feelings, and intrusive thoughts and nightmares. PTSD negatively impacts a person's daily life, resulting in fractured relationships, inability to maintain employment, diminished cognitive and psychosocial functioning, substance abuse, high-cost healthcare utilization, and increased depression and suicide risk. People who suffer from PTSD often relive the experience through nightmares and flashbacks, have poor sleep quality, and feel detached or estranged. Confronting overwhelming internal distress and frightening external environments can also lead to high levels of depersonalization and derealization, which led clinicians to identify a dissociative subtype of

PTSD in the DSM-5. Adaptations in normal brain function have been observed in imaging studies of patients with PTSD that underlie alterations in emotional processing and regulation, cognition, and many aspects of behavior, though clinical symptoms and changes in brain activity are not homogenous across patients [1]. The dissociative subtype occurs in 12 to 30% of people with PTSD and is characterized by detachment and emotional numbing and visualized in the brain as overmodulation of affect mediated by midline prefrontal inhibition of limbic regions, while the non-dissociative subtype presents symptoms of hyperarousal and re-experiencing, an emotional undermodulation mediated by failure of prefrontal inhibition of the same limbic regions [2, 3]. Patients suffering from the dissociative subtype of PTSD typically have early childhood trauma and appear to be particularly difficult to treat, with mixed response to existing evidence-based treatments.

Approximately 7% of the population in the United States (U.S.) will have PTSD sometime in their life, but this figure jumps to 10.8% to 13% of veterans with combat experience [4]. For soldiers returning from Iraq and Afghanistan, the incidence of PTSD is 17.1% with 400,000 to 500,000 U.S. Iraq/Afghanistan veterans reportedly having PTSD. In 2004, the Defense Department and U.S. Department of Veterans Affairs (VA) spent $4.3 billion on PTSD disability payments to approximately 215,000 veterans [5]. In 2012 alone the VA spent $294 million and $3 billion, respectively, on care for veterans with the disorder and disability payments, even with this funding the demand for services far outreached the availability of VA doctors and services. As of June 30, 2016, more than 868,000 veterans with a diagnosis of PTSD were receiving disability compensation for service-connected mental disorders, with an estimated cost of about $17 billion per year [6]. There are an estimated 20 to 22 suicides a day by veterans [7].

Available PTSD treatments, including medications and therapy, effectively treat only a fraction of people who try them for adequate dose and duration. This indicates a need to develop treatments targeting durable remission of PTSD. The Food and Drug Administration (FDA) has approved only two pharmacotherapies for PTSD, both of which are selective serotonin reuptake inhibitors (SSRIs). Paroxetine and sertraline (Paxil and Zoloft) both demonstrated statistically significant superiority over placebo on the CAPS in 12-week confirmatory clinical trials with daily dosing, but some studies were less effective in treating combat-related PTSD and sertraline demonstrated gender differences with minimal efficacy in men [8-10]. PTSD rarely remits after 12 weeks of SSRIs, and many patients who are placed on maintenance treatment experience partial relief of symptoms, which fully return upon discontinuation of treatment. Adverse effects of maintenance SSRI treatment that contribute to discontinuation include sexual dysfunction, weight gain, and sleep disturbance. Variable SSRI treatment outcomes have led to recommendations of trauma-focused psychotherapy as routine first-line treatment by the VA’s National Center for PTSD in the U.S., as well as by the World Health Organization (WHO). An extensive list of medications, namely antipsychotics, anxiolytics, antidepressants, and sleep aids, are frequently prescribed off-label but have only small effect sizes in reducing PTSD symptoms. PTSD brings a high public burden, both economically and socially, by increased use of health and social services, lost wages, and disability payments [11, 12]. Given the chronicity of PTSD, low compliance evidenced by high dropouts, and limited recovery with current medications contributing to serious outcomes, PTSD patients suffer from unmet medical need.

One treatment approach is to develop medications and/or psychotherapeutic treatments that may indirectly decrease or eliminate the neurochemical pathologies underlying the chronic hyperarousal and dysregulation of the hypothalamic-pituitary-adrenal (HPA) axis associated with PTSD. Cognitive behavioral therapies, particularly prolonged exposure and cognitive processing therapy, are considered among the most effective psychotherapies. Other methods such as psychodynamic therapy and eye movement desensitization and reprocessing (EMDR) have also proven to be effective in treating some symptoms of PTSD [13, 14], although some patients may

need more than one type of treatment to reduce or resolve those symptoms. A meta-analysis concluded that all "bona fide" psychotherapies, including those listed above, are similarly effective with PTSD [15]. In the past decade, there has been a growing amount of research into medications and other methods that may augment the effectiveness of psychotherapy for PTSD (see [16] for a review). Examples of this are virtual reality-assisted exposure therapy [17, 18] and D-cycloserine-assisted psychotherapy [19]. MDMA-assisted psychotherapy is another such approach.

2.2.2 MDMA

MDMA is a ring-substituted phenylisopropylamine derivative invented by the Merck pharmaceutical company in 1912 [20, 21]. Similar to SSRIs, MDMA binds to the serotonin transporter, but has additional effects on carrier-mediated release and reuptake inhibition of norepinephrine, and to a lesser extent in humans, dopamine [22-28]. MDMA also increases levels of affiliative neurohormones oxytocin and vasopressin, which increases trust and attenuates reactivity to threatening cues, and some researchers have suggested a role for oxytocin in treating PTSD. The indirect effects of MDMA on central and peripheral neurohormone release contribute to a novel mechanism that may help regulate the HPA axis, which would treat the core psychopathology of PTSD for a durable remission.

Onset of MDMA effects occurs ~0.5 to 1 hour after oral administration, and peak effects occur 1.25 to 2 hours after the initial dose. Effects of the initial dose last 3 to 6 hours, which is extended to 5 to 8 hours with a supplemental half-dose administered 1.5 to 2 hours post initial dose. Orally administered doses of MDMA have a half-life of 7 to 9 hours in humans. Unlike approved PTSD medications, therapeutic effects of MDMA have a rapid-onset and do not require daily dosing or a steady state in the blood to be effective. Thus, the effects of MDMA are distinct from and go well beyond anxiolytics and SSRIs. Furthermore, there is no evidence that MDMA creates a physical dependency, as benzodiazepines do. Previous studies of polydrug users have found a small percentage of people exhibit problematic use of Ecstasy (material represented as containing MDMA) [29, 30]. Studies of regular or problematic Ecstasy users indicate that on average, regular use occurs no more often than once a week [31]. Hence, MDMA may have moderate abuse potential. See the Investigator's Brochure (IB) for a more detailed explanation.

2.2.3 MDMA-Assisted Psychotherapy for PTSD

Many psychotherapies for PTSD involve the induction and extinction of abnormal autonomic responses through revisiting traumatic experiences in psychotherapy with an appropriate level of emotional engagement [14]. To be effective, exposure must be accompanied by a degree of emotional engagement or "fear activation" while avoiding dissociation or overwhelming emotion [32]. This has been referred to as working within the "optimal arousal zone" or "window of tolerance" [33-35].

The combined neurobiological effects of MDMA increase compassion, reduce defenses and fear of emotional injury, and enhance communication and introspection. MDMA produces anxiolytic and prosocial effects, which counteract avoidance and hyperarousal in the context of therapy. PTSD increases amygdala activity, causing heightened encoding of fearful memories and decreasing blood flow in the prefrontal cortex. In contrast, MDMA acutely decreases activity in the amygdala [36], and there is some indication that MDMA may increase activity in the prefrontal cortex [37]. Brain imaging after MDMA indicates less reactivity to angry facial expressions and greater reward in happy faces [36]. This action is compatible with its reported reduction in fear or defensiveness, and is in contrast to the stimulation of the amygdala observed in animal models of conditioned fear, a state similar to PTSD [38-40]. The reduction in stress-

induced activation of the amygdala may be supported and enhanced by interacting with the therapy team during and after the MDMA experience. The subjective effects of MDMA create a productive psychological state that enhances the therapeutic process. MDMA is capable of inducing unique psychopharmacological effects, including decreased fear and increased wellbeing, sociability, interpersonal trust, acceptance of self and others, and ability to address these issues without extreme disorientation or ego loss due to alert state of consciousness. These factors taken together can provide the opportunity for a corrective emotional experience.

A combined treatment of MDMA and psychotherapy may be especially useful for treating PTSD because MDMA can attenuate the fear response of a perceived threat to one's emotional integrity and decrease defensiveness without blocking access to memories or preventing a deep and genuine experience of emotion [41-44]. Elimination of these conditioned fear responses can lead to more open and comfortable communication about past traumatic events and greater access to information about them [45]. Participants are able to experience and express fear, anger, and grief with less likelihood of feeling overwhelmed by these emotions. MDMA seems to engender internal awareness that even painful feelings that arise are an important part of the therapeutic process. In addition, feelings of empathy, love, and deep appreciation often emerge, along with a clearer perspective of the trauma as a past event, a more accurate perspective about its significance, and a heightened awareness of the support and safety that exists in the present. As a result, MDMA-assisted psychotherapy may enable the participants to restructure their intra-psychic realities and develop a wider behavioral and emotional repertoire with which to respond to anxiogenic stimuli.

The therapeutic method is described in further detail in the Treatment Manual of MDMA-Assisted Psychotherapy, which the sites and therapy teams will be trained on prior to the study.

2.2.4 Previous Clinical Experience with MDMA

MDMA-assisted psychotherapy is a novel treatment package that combines psychotherapeutic techniques with the administration of MDMA as a pharmacological adjunct intended to enhance certain aspects of psychotherapy. Chemists Shulgin and Nichols were the first to report on the effects of MDMA in humans [46], with 80 to 160 milligrams (mg) MDMA required to produce desired subjective effects in humans [46, 47]. MDMA was found to robustly influence human emotional status in a unique way [46] without adversely affecting physiological functions or perception, such as visual perception or cognition [48-51]. In the 1970s, psychotherapists used MDMA-assisted psychotherapy to treat psychological disorders, including anxiety [52]. Legal therapeutic use continued until its placement on the U.S. list of Schedule 1 substances in 1985 [42, 45, 53]. An estimated 500,000 doses of MDMA were administered during psychotherapy and personal growth sessions in North America prior to its scheduling [42, 54]. A few uncontrolled human studies of MDMA assessing safety in a therapeutic setting occurred in the 1980s [55, 56].

Controlled human studies for clinical development of MDMA commenced in the mid-1990s with a MAPS-funded investigator-initiated Phase 1 dose-response safety study [57, 58]. Starting in 2000 in Spain, MAPS funded a Phase 2 investigator-initiated dose-response effect and safety pilot study in participants with PTSD that was terminated early due to political pressure. This study enrolled six participants, with four receiving a single session of MDMA-assisted psychotherapy without any safety concerns and with some PTSD symptom reduction [44]. These studies formed the basis of clinical experience with MDMA prior to studies subsequently conducted under a MAPS IND.

Under IND #063384, MAPS initiated an international series of Phase 2 clinical trials to develop the medical use of MDMA-assisted psychotherapy for patients with chronic, at least moderate

PTSD (CAPS-4 score: 50+), with at least 6 months of symptoms. Participants were not excluded for having more than one traumatic event, or for having tried, not tolerated, or refused an SSRI or serotonin-norepinephrine reuptake inhibitor (SNRI) prescribed for PTSD. Outcomes from six Phase 2 studies with evaluable data have been promising and have generated a range of methodological information for the design of future studies.

Results from two Phase 2 studies have been published: one study in the U.S. with a long-term follow-up (LTFU) conducted an average of 3.8 years after the final MDMA-assisted psychotherapy session (MP1) [43, 59] and one in Switzerland (MP2) [60, 61]. MP1 was followed by a small open-label extension study examining the treatment of relapse in three participants with a single MDMA-assisted psychotherapy treatment and a 12-month follow-up (MP1E2). Three additional studies have completed treatments (MP8, MP9, MP12) and two international studies were terminated early for logistical reasons with partial datasets (MP3, MP4). These studies tested a range of designs, such as a placebo control (MP1, MP4), low dose MDMA comparator control (MP2, MP9), and three-arm dose response studies (MP8, MP12). MP4 was terminated early due to delays in regulatory approval and enrollment timelines, with available efficacy data presented without a formal analysis. MP3 was terminated early by the sponsor due to inadequate data collection procedures at the site and insufficient therapy team training; efficacy data are not available for these reasons (MP3 is excluded from Phase 2 data).

Intent-to-treat (ITT) analysis of primary efficacy and safety data from six MAPS-sponsored MDMA PTSD Phase 2 clinical trials worldwide (MP1, MP2, MP4, MP8, MP9, MP12) consisting of 107 blinded participants with chronic PTSD was completed in 2016. In these studies, PTSD, independent of cause, appears treatable with a two to three-session treatment package of MDMA-assisted psychotherapy. As of October 1, 2018, with 248 individuals exposed to MDMA in the sponsor's development program across various indications and at least 1,286 participants in MDMA research studies conducted without sponsor support, the sponsor has observed an acceptable risk-benefit ratio for MDMA-assisted psychotherapy. Across Phase 2 studies, 75 mg to 125 mg MDMA was statistically superior to 0 mg to 40 mg MDMA based on a t-test of difference in CAPS-4 severity scores from Baseline, 2 months after two blinded Experimental Sessions ($p<0.001$). The dropout rate across studies was 7.5% (8 of 107). Large between-groups effect size estimates (0.9), initial indications of efficacy, and favorable safety outcomes support expanding the research initiative to encompass a larger sample of participants with PTSD in a Phase 3 program.

A comprehensive review of MDMA research can be found in the IB supplied by the sponsor. This document should be reviewed prior to initiating the protocol.

3.0 Protocol Objectives

The overall objective of this study is to use standard clinical measures to confirm the efficacy and safety of manualized MDMA-assisted psychotherapy with a flexible dose MDMA in reducing PTSD and associated symptoms in comparison to the same psychotherapy combined with an inactive placebo control.

3.1 Primary Objective

The primary objective of this study is to evaluate the efficacy of MDMA-assisted psychotherapy for PTSD compared to identical psychotherapy with inactive placebo, as measured by the reduction in CAPS-5 Total Severity Scores.

3.2 Key Secondary Objective

The key secondary objective of this study is to evaluate the efficacy of MDMA-assisted psychotherapy for PTSD compared to identical psychotherapy with inactive placebo in clinician-rated functional impairment, as measured by the mean change in Sheehan Disability Scale (SDS) item scores.

3.3 Safety Objectives

The overall safety objective is to assess differences between groups in severity, incidence and frequency of Adverse Events (AEs), Treatment Emergent AEs (TEAEs), AEs of Special Interest (AESIs), and Serious Adverse Events (SAEs), concomitant medication use, suicidal ideation and behavior, and vital signs to support the package insert for MDMA-assisted psychotherapy. The following safety objectives will evaluate the safety of MDMA-assisted psychotherapy compared to identical psychotherapy with inactive placebo:

1. Compare relative incidence of AEs during Experimental Sessions that may be indicative of a medical complication of the Investigational Medicinal Product (IMP) such as clinical signs and symptoms of chest pain, shortness of breath, or neurological symptoms or any other signs or symptoms that prompt additional vital sign measurements.
2. Compare relative incidence of AEs by severity.
3. Compare relative incidence of TEAEs, to determine relationship to the IMP based on relative incidence in the MDMA group.
4. Compare relative incidence of TEAEs by severity reported during an Experimental Session, 1 day, and 2 days after IMP administration.
5. Compare relative incidence of AESIs, defined as AEs specified in the protocol related to cardiac function and abuse liability.
6. Compare relative incidence of AEs by severity categorized as leading to discontinuation of IMP, resulting in death or hospitalization, and continuing at Study Termination.
7. Compare relative incidence of SAEs.
8. Compare relative incidence of concomitant medications taken during an Experimental Session, 1 day, and 2 days after IMP administration.
9. Compare relative incidence of psychiatric concomitant medications taken during the Treatment Period.
10. Compare relative incidence of positive or serious suicidal ideation and positive suicidal behavior assessed with the Columbia Suicide Severity Rating Scale (C-SSRS) by treatment group.
11. Compare mean changes in blood pressure, heart rate, and body temperature from pre-IMP administration to end of each Experimental Session by treatment group.

3.4 Exploratory Objectives

These objectives may be explored to characterize participants receiving MDMA-assisted psychotherapy in comparison to those receiving psychotherapy with inactive placebo to support the primary objective:

1. Explore the effect of presence of secondary traumatic stressors (LEC-5) on the CAPS-5 Total Severity analyses.

2. Explore changes in PTSD symptom clusters of re-experiencing, avoidance, negative alterations in cognition and mood, and hyperarousal, as measured by changes in CAPS-5 subscale scores.
3. Characterization of CAPS-5 Total Severity score to explore onset of treatment efficacy with a descriptive time course plot.
4. Explore the effect of adverse childhood experiences (ACE) on the CAPS-5 Total Severity analyses.
5. Explore differences between treatment groups in:
    a. Dissociative symptoms associated with PTSD (DSP-I)
    b. Depression (BDI-II)
    c. Chronic pain (CPGS)
    d. Quality of life (EQ-5D-5L)
    e. Functioning in relation to self and others (IASC)
    f. Psychosocial functioning (IPF)
    g. Self-compassion (SCS)
    h. Alexithymia (TAS-20)
    i. Addictive behaviors including: alcohol use (AUDIT), drug use (DUDIT), and nicotine use (SRNU)
    j. Eating habits (EAT-26)
    k. Workplace productivity (HPQSF)
    l. Healthcare utilization (UFEC)

4.0 Eligibility Criteria

4.1 Inclusion Criteria

At the completion of Screening, participants must meet all eligibility criteria (except Inclusion Criterion #13) and agree to all lifestyle modifications to be enrolled. Each participant will then enter the Preparatory Period which includes medication tapering, if needed, and non-drug Preparatory Sessions. The Preparatory Period ends with Enrollment Confirmation. A participant's enrollment will be confirmed if they have completed medication tapering, have a confirmed PTSD diagnosis per the CAPS-5 assessment, continue to agree to all lifestyle modifications, and continue to meet all eligibility criteria.

Potential participants are eligible to enroll in the protocol if they:

1. Are at least 18 years old.
2. Are fluent in speaking and reading the predominantly used or recognized language of the study site.
3. Are able to swallow pills.
4. Agree to have study visits recorded, including Experimental Sessions, Independent Rater assessments, and non-drug psychotherapy sessions.
5. Must provide a contact (relative, spouse, close friend or other support person) who is willing and able to be reached by the investigators in the event of a participant becoming suicidal or unreachable.
6. Must agree to inform the investigators within 48 hours of any medical conditions and procedures.
7. If of childbearing potential, must have a negative pregnancy test at study entry and prior to each Experimental Session, and must agree to use adequate birth control through 10 days after the last Experimental Session. Adequate birth control methods include intrauterine device (IUD), injected, implanted, intravaginal, or transdermal hormonal methods, abstinence, oral hormones plus a barrier contraception,

vasectomized sole partner, or double barrier contraception. Two forms of contraception are required with any barrier method or oral hormones (i.e. condom plus diaphragm, condom or diaphragm plus spermicide, oral hormonal contraceptives plus spermicide or condom). Not of childbearing potential is defined as permanent sterilization, postmenopausal, or assigned male at birth.

8. Agree to the following lifestyle modifications (described in more detail in Section 4.3 Lifestyle Modifications): comply with requirements for fasting and refraining from certain medications prior to Experimental Sessions, not participate in any other interventional clinical trials during the duration of the study, remain overnight at the study site after each Experimental Session and be driven home after, and commit to medication dosing, therapy, and study procedures.

Medical History

9. At Screening, meet DSM-5 criteria for current PTSD with a symptom duration of 6 months or longer.
10. May have well-controlled hypertension that has been successfully treated with anti-hypertensive medicines, if they pass additional screening to rule out underlying cardiovascular disease.
11. May have asymptomatic Hepatitis C virus (HCV) that has previously undergone evaluation and treatment as needed.
12. May have a history of or current Diabetes Mellitus (Type 2) if additional screening measures rule out underlying cardiovascular disease, if the condition is judged to be stable on effective management, and with approval by the Medical Monitor.
13. May have hypothyroidism if taking adequate and stable thyroid replacement medication.
14. May have a history of, or current, glaucoma if approval for study participation is received from an ophthalmologist.

4.2 Exclusion Criteria

Potential participants are ineligible to enroll in the protocol if they:

1. Are not able to give adequate informed consent.
2. Have any current problem which, in the opinion of the investigator or Medical Monitor, might interfere with participation.
3. Weigh less than 48 kilograms (kg).
4. Are pregnant or nursing, or are of childbearing potential and are not practicing an effective means of birth control.

4.3 Lifestyle Modifications

All participants must agree to the following lifestyle modifications at enrollment and throughout the duration of the study. Participants are eligible to enroll in the study if they:

- Are willing to commit to medication dosing, psychotherapy sessions, follow-up sessions, completing evaluation instruments, and all necessary telephone contact.
- Agree to not participate in any other interventional clinical trials during the duration of this study.

Leading up to Experimental Sessions

- Agree to take nothing by mouth except alcohol-free liquids after 12:00 A.M. (midnight) the evening before each Experimental Session.
- Refrain from the use of any psychoactive medication not approved by the research team from Baseline through Study Termination.
- Agree not to use caffeine or nicotine for 2 hours before and at least 6 hours after the initial dose during each Experimental Session.
- Are willing to comply with medication requirements per protocol (refer to Section 12.0 Concomitant Medications). Medications will only be discontinued after enrollment per clinical judgment of the site physician in consultation with the prescribing physician.
- Are able to decrease dose of allowable opiates (per Section 12.0 Concomitant Medications), if used for pain management, leading up to the Experimental Session in order to avoid taking the medication for at least 12 hours prior to the initial IMP administration and 24 hours after. During this period, the participant will be allowed to take the medication if needed for intolerable pain flare-ups.
- Agree that, for 1 week preceding each Experimental Session to refrain from:
    - Taking any herbal supplement (except with prior approval of the research team).
    - Taking any nonprescription medications (with the exception of non-steroidal anti-inflammatory medications or acetaminophen unless with prior approval of the research team).
    - Taking any prescription medications (with the exception of birth control, thyroid hormones, or other medications approved by the research team).

Post Experimental Session (for exceptions, see Appendix A sub-study)

- Are willing to remain overnight at the study site after each Experimental Session until after the Integrative Session the next morning.
- Are willing to be driven home on the morning after the Experimental Sessions after the Integrative Session, either by a driver arranged by the participant, site personnel or taxi.

5.0 Protocol Design

5.1 Study Design Overview

This multi-site, randomized, double-blind study assesses the efficacy and safety of MDMA-assisted psychotherapy versus psychotherapy with placebo control in participants diagnosed with at least severe PTSD.

There will be separate open-label lead-in protocols following identical study procedures for new therapy teams to receive clinical supervision from the sponsor.

For each participant, the study will consist of:

- **Screening Period**: phone screen, informed consent, eligibility assessment, and enrollment of eligible participants
- **Preparatory Period with Enrollment Confirmation**: medication tapering, Preparatory Sessions and Baseline assessments leading to Enrollment Confirmation
- **Treatment Period**: three monthly Experimental Sessions and associated Integrative Sessions over ~12 weeks

- **Follow-up Period and Study Termination**: 4 weeks with no study visits, followed by Study Termination visit
- **Invitation to participate in Long-term Follow-up (LTFU) extension study**: 12 months after last Experimental Session

The treatment consists of a flexible dose of MDMA or placebo, followed by a supplemental half-dose unless contraindicated, administered with manualized psychotherapy in three blinded monthly Experimental Sessions. This ~12-week Treatment Period is preceded by three Preparatory Sessions. During the Treatment Period, each Experimental Session is followed by three Integrative Sessions of non-drug psychotherapy. Experimental Sessions are followed by an overnight stay; a sub-study (Appendix A) will assess feasibility of Experimental Sessions without an overnight stay. Upon unblinding after database lock, all participants who were assigned to placebo (regardless of CAPS-5 scores), completed the study, meet criteria, and are in good standing with the clinical site will be offered the opportunity to enroll in an open-label safety extension study of manualized MDMA-assisted psychotherapy.

Table 1: Study Design Overview

**Screening Period**<br>From Consent to Enrollment ~4 weeks (+/-2 weeks)			
**Study Visit**		**Visit Duration/ Visit Timing**	**Brief Description of Events**
Screening	Screening	Multiple visits over 7 to 28 days/ After phone screen/	At initial visit, obtain Informed Consent and assess all screening measures (including PCL-5, Lifetime C-SSRS), medical history, and pre-study medications. Contact outside providers and order medical records, physical exam, labs (including pregnancy and drug tests), ECG, and 1-minute rhythm strip. Once all results and records are obtained, review along with notes from all screening visits and measures. If eligible, send results of LEC-5 & SCID-5-SPQ to IR. Screening may take place over 7 to 28 days at multiple visits.
	Independent Rater Screening	1 hour/<br>2 to 9 days after initial eligibility established during Screening	After PCL-5 and initial eligibility are reviewed, an IR will conduct the Since Last Visit C-SSRS, SCID-5-PD, DDIS, and MINI via telemedicine. Results will be confirmed by clinical observation during the Preparatory Period, but the SCID-5-PD, DDIS, and MINI will not be repeated.
Enrollment	Enrollment	1.5 hours/<br>2 to 14 days after Independent Rater Screening	Prior to enrolling: review all screening measures, medical history, discussion with outside providers and sponsor, and any clarification phone calls with participant. Visit is 1.5 hours to review eligibility and medical tapering plan. If enrolled, begin taper, (5 half-lives plus 7 days for stabilization). Adverse Event (AE) collection begins.

**Preparatory Period with Enrollment Confirmation**<br>~6 weeks (+5/-5 weeks)			
**Study Visit**		**Visit Duration/ Visit Timing**	**Brief Description of Events**
Preparatory Period	Preparatory Session 1	1.5 hours/<br>0 to 12 days after Enrollment	90-minute Preparatory Session. Target visit timing on tapering needs. If needed, schedule calls between Visits if indicated for tapering, safety, or further questions about medical history.
	Preparatory Session 2	1.5 hours/<br>2 to 21 days after Prep Session 1	90-minute Preparatory Session/ongoing assessment. If tapering is complete or not needed, check eligibility and schedule upcoming visits. If tapering is ongoing, schedule post taper call for ongoing assessment.
	Phone Call End Taper	1 hour/<br>0 to 7 days after taper and stabilization end; up to 56 days after Enrollment; prior to Prep 3	If needed, confirm medication taper and stabilization is complete and participant is eligible for Baseline CAPS-5. Schedule Baseline CAPS-5 and Prep Session.

Baseline & Enrollment Confirmation	Baseline CAPS-5	1.5 hours/<br>Post Prep Session 2 & Medication Taper; before Prep Session 3	CAPS-5, SDS, and DSP-I completed by an IR via telemedicine after taper is complete. CAPS scores sent ASAP to the therapy team/PI.<br><br>Due to possibilities of negative side effects of withdrawal from psychiatric medications, the Baseline CAPS-5 should be scheduled as close to the end of tapering as clinically appropriate so that participants who do not meet eligibility criteria can resume their previously prescribed medications as quickly as required for symptom management and participants who do meet criteria can be enrolled and treated as quickly as possible.
	Preparatory Session 3 & Enrollment Confirmation	3 hours (90-minute measures, 90-minute therapy)/<br>3 to 6 days after Baseline CAPS-5	Complete Baseline self-report measures. Complete 3-hour Preparatory Session (~90-minute measures, 90-minute therapy) and schedule Exp. Session 1. If enrollment is not confirmed, do not randomize; complete Study Termination.

**Treatment Period**<br>From Randomization to Integrative Session 3.3 12 weeks (-3/+4)			
**Study Visit**		**Visit Duration/ Visit Timing**	**Brief Description of Events**
Treatment 1	Randomization	.5 hours/<br>Within 2 week of Baseline CAPS-5/<br>24 to 48 hours before Exp. Session 1	Complete after enrollment and scheduling Exp. Session 1. Enter demographics in Medrio for use in randomization. The participant does not need to be present for this.
Treatment 1	Experimental Session 1	8 hours + overnight/<br>Within 2 weeks of Baseline CAPS-5	8 hours with overnight stay. Dose is 80 mg with supplemental half-dose of 40 mg unless contraindicated.
Treatment 1	Integrative Session 1.1	1.5 hours/<br>Morning after Experimental Session 1	90-minute Integrative Session, followed by phone check-ins that includes Since Last Visit C-SSRS assessment on Days 2, 4, 6, 7, 8, 10, 12, 14 post Experimental Session.
Treatment 1	Integrative Session 1.2	1.5 hours/<br>3 to 14 days after Experimental Session 1 at least 2 days after Integrative Session 1.1	Between 3 and 14 days after Experimental Session, a 90-minute Integrative Session is completed.
Treatment 1	Integrative Session 1.3	1.5 hours/<br>20 to 34 days after Experimental Session 1; at least 2 days after Integrative Session 1.2; 1 to 7 days before Experimental Session 2	90-minute Integrative Session. Measures include LEC-5 and C-SSRS.
Treatment 2	Experimental Session 2	8 hours + overnight/<br>21 to 35 days after Experimental Session 1	The second Experimental Session lasts 8 hours with an overnight stay. Dose is 80 or 120 mg plus supplemental half-dose unless contraindicated.
Treatment 2	Integrative Session 2.1	1.5 hours/<br>Morning after Experimental Session 2	90-minute Integrative Session. Followed by phone check-ins that includes Since Last Visit C-SSRS assessment on Days 2, 4, 6, 7, 8, 10, 12, 14 post Experimental Session.
Treatment 2	Integrative Session 2.2	1.5 hours/<br>3 to 14 days after Experimental Session 2; at least 2 days after Integrative Session 2.1	90-minute Integrative Session.
Treatment 2	Integrative Session 2.3	1.5 hours/<br>20 to 34 days after Experimental Session 2; at least 2 days after Integrative Session 2.2 1 to 7 days before Experimental Session 3	90-minute Integrative Session . Measures include LEC-5 and C-SSRS.

Study Visit/ Visit #		Visit Duration/ Visit Timing	Brief Description of Events
Treatment 3	Experimental Session 3	8 hours + overnight/ 21 to 35 days after Experimental Session 2	The third Experimental Session lasts 8 hours with an overnight stay. Dose is 80 or 120 mg plus supplemental half-dose unless contraindicated.
	Integrative Session 3.1	1.5 hours/ Morning after Experimental Session 3	90-minute Integrative Session. Followed by phone check-ins that includes Since Last Visit C-SSRS assessment on Days 2, 4, 6, 7, 8, 10, 12, 14 post Experimental Session.
	Integrative Session 3.2	1.5 hours/ 3 to 14 days after Experimental Session 3; at least 2 days after Integrative Session 3.1	90-minute Integrative Session
	Integrative Session 3.3	1.5 hours/ 21 to 35 days after Experimental Session 3	90-minute Integrative Session. Measures include LEC-5 and C-SSRS.

**Follow-up Period and Study Termination**<br>From Integrative Session 3.3 \ until Study Termination: 4 weeks (±2). After Integrative Session 3.3			
**Study Visit/ Visit #**		**Visit Duration/ Visit Timing**	**Brief Description of Events**
Study Termination	Study Termination	2 hours; 1 to 9 days after Integrative Session 3.3	Occurs 105 to 147 days post Baseline. Complete self-reported and safety measures; create an exit plan for participant. Invite participant to the extension study for LTFU. Let participant who completed the protocol know how they will be informed of unblinding and at that time, if in the placebo group, regardless of CAPS-5 scores, they will be offered the opportunity to enroll at no cost in an open-label safety extension study.

Qualified, blinded IRs selected based on availability from the IR Pool will perform the CAPS-5 assessments.

5.2 Planned Duration of Study

Full screening may take 28 days after completion of phone screening. The Preparatory Period begins at enrollment and can be as brief as 7 days, but depending on medication tapering could be as long as 56 days to ensure an appropriate medication washout of at least five half-lives of pre-study psychiatric medications and active metabolites, and at least 7 days for stabilization.

Enrollment Confirmation takes place at the completion of the Preparatory Period, at which time the Treatment period will commence. The approximately 12-week Treatment Period will consist of three Experimental Sessions 3 to 5 weeks apart with associated non-drug Integrative Sessions and CAPS-5 assessments.

After the final Integrative Session 3.3 participants will enter follow-up with no planned study visits for approximately 4 weeks, after which point the Study Termination visit will take place.

The minimum time that a participant who completes all study visits from Screening to Study Termination will be in the clinical trial is 19 weeks, and the maximum is 38 weeks. The average participant is expected to complete the study in 27 weeks. Any delays between visits outside of the protocol-defined windows may result in a corresponding extension of study duration and should be documented as a deviation as appropriate.

All participants who complete the study will be asked to participate in a LTFU extension study with one visit 12 months after the last Experimental Session. Upon unblinding after database lock, all participants who were assigned to placebo (regardless of CAPS-5 scores), completed the study, meet criteria, and are in good standing with the clinical site will be offered the opportunity to enroll in an open-label safety study of manualized MDMA-assisted psychotherapy. This open-label safety study will take place at least 18 months after the first participant is enrolled in MAPP1 and will last 19 to 38 weeks, and will follow a design similar to this protocol.

5.2.1 Interruptions and Accommodations Due to COVID-19 Pandemic or Any Other Unforeseen Emergency at Clinic Locations

This clinical trial has been interrupted by the Coronavirus Disease 2019 (COVID-19) global pandemic. Accommodations may be required for study continuation and participant and study site staff safety due to this emergency or any other unforeseen emergency in the future. The following accommodations in the protocol will be allowed, captured and noted in the Clinical Study Report as COVID-19 deviations:

- Integrative Sessions may be conducted by telemedicine
- Delaying the start of medication tapering after enrollment and the subsequent Treatment Period per Section 12.1 Tapering Instructions
- Delaying Experimental Sessions and associated Integrative Sessions
- Delaying Independent Rater assessments for participants who cannot complete them remotely off-site
- Use of prohibited medications and/or cannabis or initiation of new psychotherapy for participants with significant study delays, which will be reviewed by the study team before each Experimental Session and re-tapered prior to resuming treatment per Section 12.0 Concomitant Medications.

For any participant with COVID-19 related illness, continued trial participation after full recovery of the disease may be appropriate after discussion between the site physicians and Medical Monitors on a case-by-case basis.

5.3 Discontinuation and Completion Criteria

5.3.1 Complete or Evaluable Participants

Participants who are considered complete or evaluable will be defined as follows:

- A participant is considered ‘Evaluable’ and eligible for the mITT analysis if they have completed at least one Experimental Session and one CAPS-5 assessment beyond Baseline.
- A participant is considered ‘Evaluable and Completed Per Protocol’ if they have completed all Experimental Sessions and CAPS-5 assessments as planned. These participants will be included in the mITT analysis set and the Per Protocol analysis set.

- A participant is considered 'Evaluable and Early Termination' if they have completed at least one Experimental Session and one CAPS-5 assessment beyond Baseline but terminated early. These participants will be included in the mITT analysis.
- A participant is considered to be in good standing with the clinical site if, in the opinion of the investigator and/or therapy team, the participant was compliant with protocol requirements, even if they were unable to complete all study visits.

5.3.2 Screen Failures

'Screen Failures' are defined as participants who pass phone screening but are deemed ineligible before successfully enrolling in the study at Enrollment. Screen failures may fail to meet all Inclusion Criteria and may meet one or more Exclusion Criteria or withdraw consent prior to Enrollment. All potential participants who begin Screening will be tracked on a Screening Log, and reasons for Screen Failure will be recorded. Screen Failures are not considered evaluable.

Screen Failures may be identified through review of medical history, assessments, measures, laboratory results, or conversations with the participant. Medical assessments may be repeated for confirmation. At any time during Screening, if a potential participant is deemed to be ineligible, classify as a Screen Failure, notify the potential participant that they are unfortunately not eligible for the study, and do not schedule additional Screening assessments. Participants who fail Screening may be rescreened at a later date if deemed appropriate by the investigator but should sign a new copy of the Informed Consent Form (ICF). Screen Failures may request a referral to an outside therapist if needed. Screen Failures that were scheduled for an IR assessment will be entered into the Electronic Data Capture (EDC) system.

5.3.3 Pre-Randomization Early Terminations

'Pre-randomization Early Terminations' are defined as participants who were deemed eligible and enrolled in the study, but are deemed ineligible prior to the first Experimental Session and do not have enrollment confirmed at Enrollment Confirmation. These participants may fail to meet all Inclusion Criteria and may meet one or more Exclusion Criteria or withdraw consent prior to Enrollment Confirmation or the site may withdraw the participant for reasons described in Section 5.3.4 Early Termination from the Study). Participants who fail Enrollment Confirmation may not be re-enrolled into this study at a later date. All enrolled participants, even those failing Enrollment Confirmation, will be maintained in the EDC system. Pre-randomization Early Terminations are not considered evaluable.

Pre-randomization Early Terminations may be identified through review of medical history, assessments, measures, laboratory results, or conversations with the participant. At any time during the Preparatory Period, if a potential participant is deemed to be ineligible, classify as a Pre-randomization Early Termination, notify the potential participant that they are unfortunately not eligible for the study, and do not schedule additional assessments. Do not randomize the participant. Pre-randomization Early Terminations will be provided an Exit Plan as described in Section 8.4.3.2 Exit Plan.

5.3.4 Early Termination from the Study

Participants who are removed from the study after they are randomized and receive IMP but do not complete the study may fall into one of these categories: Post-randomization Early Termination or Dropout. If the participant has received IMP in at least one Experimental Session and completed one CAPS-5 assessment beyond Baseline, they will be considered evaluable. All

participants who receive IMP in at least one Experimental Session will be included in all safety analyses.

Participants can withdraw from treatment or withdraw consent at any time for any reason without judgment. The site team can withdraw a participant if, in their clinical judgment, it is in the best interest of the participant or if the participant cannot comply with elements of the protocol that are critical for safety or for the scientific integrity of the study. If the site team makes the decision to terminate the participant from treatment or the study, they will explain the reason for withdrawal and document in the participant's source records and Electronic Case Report Form (eCRF). If a participant develops any Exclusion Criteria that, in the opinion of the Medical Monitor or Site, affects the safety of the participant, including psychiatric diagnosis, medical diagnosis, pregnancy, or requiring use of prohibited medications, the participant will discontinue treatment in Experimental Sessions but remain in the study for the associated Integrative Sessions. Participants whose Experimental Sessions are delayed by the COVID-19 pandemic may start any of these medications during the delay, as clinically indicated. When study visits resume, they will be given the option to resume Experimental Sessions after tapering off of these medications per Section 12.1 Tapering Instructions. Any time a participant terminates from the study early, the site team will attempt to obtain information about AE outcomes if appropriate, as determined by the site physician and Medical Monitor. The site team will provide the participant with an Exit Plan as described in Section 8.4.3.2 Exit Plan.

Data will be collected from participants who terminate early as follows:

- **Post-randomization Early Termination**: Participants who discontinue study treatment but continue to participate in Independent Rater primary and secondary outcome assessments. Data collection by IRs will continue on the same schedule as planned through Study Termination visit procedures.
- **Dropout**: If a participant decides to withdraw consent completely, they will terminate without further follow-up. If the participant agrees, they will complete a final CAPS-5 assessment. These participants are defined as dropouts who withdraw consent due to any reason after receiving at least one dose of IMP and no longer participate in the study (i.e., no further contact with investigators or site staff). Data collected on study participants up to the time of withdrawal of consent will remain in the trial database in order to maintain scientific validity. Removal of data from the database would undermine the scientific and ethical integrity of the research.

5.3.5 Lost to Follow-up

A participant will be considered lost to follow-up if they fail to attend scheduled visits and are unable to be contacted by the site staff. If the participant has completed at least one Experimental Session and one CAPS-5 assessment beyond Baseline, they will be considered evaluable. All participants with at least one Experimental Session will be included in the safety analysis.

If a participant does not attend a scheduled visit, the site must attempt to contact the participant to reschedule the visit as soon as possible and emphasize the importance of complying with the protocol specified visit schedule. The staff should determine if the participant is willing to comply with future visits.

If a participant does not respond to this initial contact, the site staff must make multiple efforts to contact the study participant and document each attempt in the source record. At least three attempts should be made via telephone, over the course of approximately 7 days, with calls at different times of day. If telephone contact fails, an email should be sent if such contact

information was provided. The emergency contact the participant provided should be contacted and asked to attempt contact with the participant. Lastly, a certified letter (or equivalent) should be sent to their last known mailing address. If the participant fails to respond to all of these contacts, they will be considered to have withdrawn from the study and are lost to follow-up.

5.4 End of Study Definition and Premature Discontinuation

The end of the study is defined as the date of the last visit of the last participant in the study or last scheduled procedure for the last participant in the trial globally.

The sponsor has the right to discontinue this study at any time. If the trial is prematurely terminated, the investigator is to promptly inform participants and will ensure they receive appropriate therapy, follow-up, and Exit Plan. If the study is prematurely discontinued, all procedures and requirements pertaining to retention and storage of documents will be observed. All other study materials will be returned to the sponsor and will be treated in accordance with federal and state regulations.

5.5 Rationale of Dose Selection

Similar MDMA doses to those proposed in this study have been safely used in previous Phase 2 studies sponsored by MAPS. Phase 2 studies indicate that 75, 100 and 125 mg MDMA initial doses with the supplemental dose are active and effective in two to three Experimental Sessions. MDMA doses with an optimal risk-benefit ratio range from 75 mg (Cohen's d Independent Groups Pre-test Post-test [dIGPP]=2.73, N=7) to 125 mg (Cohen's dIGPP=0.77, N=58) initial dose of MDMA with a 2-session treatment package. In Phase 2 studies, the sponsor observed a -36.4 point mean change in CAPS-4 scores among active dose participants receiving two Experimental Sessions (N=72) compared to a -44.2 point mean change after three Experimental Sessions (N=51). Although uncontrolled, the additional 7.8 point mean reduction observed after three Experimental Sessions compared to two, along with the observed favorable safety profile formed the basis for selection of a 3-session treatment package. A flexible dosing regimen has been explored previously in Phase 2 studies, where participants who received doses of 0 mg to 75 mg MDMA in the blinded portion of the study crossed over to receive open-label 100 mg MDMA in the first Experimental Session with an option to increase to 125 mg MDMA in the second and third Experimental Sessions. In the opinion of the participants and therapy teams administering this treatment in Phase 2 studies, this flexible dosing regimen and three Experimental Sessions produced an optimal treatment response. Larger doses have been administered safely in MP2 (150 mg and 75 mg supplemental) and in Phase 1 studies (150 mg and 160 mg). The results of these Phase 2 studies led to the selection of 80 mg and 120 mg MDMA as the initial active doses to be compared to inactive placebo in Phase 3 trials.

This blinded study will compare the effects of a flexible dose of MDMA or placebo administered in three Experimental Sessions. Initial doses per Experimental Session range from 80 mg to 120 mg of MDMA compounded with inactive excipients or indistinguishable weight placebos comprised entirely of inactive excipients, followed 1.5 to 2 hours later by a supplemental half-dose (40 mg or 60 mg, or placebo). A flexible dosing regimen was chosen to mimic proposed clinical practice and better adapt to risk-benefit considerations. The initial active doses of 80 mg and 120 mg are expected to produce all commonly reported effects of MDMA. The supplemental half-dose will prolong subjective effects of MDMA without producing physiological effects much greater than peak effects occurring after the initial dose, and will be administered unless contraindicated. Total amounts of MDMA to be administered per Experimental Session range from 80 mg to 180 mg.

An inactive placebo has been selected as the control to allow differentiation of any additional MDMA effect from psychotherapy alone. This pivotal study will generate unequivocal assessment of safety data and demonstration of efficacy of MDMA-assisted psychotherapy compared to psychotherapy plus placebo.

Table 2: Dose Regimen of MDMA or Placebo

Experimental Session	Initial Dose	Supplemental Dose*	Min-Max Cumulative Dose
1	80 mg	40 mg	80 mg to 120 mg
2	80 or 120* mg	40 or 60 mg	80 mg to 180 mg
3	80 or 120* mg	40 or 60 mg	80 mg to 180 mg
		**Total Cumulative Dose**	240 mg to 480 mg

* Unless contraindicated

In the first Experimental Session, the initial dose will be 80 mg MDMA or placebo. In the second and third Experimental Sessions, the initial dose may be increased to 120 mg MDMA or placebo unless contraindicated in the opinion of the site team. The choice of whether to keep the dose the same or change it from the first Experimental Session will be made by the site team based on observed response, tolerability to the previously administered dose, and discussion with the participant. In each Experimental Session, 1.5 to 2 hours after the initial dose is given, the participant will be administered a supplemental half-dose unless contraindicated. Participants will not know if they have been assigned MDMA or placebo but can indicate if they want the dose to change or remain the same.

6.0 Psychotherapy

6.1 Description of Therapeutic Method

The largely non-directive therapeutic method of MDMA-assisted psychotherapy is described in detail in the Treatment Manual. All therapy teams will be extensively trained in a multi-week training program prior to the study to ensure all participants are treated in a similar manner. The non-directive approach pertains to inviting inquiry and providing suggestion rather than directing the participant in the therapeutic approach. This requires active or engaged listening and responding, as well as facilitation of therapeutic action by providing support for approaching difficult material in a manner that does not interfere with the participant’s spontaneous experience.

6.2 Therapy Team Qualifications

Therapy teams will be trained by the sponsor. Sites must ensure that the minimum requirements below are met:

- One person licensed to manage and administer controlled substances for each site
- A physician to assess participant safety at Screening
- One or more two-person therapy teams, male/female preferred
- One person per therapy team is required to be licensed to provide psychotherapy according to state and local requirements
- If one person on the therapy team is unlicensed, they will work under the direct supervision of the licensed team member

6.3 Training

The sponsor's Therapy Training Program is designed to teach competency in applying the essential elements of this method of MDMA-assisted psychotherapy. Therapy team members will receive specific training in the MDMA-assisted psychotherapy method, protocol, and latest version of the IB. Training in the psychotherapy method consists of reading the Treatment Manual, completing an online training module, and participating in an in-person training program that includes watching and discussing videos of Experimental Sessions. The final part of training includes supervision from the training team during a Phase 2 open-label lead-in protocol. The required elements of the therapy are defined in the Treatment Manual, and teams will be trained on visit-specific sets of adherence criteria. In addition to this specific training, it is required that participating therapy team members have the proper background, education, and experience.

6.4 Adherence to Therapeutic Method

Psychotherapy sessions, including Experimental Sessions, may be recorded, with recordings preserved for research and training purposes. Adherence criteria and competence ratings will be conducted by qualified, trained, and blinded Adherence Raters who will analyze video data from specific and randomly selected Preparatory Sessions, Experimental Sessions, and Integrative Sessions. The elements included in adherence criteria are specific to each type of session and are defined in the Treatment Manual. These ratings will be collected, at minimum, for each therapy team in the study. Ratings will be used to provide feedback to new therapy teams, to further characterize the manualized therapy, and for future exploratory research.

7.0 Measures and Reliability

The following eligibility, outcome, exploratory, and safety measures will be used in the study, in accordance with the study protocol.

Table 3: Protocol Objectives and Assessment Tools

Objectives	Measure	Measure Type	Administration
Eligibility			
Assess psychiatric disorders	MINI	Eligibility	Telemedicine (IR)
Assess personality disorders	SCID-5-PD with SCID-5-SPQ	Eligibility	Telemedicine (IR)/ self-report measure at Site
Confirm PTSD diagnosis and severity	PCL-5 with LEC-5	Eligibility	Site
Identify dissociative disorders	DDIS	Eligibility	Site
Primary			
Assess changes in PTSD symptom severity from compared between groups	CAPS-5	Outcome	Telemedicine (IR)
Secondary			
Assess changes in clinician-rated functional impairment from compared between groups	SDS	Outcome	Telemedicine (IR)

**Objectives**	**Measure**	**Measure Type**	**Administration**
Safety			
Compare relative incidence of positive or serious ideation and suicidal behavior between groups	C-SSRS	Safety	Site
Exploratory			
Characterization of CAPS-5 Total Severity score to explore onset of treatment efficacy with a descriptive time course plot	CAPS-5	Outcome	Telemedicine
Explore changes in PTSD symptom clusters of re-experiencing, avoidance, negative alterations in cognition and mood, and hyperarousal as measured by changes in CAPS-5 subscale scores	CAPS-5	Outcome	Telemedicine
Assess changes in severity of dissociative symptoms associated with PTSD compared between groups	DSP-I	Outcome	Telemedicine (IR)
Explore correlation of dissociative symptoms associated with PTSD with the CAPS-5 Total Severity analyses	DSP-I<br>CAPS-5	Outcome	Telemedicine (IR)
Explore the effect of presence of secondary traumatic stressors during the assessment period as a covariate on the CAPS-5 Total Severity analyses	LEC-5<br>CAPS-5	Outcome	Site or Telemedicine
Explore the effect of adverse childhood experiences on PTSD treatment outcomes as a covariate on the CAPS-5 Total Severity analyses	ACE<br>CAPS-5	Outcome	Site or Telemedicine
Assess changes in depression symptoms compared between groups	BDI-II	Outcome	Site or Telemedicine
Assess changes in chronic pain from compared between groups	CPGS	Outcome	Site or Telemedicine
Assess changes in quality of life compared between groups	EQ-5D-5L	Outcome	Site or Telemedicine
Assess changes in functioning in relation to self and others from compared between groups	IASC	Outcome	Site or Telemedicine
Assess changes in self-reported psychosocial functioning compared between groups	IPF	Outcome	Site or Telemedicine
Assess changes in self-compassion compared between groups	SCS	Outcome	Site or Telemedicine
Assess changes in alexithymia compared between groups	TAS-20	Outcome	Site or Telemedicine
Assess changes in alcohol use compared between groups	AUDIT	Healthcare cost	Site or Telemedicine
Assess changes in drug use compared between groups	DUDIT	Healthcare cost	Site or Telemedicine
Assess changes in nicotine use compared between groups	SRNU	Healthcare cost	Site or Telemedicine
Assess changes in disordered eating compared between groups	EAT-26	Healthcare cost	Site or Telemedicine
Assess changes in workplace productivity compared between groups	HPQSF	Healthcare cost	Site or Telemedicine
Assess facility-based healthcare utilization at Screening	UFEC	Healthcare cost	Site or Telemedicine

7.1 Primary Outcome Measure and Reliability

7.1.1 CAPS-5 (Clinician-Administered PTSD Scale for DSM-5)

The last month CAPS-5 is a semi-structured interview that assesses index history of DSM-5-defined traumatic event exposure [62], including the most distressing event, time since exposure, to produce a diagnostic score (presence vs. absence) and a PTSD Total Severity score [62]. The CAPS-5 rates intrusion symptoms (intrusive thoughts or memories), avoidance, cognitive and mood symptoms, arousal and reactivity symptoms, duration and degree of distress and dissociation. The CAPS-5 will be administered by a blinded IR via telemedicine. Interviews will be conducted by the centralized remote IR pool to enhance quality control by reducing site-level variation in interview fidelity and quality. The IRs will be trained and supervised by a research reliable trainer and will be supervised by qualified personnel. Per the CAPS-5 Training Manual for the IR Pool, IRs will ensure that every single item-level score is collected in every CAPS-5 interview. The CAPS-5 is administered by the IR in a neutral, non-leading manner to minimize the chance for bias. Additionally, the IRs are trained to carefully assess trauma-relatedness of each symptom according to standard CAPS-5 procedures. Doing so ensures that all symptoms contributing to the CAPS-5 PTSD diagnostic status and total severity score are either temporally or functionally related to the index trauma rather than attributable to non-trauma current life stressors or current world events such as the COVID-19 pandemic. Avoiding a biased administration can be achieved by adhering to administration guidelines verbatim and only deviating from the script to clarify, re-direct, or query further if behavioral examples are needed to determine the appropriate symptom intensity rating. Avoiding building therapeutic/clinical rapport beyond the basic level of rapport needed to conduct the interview in the research setting also minimizes the chance for bias. Remote assessment assures that the rater who is collecting the Primary Outcome will not witness Experimental Sessions and the acute effects of IMP, which strengthens the study blind. Interviews may be recorded in as many instances as necessary to establish reliability of a random selection of interviews for accuracy. After the initial screening visit, the IRs will be blinded to visit number, number of treatments received, treatment assignment, and any study data for the participant. IR visits will be assigned based on availability.

7.2 Secondary Measure

7.2.1 SDS (Sheehan Disability Scale)

The SDS is a clinician-rated assessment of functional impairment [63]. The items indicate degree of impairment in the domains of work/school, social life, and home life, with response options based on an eleven-point scale (0=not at all to 10=extremely), and five verbal tags (not at all, mildly, moderately, markedly, extremely). The SDS takes 1 to 2 minutes to complete. The measure can be scored for each of the three domains. The SDS has high internal consistency and accurately identified 80% of a sample of primary care patients with mental disorders [64].

7.3 Safety Measures

7.3.1 C-SSRS (Columbia Suicide Severity Rating Scale)

The C-SSRS is a clinician-administered measure of suicidal behavior devised to detect potential suicidal thoughts or behaviors during a clinical trial [65]. It consists of a Lifetime version and a Since Last Visit version that assess suicidal ideation, ideation intensity, and behavior. The C-SSRS consists of a series of questions and can be administered during a face-to-face interview or over the telephone. The Lifetime version will only be administered at the initial Screening visit. All subsequent administrations will utilize the Since Last Visit version. Participants who are

discontinuing medications to participate in the study will complete the C-SSRS before and after medication washout. The C-SSRS Intensity scale for Lifetime obtained a Cronbach's alpha of 0.93 and 0.94 for the Since Last Visit form, and Last Visit C-SSRS severity scores were positively correlated with the BDI "suicide thoughts" item [66].

7.4 Screening Measures and Reliability

7.4.1 MINI (Mini-International Neuropsychiatric Interview)

This version of the MINI (7.0.2), a structured interview that was first developed in 1998 to be compatible with DSM and International Classification of Disease (ICD) criteria for psychiatric illnesses [67], is now compatible with DSM-5 and will be administered by a member of the Independent Rater Pool to screen for psychiatric conditions per DSM-5. Each module of the MINI consists of two or three questions where the answer is either "Yes" or "No," and decision-tree logic is used to determine whether to ask additional questions [68]. The MINI takes between 15 and 20 minutes to perform and addresses major psychiatric disorders. MINI items were highly reliable (interrater reliability between kappa of 0.8 and 0.99; test-retest reliability between 0.6 and 0.9 for all scales save "current mania), and diagnosis via MINI was comparable to that made with the Composite Diagnostic Interview and the SCID [68, 69] Testing on nonpsychiatric samples did not create false positives [67].

7.4.2 SCID-5-PD (Structured Clinical Interview for DSM-5 for Personality Disorders)

The SCID-5-PD will be administered by a blinded IR via telemedicine [70]. Prior to the SCID-5-PD clinical interview, participants will complete a brief self-report questionnaire called the SCID-5 Self-report Personality Questionnaire (SCID-5-SPQ) as a self-report screening tool used to assess for personality disorders. Potential personality disorders that satisfy diagnostic thresholds will be further assessed via clinical interview during the SCID-5-PD. IRs will receive training on administering these measures from a research reliable trainer. Interviews may be recorded in as many instances as necessary to establish reliability of a random selection of interviews for accuracy.

7.4.3 LEC-5 (Life Events Checklist for DSM-5)

The LEC-5 is a 17-item self-report instrument designed to determine the presence of traumatic life events in the assessment and diagnosis of PTSD. It is a companion measure to the PCL-5 and will be used to assess PTSD. The participant indicates whether each event listed has occurred during their lifetime, permitting the possibility of marking multiple events [71].

7.4.4 PCL-5 (PTSD Checklist)

The PCL-5 is a 20-item self-report questionnaire in which respondents indicate the presence and severity of PTSD symptoms, derived from the symptoms of PTSD per DSM-5 [72]. Participants indicate how much distress they have experienced due to symptoms such as "Repeated, disturbing memories, thoughts, or images of a stressful experience from the past," "Trouble remembering important parts of a stressful experience from the past," and "Feeling irritable or having angry outbursts" on a five-point Likert-type scale (1=Not at all to 5=Extremely).

7.4.5 DDIS (Dissociative Disorders Interview Schedule for DSM-5)

Questions on the DDIS specifically addressing dissociative disorder symptoms (items 117 to 130) will be asked by an IR during Screening. These questions are part of an intensive interview that includes questions concerning somatic and psychiatric symptoms. The interview is intended to assess and potentially distinguish between dissociative disorders and other disorders and between Dissociative Identity Disorder (DID) and a dissociative disorder not otherwise specified [73]. Owing to overlap with items found between the DDIS and other screening measures.

7.5 Exploratory Measures

7.5.1 DSP-I (The Dissociative Subtype of PTSD Interview)

The DSP-I is a clinician-administered interview designed by an international team of PTSD researchers to detect and assess severity of the dissociative type of PTSD and recommended for use as an additional or complementary measure ("add-on") to the CAPS-5 [74]. Assessments of military veterans and civilians support the existence of a dissociative subtype of PTSD that is associated with PTSD severity and derealization and depersonalization [75-77]. The DSP-I takes approximately 5 to 15 minutes to complete. It consists of two parts, only Part 1 will be administered. Part 1 contains five items addressing depersonalization, four items addressing derealization, and a section that is administered if dissociative episodes are endorsed that assesses duration and perceived cause of episodes (seven items) and observer items (three items) addressing interviewee demeanor, including evidence of dissociation, such as forgetfulness or giving a statement that is bizarre within the context of the interview. If two or more items within this section are endorsed, this indicates the presence of other dissociative symptoms beyond depersonalization and derealization. The DSP-I was first developed in 2016 and revised in 2017.

7.5.2 ACE (Adverse Childhood Experience Questionnaire)

The ACE is a 10-item checklist measure assessing number and types of adverse childhood experiences, including neglect and emotional, physical, and sexual abuse. Respondents are asked if an experience happened "often" and if so, to write "1". The total score reflects the number of adverse childhood experiences. The measure was first used in the context of a study investigating the relationship between childhood adverse experiences and health outcomes in adulthood [78]. Number of frequent adverse childhood experiences is associated with adverse health outcomes in adulthood, including greater likelihood of heart disease, chronic pain, and poor work performance [79-82]. The scoring method has been used in archival research, finding an association between increased scores and health problems in several generations [81].

7.5.3 BDI-II (Beck Depression Inventory II)

The BDI-II is a revision of the BDI, a 21-item self-report measure [83, 84] that will serve as a measure of depression symptom severity [85]. The BDI-II has been validated, has high internal consistency and good test/re-test reliability, and is not overly sensitive to daily variations in mood. It takes 5 to 10 minutes to complete [85]. Score cutoffs indicate: 0 to 13 minimal depression, 14 to 19 mild depression, 20 to 28 moderate depression, and 29 to 63 severe depression. Initial and subsequent studies report that the BDI-II total score has a reliability coefficient of 0.90 to 0.91 which is related to other measures of depression symptoms [85, 86]. Higher scores indicate more severe depressive symptoms.

7.5.4 CPGS (Chronic Pain Grade Scale)

The CPGS is a seven-item measure of pain. Responses to six of the seven items are made on a 10-point Likert scale, and a response on the other item is the number of days in the past 3 to 6 months when pain prevented the respondent from carrying out everyday activities [87]. Responses to questions are used to attain a rating (grade) for pain from zero (no pain) to five (high disability, severely limiting). The instrument has three scale scores: pain severity, pain intensity, and pain-related disability. Estimated time to complete is 3 to 5 minutes. The CPGS is a validated scale with high internal consistency (Cronbach's alpha=0.90) and correlated with other instruments assessing pain [88].

7.5.5 EQ-5D-5L (EuroQol Five Dimensions-Five Levels Questionnaire)

The EQ-5D-5L is a two-part self-report questionnaire assessing health status. It consists of five dimensions; mobility, self-care, usual activities, pain-discomfort and anxiety-depression, and one visual analog scale (VAS). Responses are made on each dimension by checking one of five statements that best reflects their health on the day of measure completion, from the healthiest or fewest problems (e.g., "I have no trouble walking about") to the most trouble (e.g., "I am unable to walk about") [89, 90]. In the second part of the EQ-5D-5L, current degree of health ("your health today") is indicated by marking a 20 cm line marked form one to 100, with 100 considered "the best health you can imagine" and one "the worst health you can imagine." The EQ-5D-5L does not sum responses, but treats each response on a dimension as a scale score, and the VAS is the location of the mark in centimeters. The scale can permit comparison across groups on health profiles, and an index can be derived from matching the five dimension scores and the VAS response with nation-specific datasets and calculator software or statistical software syntax designed for the measure. The EQ-5D-5L began as part of the EuroQoL measure, published in 1990 [91]. The instrument has been validated in populations from eight countries. EQ-5D-5L index scores and VAS scores assessed in people with stroke varied with degree of recovery assessed via long-term observation. EQ-5D-5L index scores and VAS scores assessed in people with stroke varied with degree of recovery assessed via long-term observation [92]. The EQ-5D-5L takes about 3 minutes to complete.

7.5.6 IASC (Inventory of Altered Self Capacities)

The IASC is a 63-item self-report measure of difficulties with relationships, identity, and affect regulation [93]. The measure is completed by rating the frequency that an event or experience has occurred in the last 6 months or last month (1=Never to 5=Very often) on a 5-point Likert scale. The IASC consists of seven 9-item scales: interpersonal conflicts, idealization-disillusionment, abandonment concerns, identity impairment, susceptibility to influence, affect dysregulation, and tension reduction activities. Subscale scores are summed into raw scale scores. Level of symptomatology is assessed through use of t-scores based upon scale norms. IASC subscales range in reliability from Cronbach's alpha of 0.78 to 0.93. IASC scale scores were correlated with a measure of personality disorders and psychiatric symptoms, and affect dysregulation scale scores were associated with a measure of depression [93].

7.5.7 IPF (Inventory of Psychosocial Functioning)

The IPF will be administered as an exploratory measure at Baseline and 2-Month Follow-up. The IPF is a self-report instrument designed to assess functional impairment across a spectrum of domains [94]. The IPF is an 80-item measure that was developed for use among individuals with PTSD. It assesses current psychosocial functioning across seven domains: romantic relationships, family, work, friendships, parenting, education, and self-care. Responses are made on a six point

Likert scale, (1=Never to 6=Always). Domain scores can be computed for each subscale, which possess positive correlations with similar scales or subscales on other measures. The IPF has excellent psychometric properties, with Cronbach's alpha for the total scale computed at 0.93 and good internal consistency for subscales (ranging from 0.80 to 0.90) [95].

7.5.8 SCS (Self-Compassion Scale)

The SCS is a 26-item self-report measure of self-compassion, or responding to one's own failure, suffering or inadequacies with kindness and compassion and recognizing one's own flaws and suffering as part of common human experience [96]. Respondents complete the SCS by indicating how typical they feel on each item on a 5-point Likert scale (1=Almost never and 5=Almost always). It is estimated to take between 4 to 8 minutes to complete. The scale has six sub-scales: Self-Kindness, Self-Judgment, Common Humanity, Isolation, Mindfulness, and Over-Identified. The mean of subscale scores serves as a total score. Analysis of SCS response indicated that subscales are all related to a higher order factor of self-compassion, and the measure has high test-test reliability at a level of 0.93. Neff et al. reported an inverse relationship between SCS total scores and scores on measures of depression and anxiety. Self-compassion and global self-esteem are both related to positive mood and optimism, but self-compassion may be more strongly associated with stable mood and less associated with self-rumination and anger [97].

7.5.9 TAS-20 (Toronto Alexithymia Scale)

The TAS-20 is a 20-item measure of self-reported difficulties with recognizing and verbalizing emotions [98, 99]. Responses are made on a 5-point Likert scale (1=Strongly disagree to 5=Strongly agree). Estimated time of measurement is 5 to 8 minutes. The scale is comprised of three subscales: Difficulty Describing Feelings, Difficulty Identifying Feelings, and Externally-Oriented Thinking, with all scales summed to create a total score reflecting presence and degree of alexithymia. The TAS-20 is an established measure and can be used diagnostically with a score of 61 or higher indicative of alexithymia. The TAS-20 is reliable and has good test-retest reliability (Cronbach's alpha of 0.81, test-retest of 0.77).

7.5.10 AUDIT (Alcohol Use Disorders Identification Test)

The AUDIT is a ten-item self-report test. Respondents answer on a 5-point scale (0=Never or none, 4=Daily or greatest number) [100]. The ninth item addresses occurrence of injury of self or other as a result of drinking and the tenth addresses others' concerns about the respondent's drinking, with only three responses provided (0=No, 2=Yes, but not during the last year, 3=Yes, during the last year). The measure can readily detect alcohol abuse disorders in a wide array of individuals [101].

7.5.11 DUDIT (Drug Use Disorders Identification Test)

The DUDIT is an 11-item measure designed to assess presence of substance use disorders [102]. Responses to items are made on a 5-point scale with exact responses varying across questions. When present, use can be described in monthly or less than monthly versus four times a week or daily. A list of substances is provided at the end of the measure. The DUDIT is reliable, with a Cronbach's alpha of 0.80. When compared with an interview based on ICD 10, the DUDIT had a sensitivity to detecting substance use disorders of 90% and a specificity of 80% [102]. The English translation was developed from a Swedish-language original. Estimated time to complete is 2 to 4 minutes.

7.5.12 SRNU (Self-reported Nicotine Use)

The SRNU is a sponsor-developed measure that will assess participant's use of nicotine, including approximate frequency of use in the last month and attitudes towards quitting. The measure will take less than 3 minutes to complete.

7.5.13 EAT-26 (Eating Attitudes Test)

The EAT-26 is a 26-item self-report measure that assesses attitudes about eating and food and is used to assess presence of eating disorders. Responses are made on a six-point scale (1=Always to 6=Never), and gathers information on gender, age, height, and weight. The EAT-26 produces a total score and can be used to generate a "referral score." The 27th item addresses the occurrence and frequency of specific eating behaviors, such as binge eating. Estimated time to complete is 4 to 8 minutes. Items on the EAT-26 have high reliability coefficients (Cronbach alpha of 0.83 to 0.90) and has concurrent validity [103].

7.5.14 HPQSF (Health and Work Performance Absenteeism and Presenteeism Short Form)

The HPQSF is a short form of a larger measure of health and work performance that has selected items referring to absenteeism and work performance [104]. The larger measure was created by the WHO as part of the Global Burden of Disease initiative. It consists of eight questions selected from the larger Health and Work Performance Questionnaire, with one question containing five additional items. Items include questions concerning hours worked during an average week, number of whole and partial days missed during a 4-week period, and items that rate average coworker and self-work performance on a ten-point Likert scale (1=Worst performance to 10=Top performance). Hours spent in work over a 4-week period and over the last 7 days can be used to estimate absenteeism, and the HPQSF can also score presenteeism, a measure of actual performance in relation to possible performance. Self-reports on measure appear to match employer records of presence or absence [105], and the HPQSF appears to be reliable between one time point and another (reliability of 0.52) and is sensitive to change [104].

7.5.15 UFEC (Utilization of Facility-based and Emergent Care)

The UFEC is a sponsor-developed measure assessing participant health events, including hospitalization and use of healthcare facilities, including in-patient hospitalization, rehabilitation facilities and other health care facilities for a set period prior to study entry.

8.0 Study Procedures

All assessments must be performed by qualified study staff delegated these duties on the Site Responsibilities Log. The Clinical Research Associate (CRA) should be notified of any delays or deviations to study procedures and Medical Monitor consulted if necessary. If there are delays of more than 7 days between visits or contact, the site should assess the need for additional telephone contact with the participant to ensure safety.

8.1 Screening Period

8.1.1 Screening

Prospective participants will be pre-screened by telephone according to an Institutional Review Board (IRB)-approved script to ascertain if they meet basic eligibility criteria. All individuals who are pre-screened should be assigned a Screening Number and recorded on the Screening Log. Data from potential participants who do not pass telephone screening will not be entered in the eCRF but reason of ineligibility will be documented on the Screening Log. At any time during Screening, if a potential participant is deemed ineligible, they will be classified as a Screen Failure, notified that they are not eligible for the study, and not be scheduled for any additional Screening assessments.

If deemed potentially eligible, the potential participant will receive a copy of the ICF for review and invited to the site for in-person screening. Relevant medical and psychiatric records are required for the site physician to obtain a well-characterized medical history and assess eligibility. The physician may need to contact the prescribing physician to discuss the tapering of medications (see Section 12.0 Concomitant Medications).

Site staff (preferably the therapy team who would be treating this potential participant) will explain and obtain written informed consent using the IRB-approved ICF. Written consent must be obtained prior to performing any tests or evaluations for the study. Discussion about the ICF may take place over a telemedicine visit or at the first in-person visit. If a participant fails Screening and is rescreened at a later date, a new copy of the ICF should be signed.

Screening will take place over multiple visits and will be completed in-person, via telemedicine, or over the telephone. All procedures must be completed but there can be some flexibility in timing and order of individual assessments within the Initial Eligibility and Medical Assessments categories below:

- Initial Eligibility, including measures, in-person discussions, and review of medical records
- Medical Assessments, including labs, electrocardiogram (ECG), and physical exam
- The site staff will schedule the IR assessment and send IR the results of initial measures

The sponsor recommends the following order of assessments:

Initial Eligibility
Qualified site staff will:

- Review medical and psychiatric history with the participant via interview and review of provided records. If no records were provided or those provided are not sufficient, request additional records. Collect data on previous hospitalizations and healthcare utilization.
- Support the participant during completion of the LEC-5 with PCL-5.Administer the Lifetime C-SSRS to assess history of suicidal behavior and ideation.
- Review past and current medications and adherence to prescriptions.
- Assess childbearing potential and discuss requirement for commitment to adequate birth control for the duration of the study. Perform urine pregnancy test for participants who are of childbearing potential.
- Perform a urine drug test.
- Direct participant to complete self-reported Screening measures.

- Review results of all measures and discussions against eligibility criteria to assess initial eligibility. If deemed initially eligible, potential participant will be provided with instructions (and appointments, if applicable) for a physical exam, laboratory assessments, an electrocardiogram (ECG), and 1-minute rhythm strip. Some or all of these assessments may be at outside facilities.

Medical Assessments
The physical exam must be performed by a qualified physician and lab assessments must be completed at a designated lab. Medical assessments will include:

- Blood pressure, pulse, and body temperature measurement.
- Height and weight, which will be used to calculate Body Mass Index (BMI).
- Examination of head, eyes, ears, nose, throat, skin, heart, lungs, abdomen, and extremities.
- Brief neurological exam (cranial nerves 2 to 12, sensory, motor, reflexes, and cerebellar function).
- ECG and 1-minute rhythm strip.
- Clinical laboratory assessments, per Section 13.0 Clinical Laboratory Assessments. The clinical laboratory values will not be captured in the eCRF but will be used to establish eligibility and will be kept with the participant's source record. Clinically significant abnormal values will be captured as medical history.
- If there is evidence of liver disease by history, physical examination or laboratory testing, HCV serology will be performed.
- If there is evidence of significant hepatic disease other than HCV, the potential participant will not be eligible for enrollment and will be advised to see their personal physician for further evaluation. If HCV serology is positive and the potential participant has not already been evaluated for possible treatment of HCV, they will be referred to a physician with expertise in evaluating and treating liver disease. After this evaluation and after completion of any recommended treatment, if the HCV is judged by this physician to be relatively stable and of mild severity, the participant may be enrolled, if there are no other contraindications.
- If the potential participant has a condition such as controlled hypertension or Diabetes Mellitus (Type 2) that warrants additional testing to ensure they do not have evidence of significant vascular or other cardiac disease, they have no other evidence of cardiovascular or cerebrovascular disease by history, physical exam or ECG, and if the investigator judges their overall health and other cardiovascular risk factors to be acceptable (family history, smoking, lipid levels, body weight, level of physical activity), they will be referred for nuclear exercise testing by a cardiologist and for carotid ultrasound. If these tests fail to reveal evidence of significant vascular disease or other cardiac disease, the person may be enrolled if there are no other contraindications. Participants taking one or more antihypertensives may be enrolled in the study. The investigators will record and review medications used to control hypertension prior to enrollment.

Additional visits (in person, by telephone, or via telemedicine) may be scheduled at the discretion of the study staff to collect more information for determining eligibility or to discuss study expectations with the potential participant.

Once all results are obtained, the site team will review all medical assessments, notes from interviews and discussions, medical records, and measures against eligibility criteria. If, upon examination, there are questions raised about possible medical problems, the site physician will request additional tests, assessments, or measures as indicated. The site physician may also

contact outside providers with participant permission as needed. If deemed initially eligible, the site staff will schedule the IR screening Although the IR visit is by telemedicine, the participant will be provided a location to complete the telemedicine visits at the study site if needed. For the first IR assessment, the site can provide technical support before the assessment and therapeutic support after, if needed. The site staff will instruct the participant on how to access the telemedicine visits going forward. For all IR visits discuss with the participants that they should have adequate internet access and be in a private and quiet space where they are comfortable talking about personal matters.

8.1.2 Independent Rater Screening

If participants meet initial eligibility during Screening, a blinded IR will continue the eligibility assessment via telemedicine after reviewing the results of the screening measures. The blinded IR interview may be recorded to assess reliability of ratings. If possible, the potential participant should be present at the study site during this assessment, in case the therapy team is needed for support. If a participant reports suicidal ideation during this assessment, the IR will contact the therapy team after the call and present any concerns. The therapy team will follow-up with the participant to ensure safety, provide support, recommend treatment, or schedule a visit to the study site.

The results from the IR screening measures will be provided to the therapy team at the site to review along with all other Screening information to determine eligibility. Items assessed by the IR at this visit will be confirmed in the Preparatory Period by clinical observation, but the measures will not be repeated. If site staff deem the participant eligible, schedule Enrollment.

IR visits will be assigned based on availability.

8.1.3 Enrollment

In advance of enrollment, the site team will review all notes from Screening visits, medical assessments, IR assessments, notes, discussions, medical records, and measures against eligibility criteria. If the participant is eligible, medication tapering and concomitant medications dose adjustments will be discussed, if applicable. The site physician will consult the prescribing physician to initiate medication tapering for participants. For all details on concomitant medications, tapering, allowed, and prohibited medications refer to Section 12.0 Concomitant Medications.

At study onset at each site, if a potential participant is eligible, the study team will contact the Medical Monitor and send a summary of the medical history for approval to enroll the potential participant. After the Medical Monitor establishes confidence in the enrollment procedures at each site, the Medical Monitor will inform the site that for future participants they need only contact the Medical Monitor if they have questions about further participants' eligibility. If a participant is approved by the sponsor, the participant will be notified of enrollment in-person, via telemedicine, or by telephone. Medical history and medication information will be reviewed for completeness. A medication tapering plan will be discussed with the participant, if applicable. If agreeable, the participant will be enrolled in the study. Once enrolled, AE collection requirements begin (refer to Section 11.0 Safety). Enrollment should take place 2 to 14 days after Independent Rater Screening is completed. Enrollment and the first Preparatory Session may take place on the same day.

8.2 Preparatory Period with Enrollment Confirmation

Participants will undergo three Preparatory Sessions lasting approximately 90 minutes with the therapy team prior to the first Experimental Session. The Preparatory Period will be initiated within 12 days of Enrollment and last 1 to 11 weeks, depending on duration of medication tapering. There must be at least 48 hours between Preparatory Sessions. The minimum time to complete the Preparatory Period is 5 days. The Preparatory Period will also include the Baseline CAPS-5 assessment, which will be assessed by a blinded IR. Adherence criteria for Preparatory Sessions should be followed per the Treatment Manual. In these visits the therapy team will work with the participant to prepare for MDMA-assisted psychotherapy, begin building therapeutic alliance, and promote a safe set and setting for confronting trauma-related memories, emotions, and thoughts.

8.2.1 Preparatory Sessions 1 and 2

Preparatory Sessions during the Preparatory Period will focus on psychoeducation about PTSD, building safety for the therapeutic relationship, developing the therapeutic alliance, obtaining the background for the trauma, and preparing the participant for the first Experimental Session. Telephone calls may be scheduled between visits if indicated for tapering, safety, or any further questions about medical history.

The Preparatory Sessions will be scheduled as follows:

- Preparatory Session 1 will occur 1 week (0 to 12 days) after Enrollment. The visit timing should take in to account appropriate times for monitoring medication tapering.
- Preparatory Session 2 will occur within 3 weeks of Enrollment and at least 2 days after Preparatory Session 1. The visit timing should take into account appropriate times for monitoring medication tapering.
- If tapering is ongoing at Preparatory Session 2, the site team will schedule a telephone phone call within 7 days of completion of tapering and stabilization to confirm that the participant is eligible for the Baseline CAPS-5 assessment. If tapering is complete or not needed, the site team will confirm eligibility and schedule Baseline CAPS-5 and Preparatory Session 3 as soon as possible.

At each 90-minute psychoeducation and psychotherapy Preparatory Session, the therapy team will:

- Record the therapy session.
- Inquire about any possible changes in health to ensure the participant continues to meet all eligibility requirements. Record AEs as described in Section 11.0 Safety.
- Inquire about concomitant medication use and adherence.
- Confirm that medication tapering is ongoing or complete, as appropriate.
- Discuss goals and expectations for the Experimental Session, following standard procedures and techniques described in the Treatment Manual [106].

If a participant would like a companion present during or after the Experimental Session, a meeting between the therapy team and that individual will be scheduled prior to the first Experimental Session. There must be mutual agreement between the participant and therapy team concerning the presence of the companion.

During one of the Preparatory Sessions, if possible, the therapy team will introduce the participant to the attendant who will remain with the participant during each overnight stay after

each MDMA-assisted psychotherapy session. The attendant will be an individual with previous training in managing psychological distress. The site will make all attempts to have the same attendant for each Experimental Session for a given participant, but it is not guaranteed.

At any time during the Preparatory Period, if a potential participant is deemed to be ineligible, the site team will classify them as a Pre-randomization Early Termination, notify the potential participant that they are unfortunately not eligible for the study, and not schedule additional assessments. The site team will not randomize participants classified as Pre-randomization Early Terminations.

8.2.2 Baseline: CAPS-5 by Independent Rater

An IR will measure the Baseline CAPS-5 via telemedicine as soon as possible after medication tapering, stabilization, and two Preparatory Sessions are complete. This visit may be recorded to video to establish inter-rater reliability. The scores will be sent as soon as possible to the site staff.

Due to possibilities of negative side effects of withdrawal from psychiatric medications, the Baseline CAPS-5 should be scheduled as close to the end of tapering as clinically appropriate so that participants who do not meet eligibility criteria can resume their previously prescribed medications as quickly as required for symptom management and participants who do meet criteria can be enrolled and treated as quickly as possible.

8.2.3 Baseline: Preparatory Session 3 & Enrollment Confirmation

Prior to Preparatory Session 3, the site team will review the results of the IR visit Preparatory Session 3 should be scheduled 3 to 6 days after the Baseline CAPS-5 assessment and 1 to 4 days before Experimental Session 1.

At Preparatory Session 3, the site team will confirm eligibility by reassessing specified eligibility criteria and ensuring that the participant continues to agree to all lifestyle modifications. If the participant continues to be eligible for the study, enrollment will be confirmed (after approval from the Medical Monitor for the initial participants until confidence is established). If any requirements are not met before or during Preparatory Session 3, the participant will be considered a Pre-randomization Early Termination.

For eligible participants at Preparatory Session 3, qualified site staff will:

- Perform urine pregnancy test for participants who are of childbearing potential.
- Perform urine drug test.
- Administer Since Last Visit C-SSRS to determine suicidal risk.
- Actively support participant in the completion of Baseline self-reported measures. Completion of measures does not need to be recorded.
    - ACE
    - BDI-II
    - CPGS
    - EQ-5D-5L
    - IASC
    - IPF
    - SCS
    - TAS-20
    - SRNU

  - EAT-26
  - HPQSF
  - UFEC
- Complete the third 90-minute Preparatory Session (as described in Section 8.2.1 Preparatory Sessions 1 and 2) with the purpose of confirming all enrollment is met and completing final preparation for the first Experimental Session.
- Remind the participants of lifestyle modifications, including fasting and refraining from using psychoactive or non-approved medications, pertinent prior to the Experimental Session per Section 4.3 Lifestyle Modifications.
- Schedule Experimental Session 1.

8.3 Treatment Period

During the Treatment Period, which occurs over a duration of 9 to 15 weeks participants will complete three treatments. Each treatment consists of an Experimental Session, followed the morning after by an Integrative Session, phone follow-ups over the next week, a second Integrative Session within 2 weeks, and a third Integrative Session within 3 to 5 weeks. The Experimental Sessions will be scheduled 3 to 5 weeks apart.

8.3.1 Experimental Sessions

There will be three blinded Experimental Sessions Procedures. for MDMA-assisted psychotherapy will remain the same across all sessions and all procedures regardless of dose received. Experimental Sessions must be at least 8 hours long, measured from 30 minutes prior to IMP administration.

Experimental Sessions will be scheduled as follows:

- Experimental Session 1 will occur 1 to 4 days after Preparatory Session 3 and Enrollment Confirmation. The first Experimental Session will include 80 mg of IMP followed by a supplemental half-dose 1.5 to 2 hours after the initial dose unless contraindicated.
- Experimental Session 2 will occur 21 to 35 days after Experimental Session 1 and at least 1 day after Integrative Session 1.3. A dose of 80 or 120 mg MDMA or placebo will be administered. A supplemental half-dose will be administered 1.5 to 2 hours after the initial dose unless contraindicated.
- Experimental Session 3 will occur 21 to 35 days after the second Experimental Session and after Integrative Session 2.3. A dose of 80 or 120 mg MDMA or placebo will be administered. A supplemental half-dose will be administered 1.5 to 2 hours after the initial dose unless contraindicated.

Table 4: Schedule of Procedures for Experimental Sessions

Approximate Time	Procedure or Action
9:30	Urine drug screen and pregnancy test, concomitant medication information collected, participant acclimated to environment, C-SSRS
9:55	Baseline BP (Blood Pressure), body temperature, pulse
10:00	**IMP Administration**, Begin video recording
11:30	BP, body temperature pulse<br>**Supplemental Dose Administration**, unless contraindicated
17:30	C-SSRS, BP, body temperature, pulse

Pre-IMP administration

- 24 to 48 hours prior to the first Experimental Session, the participant will be randomized using a web-based randomization program.
- 24 to 48 hours prior to each Experimental Session, the site team will obtain the container assignment.
- On the day of the Experimental Session, the participant will arrive approximately 30 to 60 minutes prior to IMP administration.
- The site team will ensure the participant has not used caffeine or nicotine 2 hours prior and has fasted for 10 hours prior to IMP administration and complied with all other requirements per Section 4.3 Lifestyle Modifications.
- The site team will inquire about any possible changes in health to ensure the participant continues to meet all eligibility requirements and record AEs as described in Section 11.0 Safety.
- The site team will instruct the participant that they will not be able to use caffeine or nicotine at least 6 hours after the IMP administration.
- The site team will complete urine drug screen, pregnancy test, and concomitant medication review.
  - A positive drug screen will be reviewed by the site physician and may be cause for delaying IMP administration to a later time, rescheduling the session to a later date, or withdrawing the participant from the study, based on Medical Monitor review.
  - A positive pregnancy screen is cause for withdrawal from the protocol.
- The therapy team will administer Since Last Visit C-SSRS.
- The therapy team will review procedures for the Experimental Session with the participant and discuss the participant’s goals, intentions, and concerns and some of the commonly experienced effects of MDMA. The choice of whether to keep the dose the same or change it from the first Experimental Session will be made by the therapy team in consultation with the site physician based on observed response, tolerability to the previously administered dose, and discussion with the participant.
- If the participant continues to be eligible, the session will proceed.
- Baseline blood pressure, body temperature, and pulse will be measured just prior to administration of the initial dose.

During the Experimental Session

- After video recording has begun, at approximately 10:00 in the morning, a qualified staff member will administer the initial dose of IMP with an electrolyte-containing fluid. The participant will sit or recline on comfortable furnishings. Eyeshades and a program of music will be provided for the participant if they wish to use them. Whenever they wish, participants may speak to the therapy team, who will provide guidance and support, as needed.
- After the first hour, if the participant has not spoken spontaneously, the therapy team will check in with them about the nature of the experience. For the rest of the experience, as appropriate, the therapy team will support and encourage the participant in emotional processing and resolution of whatever psychological material is emerging, as described in the Treatment Manual.
- Electrolyte-containing fluids will be provided throughout the session but not to exceed three liters overall.
- Blood pressure, body temperature, and pulse will be measured approximately 1.5 to 2 hours after the initial dose, before the supplemental dose is administered.

- If the participant prefers not to have the supplemental dose, the therapy team will document the reason.
- The site physician will be contacted with a brief description of how the session is progressing and the recent vital signs. The site physician will approve or deny the administration of the supplemental dose. If an AE requiring medical attention has occurred between the initial and supplemental dose, the site physician will determine whether the supplemental dose is contraindicated. If medical attention is needed, the site physician will provide further instruction or consult the Medical Monitor.
- A supplemental half-dose will be administered with a glass of electrolyte-containing fluid approximately 1.5 to 2 hours after the initial dose, unless contraindicated.
- Food will be provided during the latter part of the session.
- If there is an approved companion, that person may arrive as agreed upon but will wait in the waiting room until a member of the therapy team brings them to the session room. Alternatively, the companion may arrive after the session has ended.

End of Experimental Session

- The therapy team will administer Since Last Visit C-SSRS.
- The therapy team will record AEs and concomitant medications.
- The session may be ended if all medical and psychiatric parameters are acceptable, elevations in vital signs have resolved to pre-IMP levels, the participant is alert, ambulatory, and emotionally stable, and the night attendant has arrived.
- The therapy team or site physician shall remain available to participants via 24-hour cellular phone for integration, as needed.

Overnight Stay (See Appendix A for sub-study with exceptions)

- Participants will remain overnight in an appropriately furnished room at or near the study site until after the Integrative Session the morning after each Experimental Session. The stay will be in an appropriately furnished room at the study site. With prior approval of the therapy team, a companion may accompany the participant during the overnight stay.
- An attendant will check in periodically on the participant during the overnight stay, even if a companion is present. The attendant will monitor participant condition and will help participants relax during the overnight stay. The attendant will be an individual with some previous training in managing psychological distress and will be supportive but not intrusive. If there is an emergency or the participant needs additional support, the attendant can contact the therapy team.
- The participant and a companion (if applicable) will receive information that will allow them to contact the therapy team during the overnight stay in the case of an emergency or to request for additional support.
- Participants will be encouraged to use much of the time during their overnight stay for rest and as a period of reflection and integration in a quiet atmosphere.
- The participant will be driven home after the integration session by either a driver arranged by the participant, by site personnel, or taxi.

8.3.2 Telephone Contact After Experimental Sessions

The goal of the telephone contact is to assess health changes, ensure participant safety, and offer support. The therapy team will follow-up with the participant by telephone on Days 2, 4, 6, 7, 8, 10, 12, and 14 after each Experimental Session. Each call will last on average 5 to 20 minutes but could be longer to address participant concerns and to adequately assess wellbeing. Additional

telephone contact can be initiated at the request of the therapy team or participant. If any planned phone contact would occur on the date of an Integrative Session, the phone contact does not need to be completed.

At each telephone contact, the therapy team will:

- Inquire about any possible changes in health, assess the participant's mental health and the status of any previously recorded AEs, and record AEs as described in Section 11.0 Safety.
- Inquire about concomitant medication use and adherence.
- Offer support in accordance with the Treatment Manual.
- Administer the Since Last Visit C-SSRS.

8.3.3 Integrative Sessions

After each Experimental Session, three Integrative Sessions will take place. Each session will consist of 90 minutes of psychotherapy. Integrative Sessions should not be less than 48 hours apart.

Treatment 1

- Integrative Session 1.1 morning after Experimental Session 1
- Integrative Session 1.2: 3 to 14 days after Experimental Session 1
- Integrative Session 1.3: 20 to 34 days after Experimental Session 1 and 1 to 7 days in advance of Experimental Session 2. This visit serves two purposes: to continue integration and to prepare for the next Experimental Session. The participant will complete the LEC-5 self-report measure.

Treatment 2

- Integrative Session 2.1: morning after Experimental Session 2
- Integrative Session 2.2: 3 to 14 days after Experimental Session 2
- Integrative Session 2.3: 20 to 35 days after Experimental Session 2 and 1 to 7 days in advance of Experimental Session 3. This visit serves two purposes: to continue integration and to prepare for the next Experimental Session. The participant will complete the LEC-5 self-report measure.

Treatment 3

- Integrative Session 3.1: morning after Experimental Session 3
- Integrative Session 3.2: 3 to 14 days after Experimental Session 3
- Integrative Session 3.3: 21 to 35 days after Experimental Session 3 This visit will be the final Integrative Session prior to entering the follow-up period. The participant will complete the LEC-5 self-report measure.

During Integrative Sessions, the therapy team will:

- Record the session.
- Inquire about any possible changes in health. Assess the participant's mental health and the status of any previously recorded AEs. Record AEs as described in Section 11.0 Safety.

- Inquire about concomitant medication use and adherence.
- Administer Since Last Visit C-SSRS to determine suicidal risk.
- Discuss and review events that occurred with the participant during the Experimental Session, including thoughts, feelings, and memories. If necessary, the therapy team will help the participant to reduce any residual psychological distress they are experiencing. The therapy teams will also encourage the transfer of states of acceptance, feelings of intimacy, closeness, and reduced fear experienced in Experimental Sessions to emotionally threatening everyday situations. The therapy teams will be supportive, validate the experience, and facilitate understanding and emotional clearing.
- Be accessible for additional support via phone or telemedicine if needed.
- At each third Integrative Session, direct the participants to complete the LEC-5.

8.3.4 Independent Rater Assessments

A blinded IR from the IR Pool will conduct the assessments via telemedicine. These assessments may be recorded to establish inter-rater reliability. The results will not be sent to site staff but instead will be sent to a blinded IR Coordinator to enter the data which will be blinded to sponsor and site personnel. Participants will be instructed to withhold their opinion on treatment group assignment or the number of study visits they have completed from IRs. The IR will also administer the DSP-I and SDS at these time points.

8.4 Follow-up Period and Study Termination

8.4.1 Follow-up Period

After the last Integrative Session 3.3, participants will enter follow-up for approximately 4 weeks (±2 weeks) with no protocol required visits until the final CAPS-5 assessment followed by Study Termination Visit. Participants will have access to therapy teams for support if needed, and additional visits via phone, telemedicine, or in person can be scheduled if requested. Participants will continue to comply with protocol requirements for concomitant medications until after Study Termination.

8.4.2 Study Termination

Study Termination will take place approximately 4 weeks after Integrative Session 3.3. Participants who have withdrawn from treatment but have continued for follow-up will also complete this assessment immediately upon withdrawal.

The site team will:

- Inquire about any possible changes in health. Assess the participant's mental health and the status of any previously recorded AEs. Record AEs as described in Section 11.0 Safety.
- Inquire about concomitant medication use and adherence.
- Administer Since Last Visit C-SSRS.
- Measure weight (used to calculate BMI).
- Measure blood pressure.
- Provide and discuss a study exit plan.

- Ask them to enroll into the LTFU extension study with a visit at 12 months after the last Experimental Session. Review contact information and Informed Consent procedures for LTFU.
- Review potential timeline until study unblinding and how they will be informed of their treatment group at that time.
- Actively support participant in completion of Study Termination self-reported measures. Completion of the measures does not need to be recorded.
  - LEC-5
  - BDI-II
  - CPGS
  - EQ-5D-5L
  - IASC
  - IPF
  - SCS
  - TAS-20
  - AUDIT
  - DUDIT
  - SRNU
  - EAT-26
  - HPQSF

After all Study Termination measures and assessments are completed, the participant is considered terminated from the study. The participant can resume normal everyday life. The study team will provide an Exit Plan, which may include a referral for additional medical or therapeutic care, as described in Section 8.4.3.2 Exit Plan. Eligible participants will be asked to participate in the LTFU extension study, described in Section 8.4.3.1 Extension Studies.

8.4.2.1 Extension Studies

Upon completion of the study, defined as completing at least one Experimental Session and one CAPS assessment beyond Baseline, participants will be asked to join the LTFU extension study. The ICF for this study will be provided to eligible participants at the Study Termination visit. This study will measure outcomes 12 months after the last Experimental Session.

Upon unblinding after database lock, all participants who were assigned to placebo (regardless of final CAPS-5 scores), completed the study, meet criteria, and are in good standing with the clinical site, will be offered the opportunity to enroll in an open-label safety study using the same manualized MDMA-assisted psychotherapy as administered during the study. This open-label safety study will take place at least 18 months after the first participant is enrolled in MAPP1 and will last 19 to 38 weeks, similar to this protocol. Data from this study will be included in the sponsor's overall safety database.

Any sub-studies other than what is outlined in Appendix A will be detailed in separate documents.

8.4.2.2 Exit Plan

At Study Termination, participants will be provided with an Exit Plan. This Exit Plan will summarize treatments completed, current medications, and contact information for more information about the study if needed. Participants may request a referral for further therapeutic or medical care if appropriate. Enrolled participants who terminate the study early will be provided an Exit Plan at their last contact. Screen Failures will be provided a referral if requested.

9.0 Investigational Product

9.1 Description of Active Compounds

The Active Pharmaceutical Ingredient (API) to be used in this protocol is MDMA (as hydrochloride salt). This ring-substituted phenethylamine has a complex pharmacology, but it acts most prominently as a monoamine releaser and re-uptake inhibitor. Its direct actions on serotonergic, adrenergic, and other receptors are considerably lower. Refer to the IB for a comprehensive review of the pharmacology, effects and proposed mechanisms of action of the IMP. Mannitol and a lubricant, such as magnesium stearate, will serve as inactive excipients.

9.1.1 Doses

This study will compare the effects of three blinded manualized Experimental Sessions of psychotherapy assisted by flexible doses of MDMA HCl or placebo. Initial doses per Experimental Session include 80 mg or 120 mg MDMA compounded with mannitol and magnesium stearate or indistinguishable weight placebos comprised entirely of mannitol and magnesium stearate, followed 1.5 to 2 hours later by a supplemental half-dose (40 mg or 60 mg MDMA HCl or placebo). Total amounts of MDMA HCl to be administered per Experimental Session range from 80 mg to 180 mg. All drug is encapsulated with HPMC capsules.

9.1.2 Dose Modifications

In the first Experimental Session, the initial dose will be 80 mg MDMA HCl or placebo. In the second and third Experimental Sessions, the initial dose may be increased to 120 mg MDMA HCl or placebo unless contraindicated. The choice of whether to keep the dose the same or change it from the first Experimental Session will be made by the therapy team the in consultation with the site physician based on observed response, tolerability to the previously administered dose, and discussion with the participant. In each Experimental Session, 1.5 to 2 hours after the initial dose is given, the participant will be administered a supplemental half-dose unless contraindicated. If an AE requiring medical attention occurs between the initial and supplemental dose this will be evaluated as a potential contraindication by the site physician. If a participant prefers not to take the supplemental dose, the reason will be documented. Participants will not know if they have been assigned MDMA or placebo but can indicate if they want the dose to change or remain the same.

9.1.3 Stability

IMP will be manufactured and packaged according to Good Manufacturing Practices (GMP). 6-month accelerated stability studies will be completed for the IMP along with an ambient stability study that will remain ongoing throughout the duration of the study. All required Chemistry Manufacturing and Control (CMC) submissions will be made to the IND.

9.2 Handling

9.2.1 Encapsulation, Packaging, and Labeling

The IMP for each Experimental Session will be packaged in one primary container, labeled with a unique container number, protocol number, blinded IMP name and lot number, sponsor name, Experimental Session number, and a statement that the IMP is restricted to clinical trial use only. All labels will comply with local, state, and federal regulations. The initial and supplemental dose

will be packaged separately within the primary container. There will be one primary container per participant per Experimental Session.

9.2.2 Accountability

Forms will be provided to track IMP accountability and administration throughout the study. Blinded IMP accountability and administration logs will be reviewed during routine monitoring visits or through central monitoring. MDMA will be handled in accordance with all local, state, and federal regulations and forms pertaining to the use of controlled substances, and forms will be maintained by the appropriate controlled substance license holder or delegate.

Each primary container label will contain a unique container number for the IMP assigned to a single Experimental Session. The container numbers will be used to track IMP administration in the Source Record and the IMP administration log. The web-based randomization system will enable tracking of blinded primary containers for accountability purposes.

9.2.3 Storage

MDMA is a controlled substance and will be stored and handled in compliance with all relevant federal and state regulations. In accordance with these requirements, the appropriate license holder or designee will be responsible for storing, dispensing, and administering the MDMA. It will be stored securely in accordance with federal, state, and local regulations.

9.2.4 Administration

IMP will only be removed for a single Experimental Session at a time and will be administered orally at the study site. All doses administered will be recorded on the appropriate accountability and administration logs. Only the initial dose is required to be given at each Experimental Session. Supplemental doses should be administered unless contraindicated. Each dose (initial and supplemental) will be administered with a glass of electrolyte-containing fluid.

A person at the site authorized to manage and administer controlled substances will dispense the appropriate container for each Experimental Session. If a supplemental dose is not administered, the unused IMP will be kept for accountability.

Records pertaining to the use of scheduled, regulated compounds will be maintained in accordance with relevant federal and state regulations.

9.2.5 Treatment Compliance

Compliance to protocol required doses will be guaranteed by the person licensed to manage and administer controlled substances for Experimental Sessions at each site. All administered doses will be recorded for IMP accountability. The IMP will be stored securely per regulations.

9.3 Randomization and Participant Numbering

Every potential participant who is prescreened by telephone according to the IRB-approved script will be assigned a seven-character alphanumeric Screening Number and recorded on the Screening Log. This number will begin with ‘S5’ to identify that it is a Screening Number. The next two digits represent the site number (e.g., ‘11’), followed by a three-digit screening identifier starting with ‘501’. The screening identifier will be assigned to each prescreened participant at a

site sequentially. For example, the first Screening Number at Site 11 will be S511501 and first Screening Number at Site 12 will be S512501.

Each participant who passes Screening and is enrolled in the trial at Visit 0 will be assigned a seven-digit Participant Number. The first two digits will be ‘11’. The next two digits represent the site number (e.g., ‘11’), followed by a three-digit participant identifier starting with ‘001’. The Participant Number will be assigned to each enrolled participant at a site sequentially. For example, the first Participant Number at Site 12 will be 1112001. Eligible participants will be enrolled in the study and sequentially assigned an identification number. All participants will also be assigned a unique participant identifier within the database for use in analysis.

Participants will be randomized in 1:1 allocation in a blinded fashion to the MDMA-assisted psychotherapy group and the placebo plus psychotherapy group, stratified by clinical site. Randomization will be managed via an Interactive Web Randomization System (IWRS) based on a centralized randomization schedule developed by an independent third-party vendor to maintain blinding.

9.4 Blinding and Bias Minimization

Eligibility will be determined by review of screening by the PI, site team and as needed the sponsor Medical Monitor prior to randomization. Participants, site staff, and the sponsor will remain blinded to subject group assignment until after the database is locked.

To further minimize bias in measuring efficacy, the sponsor will use an observer-blind, centralized, reliable IR pool to administer the Primary Outcome measure via live video interviews. The IR Pool will have no knowledge of AEs. The IR Pool is blinded to full study design, visit number, treatment assignment, number of treatments, and any data from the treating therapy team after Baseline. IRs will be assigned to participants based on availability. Participants will be instructed to withhold their opinion on treatment group assignment from IRs. The participants, sponsor, and site staff will not see the CAPS-5 results until the end of the study. Changes in study management and analysis, if any, will be made by sponsor personnel who are blinded to group assignment and CAPS-5 data.

To ensure that all participants regardless of group assignment are treated in a similar manner, the sites will be required to follow the protocol and Treatment Manual delineating minimum length of time per visit type and describing delivery of treatment. All Experimental Sessions are required to be at least 8 hours long. Adherence to the Treatment Manual will be randomly checked by review of video by blinded Adherence Raters. The sponsor will monitor data in real-time to ensure complete data collection for all participants, including those who discontinue treatment. Sites will be required to make and document a specific number of attempts to obtain follow-up data per protocol. All randomized participants who receive at least one dose of IMP and complete at least one follow-up assessment will be included in the final mITT primary efficacy analysis.

If there is an emergency requiring knowledge of a participant’s condition assignment, the blind may be broken for an individual participant through the IWRS. Upon database lock, the study will be unblinded and participants will be notified of treatment assignment.

10.0 Risks

10.1 Non-drug Related Risks

10.1.1 Medical Assessments

In preparation for MDMA-assisted psychotherapy sessions, blood draws and a full medical examination, including a physical examination, ECG, 1-minute rhythm strip, and laboratory tests, are required to establish eligibility for the study. Temporary discomfort, inflammation, or infection could arise as a result of sampling blood at the punctured vein. Submitting to a full medical examination may also cause discomfort or psychological distress. Since medical examinations and blood draws are required to establish eligibility for the study, they cannot be omitted from the protocol.

10.1.2 PTSD, Suicide Risk, and Psychotherapy

During Screening, throughout MDMA-assisted psychotherapy, and during assessment of study measures, participants will be asked to think about and discuss their thoughts and emotions relating to the traumatic event or events. They may experience intense emotional responses or suicidal ideation as a result of recalling and speaking about this material. Even in a therapeutic context, thinking about and discussing the trauma, symptoms related to the trauma or the effects of PTSD on life function can produce distress and exacerbate suicidal ideation during and immediately after psychotherapy sessions. Psychotherapy is conducted as part of this study, and people undergoing psychotherapy are expected to confront unpleasant thoughts, feelings and memories in the process. Because psychotherapy is an integral part of the research study design, the potential distress arising from psychotherapy is unavoidable. Therapy teams will provide emotional support to participants during any psychological distress.

The therapy team will minimize risks by carefully evaluating all participants to determine if there is a current risk of suicidal behavior. Participants with a history of suicide attempts will not be excluded unless significant risk of suicidal ideation and/or behavior is detected, per Exclusion Criteria described in Section 4.2. Participants will be enrolled according to the Eligibility Criteria based on the clinical judgment of the site physician, therapy team, and Medical Monitor.

A qualified individual will administer the C-SSRS as defined by the study protocol, and as needed depending on clinical presentation of the participants, to monitor for development and intensity of suicidal ideation and/or behavior. The therapy team will implement the following plan to assess elevated or imminent suicide risk.

If the Since Last Visit C-SSRS reveals current serious Suicidal Ideation (scores of four or greater), indicating risk at the time of the assessment, or positive Suicidal Behavior (scores of one or greater), the participant will be referred for further management as described below.

1. If the participant has current suicidal ideation, but no specific plan to commit suicide (Suicidal Ideation Score=4), the individual administering the C-SSRS will ensure:
    a. The participant is evaluated by the investigator and/or site physician to determine an appropriate course of action. Findings will be discussed with the participant and their personal therapist, if applicable.
    b. Regular check-ins via phone or in-person will be continued until the participant has stabilized or a new course of action is taken based on changes in C-SSRS score and/or ongoing clinical assessment.

  c. Notification of the sponsor within 24 hours of this event and provide a narrative for the AE for expedited reporting to FDA. Increases in suicidality will be captured as an AESI per Section 11.1.1 and evaluated for seriousness. SAEs will be reported per regulatory guidance.
  d. Treatment would be continued when deemed appropriate by the investigator and Medical Monitor, unless it is determined that treatment should be discontinued, in which case the participant will enter follow-up.

2. If the participant has suicidal ideation, and a plan to commit suicide (Suicidal Ideation Score=5) or positive Suicidal Behavior (Score greater than or equal to 1), the individual administering the C-SSRS will assess whether the risk is imminent. A Suicidal Ideation score of five does not necessarily indicate an immediate risk if the thoughts are fleeting, fairly easily controlled, and deterrents are strong. If there is no imminent risk, the individual will follow the procedure described above. If there is imminent risk of suicidal behavior, the individual will ensure:
   a. Participants are evaluated by the investigator or site physician to determine an appropriate course of action, and the therapy team will contact their personal therapist, who will be invited to come to the study site to assist, depending on their location.
   b. If it is determined that the participant is at imminent risk of suicide, the therapy team will do one of the following:
      i. Escort the participant to the ED (Emergency Department);
      ii. Escort the participant to an appropriate mental health services facility (e.g., hospital psychiatric unit); or
      iii. Call EMS (Emergency Medical Services) and ensure that the participant is transferred to the responding medical personnel.
   c. If the participant will not comply and wishes to leave without consultation, call EMS. Explain that the participant is in immediate danger of committing suicide. Provide a complete description of the participant and give any other needed details to ensure the participant's safety.
   d. Notification of appropriate members of the study team and sponsor representatives within 24 hours, provide a narrative for the AE for expedited reporting to FDA.
   e. The event will be collected as an AESI per Section 11.1.1 and seriousness will be evaluated. SAEs will be reported per regulatory guidance.
   f. Treatment would be continued when deemed appropriate by the investigator and Medical Monitor, unless it is determined that treatment should be discontinued, in which case the participant will enter follow-up.

10.1.3 Recorded Content

All psychotherapy sessions and IR assessments may be recorded for research and training purposes. Participants may feel uncomfortable with having their sessions recorded. The recordings are necessary for developing the experimental treatment and assessing adherence to the Treatment Manual. Any requests for use of video outside of research and training requests will result in participants receiving information on the request. They will have control over any presentation of this material beyond viewing by researchers or regulatory agencies.

The sponsor uses encrypted, secure technology to transfer and store recordings, but there is always a risk of a security breach. The sponsor is committed to taking preventative measures to avoid such an event. In the case of a security breach, the participant will be notified, and all efforts will be made to minimize the dissemination of recorded content.

10.2 Risks of Receiving MDMA

Study procedures and eligibility criteria have been developed based on Phase 2 clinical trials to exclude potential participants with pre-existing exclusionary medical conditions that would exacerbate risk. The therapy teams and site physicians will be available via mobile phone throughout the study if any problem occurs when a participant is not at the site. In the event of a medical emergency or any other medical problem during an Experimental Session, the site physician will be immediately available by telephone, and based on assessment of the situation, they will make the decision to either evaluate the participant themselves at the site, have the therapy team call EMS to transport the participant to the ED, or instruct the therapy team to take the participant to the ED.

Further information on the risks associated with MDMA can be found in the IB and risk mitigation procedures are described by risk category below. Risk Categories were determined by review of possible risks within the Risk Assessment and Categorization Tool (RACT).

10.2.1 High Level Risks

High Risk does not indicate an event is more likely to happen but indicates per the RACT assessment that new and or more complex procedures are required in the study to ensure screening is adequate to eliminate or manage the risk in the patient population. No high level risks have been identified in this study.

10.2.2 Medium Level Risks

Medium Risk does not indicate the likelihood the event will occur but indicates per the RACT assessment that new or many procedures, which are not complex, are needed to ensure screening is adequate to eliminate or manage the risk in the patient population.

10.2.2.1 Cardiovascular and Cerebrovascular Risks and Mitigation

MDMA is known to transiently increase heart rate and blood pressure in a dose-dependent manner that is generally not problematic for physically healthy individuals. These changes should last no more than 8 hours. Participants with PTSD in MAPS-sponsored Phase 2 studies do not appear to differ from healthy individuals in this sympathomimetic, physiological response. Most people do not experience elevations in cardiovascular parameters that exceed those seen after moderate exercise. An examination of safety data drawn from Phase 2 studies of MDMA-assisted psychotherapy detected a dose-dependent increase in systolic blood pressure but not diastolic blood pressure. Characterization of sympathomimetic effects among participants with controlled hypertension is ongoing.

Risks posed by elevated blood pressure will be addressed by excluding people with pre-existing uncontrolled hypertension and monitoring blood pressure and pulse, as described in Section 4.2 Exclusion Criteria. Before and after IMP administration in Experimental Sessions, the therapy team will monitor vital signs. The therapy team will attend to clinical signs and symptoms of potential rare complications of the cardiovascular effects of MDMA, such as stroke or acute myocardial infarction (AMI) during Experimental Sessions. Any symptoms such as chest pain, shortness of breath, neurological deficit or confusion or other potential indicators of end organ effects will prompt additional vital sign measurements, and intervention if appropriate. The therapy team will notify the site physician for evaluation if this occurs.

If any participant has neurological deficits, as assessed by the site physician, whether or not they are associated with hypertensive crisis, they will be monitored, as described above and transported to the hospital if medically indicated. If evaluation at the hospital reveals a nonhemorrhagic stroke, there will be sufficient time to administer recombinant tissue plasminogen within the 3-hour time frame recommended in the American Academy of Neurology/American Heart Association guidelines [107, 108].

If a participant experiences ischemic type chest pain, whether or not it is associated with hypertensive crisis, they will be given 0.4 mg of sublingual nitroglycerin every 5 minutes as needed for chest pain pending transport to the hospital, and the site physician will be promptly notified. Nitroglycerin should be stocked on site per applicable national and local regulations in order to respond promptly in case of need. If further evaluation at the hospital reveals that the participant has had an AMI, they will be well within the time frame required for definitive therapy. The American College of Cardiology/American Heart Association guidelines for the treatment of AMI recommend percutaneous transluminal coronary angioplasty (PTCA) as the treatment of choice when it can be performed within 90 minutes of arrival at the hospital in participants who present within 12 hours of an episode of chest pain lasting more than 30 minutes and who have ECG evidence of AMI [109]. Any participant who experiences such medical complications during an Experimental Session will not be given another Experimental Session, unless it is approved by the investigator, site physician, and the Medical Monitor.

As the characterization of QT effects for the API is ongoing, QT interval will be evaluated in the event of hospitalization for management of cardiovascular or cerebrovascular event. If at any time a participant develops a QT/QTc interval>500 ms or of>60 ms over Baseline during ECG evaluation, the participant will be discontinued from treatment.

10.2.2.2 Psychological Risks and Mitigation

Mild anxiety and depressed mood are occasionally reported 1 to 3 days after MDMA administration [49, 110]. Psychological distress from MDMA could arise from the first indications of MDMA effects until the last effects have dissipated or even later. Anxiety or distress during the session may last for as little as 5 minutes or for as long as 5 hours or more. In addition, psychological distress could arise following an Experimental Session as a result of participants having difficulty integrating their experience after the MDMA effect has subsided. In previous Phase 1 and Phase 2 studies, these symptoms have been self-limiting and have responded well to reassurance from the therapy team, with occasional use of benzodiazepines for anxiety. In this study, participants will have the intention of confronting and working through traumatic experiences. Accordingly, signs of psychological distress, panic, or other unpleasant psychological reactions are to be expected and may be considered an element of the psychotherapeutic process.

Proper preparation and follow-up support will reduce the difficulties participants might have with acute or sub-acute reactions. The potential for destabilizing psychological distress will be minimized by:

- Excluding people who might be more vulnerable to it.
- Preparatory Sessions of non-drug psychotherapy before the Experimental Session.
- Creating an atmosphere of trust during the Experimental Session.
- Close monitoring.
- Phone contact with participants during the week after the Experimental Session.
- Integrative Sessions.

- Overnight stays at the study site for the night of each Experimental Session. Qualified personnel will be available during the overnight stay to respond to the needs of the participant. Attendants will be instructed to contact the therapy team upon request or at the appearance of signs of a potential Serious Adverse Event.

During the Preparatory Sessions, participants will be made aware of the fact that difficult emotions, including grief, rage, fear, or panic, may arise during Experimental Sessions. Every effort will be made to help participants resolve difficult symptoms and to arrive at a more comfortable and relaxed state by the conclusion of the Experimental Session, including empathic listening on the part of the therapy team and performance of diaphragmatic breathing by participants.

If the participant is severely agitated, anxious, in danger of self-harm or suicide, or is experiencing any other severe psychological distress, at the end of a psychotherapy session, at least one member of the therapy team will remain with the participant for at least 2 more hours. During this time, the therapy team will employ affect management techniques, will talk with the participant to help them gain cognitive perspective of their experiences, and will help the participant implement the self-soothing and stress inoculation techniques presented during the Preparatory Sessions. If the participant remains severely anxious, agitated, in danger of self-harm or suicide, or is otherwise psychologically unstable at the end of the 2-hour stabilization period, the site physician and therapy team will decide between the following options:

1. If severe distress occurs at the end of an Experimental Session, a psychiatric nurse, therapeutic assistant, physician, or therapy team member will stay with the participant until the severe distress resolves or until the time of their Integrative Session appointment the following morning. The therapy team will then meet with the participant daily until the period of destabilization has passed.
2. If the participant experiences severe, persisting emotional distress, such as panic attacks, severe generalized anxiety, or insomnia following an Experimental Session, a licensed therapy team member or the site physician may prescribe a benzodiazepine (specifically, lorazepam) and/or sleep aid (e.g., zolpidem). The route, frequency, and dose should be determined by the site physician. If these medications are stocked on site, they should be stored according to applicable national and local regulations. This medication will be captured on the Concomitant Medications eCRF. The site physician should not prescribe an SSRI, SNRI, or monoamine oxidase inhibitor (MAOI) in this context, unless it has been determined that the participant will be withdrawn from the study. Residual symptoms will be addressed during the frequent follow-up psychotherapy visits with the therapy team.
3. If a participant should become psychotic, arrangements will be made to stabilize them or transfer them to the ED if hospitalization is necessary. Any participant who is hospitalized after a severe psychological reaction will be suspended from the protocol until after recovery or stabilization, at which time the investigator and/or site physician will carefully evaluate the participant's emotional status.

For those participants engaged in an ongoing therapeutic relationship with a psychotherapist or psychiatrist, the participant's outside therapist(s) will be involved in the management of any psychiatric complications. For those participants engaged in an ongoing psychotherapeutic relationship with the investigator or member of the therapy team, the management of any psychiatric complications will be undertaken by them in their capacity as the participant's therapist.

10.2.3 Low Level Risks

Low Level Risk does not indicate the likelihood the event will occur but indicates per the RACT assessment that no new or complex procedures are needed to ensure screening is adequate to eliminate or manage the risk in the patient population.

10.2.3.1 Thermoregulatory Risks and Mitigation

MDMA administered in a controlled setting produces only a slight increase in body temperature [110]. Ambient temperature does not enhance or attenuate this slight elevation in humans. In data gathered from sponsor-supported Phase 2 studies, it was found that compared to placebo, a higher percentage of participants receiving MDMA had peak body temperatures greater than 1 degree Celsius (°C) above Baseline. However, there was no strong relationship between dose of MDMA and peak body temperature or between MDMA dose and elevation above threshold of 1°C above Baseline.

Ambient temperature will be kept at a comfortable level during Experimental Sessions. If a participant's temperature rises more than 1°C or the participant states that they feel hot, attempts will be made to decrease body temperature and increase comfort by removing blankets and layers of clothing, decreasing the ambient temperature, and, if necessary, directing a fan toward the participant. If at any time the temperature rises more than 1.5°C above Baseline despite these efforts, the site physician will be consulted for further evaluation and treatment.

10.2.3.2 Osmoregulatory Risk and Mitigation

MDMA administered in a controlled setting is not expected to have any risks of osmoregulatory changes. Participants will not be allowed to drink more than three liters of electrolyte-containing fluids over the course of the Experimental Session and fluid intake will be spread out appropriately during the session. If a participant exhibits any signs of toxicity or clinically significant dilutional hyponatremia despite these precautions after an Experimental Session, they will not receive another Experimental Session unless it is approved by the investigator, site physician, and the Medical Monitor.

10.2.3.3 Genotoxicity Risk and Mitigation

To reduce the risk of metabolic activation and formation of nitroso-derivatives of MDMA due to interactions with nitrates or nitrites in food, participants are required to have fasted (no intake other than alcohol-free liquids) for 10 hours prior to IMP administration at Experimental Sessions.

10.2.3.4 Reproductive and Developmental Risks and Mitigation

Risks posed by MDMA to pregnant people are not known. One of two studies of Ecstasy users suggests that use of Ecstasy and other drugs during pregnancy may be associated with some abnormalities at birth while the other failed to find this association [111, 112].

Pregnant and lactating people will be excluded from participation in the study. Participants who are able to become pregnant must have a negative pregnancy screen before undergoing each Experimental Session and must agree to use adequate birth control for the duration of the study during the Treatment Period. Paternal drug exposure studies show that exposure to MDMA was not a factor in adverse pregnancy outcomes, so partners of participants who were assigned male at birth will not be required to practice birth control. Due to the limited number of single-dose

exposures being studied in this trial, the chance of birth defects are low. Procedures described in Section 11.3 Pregnancy have been put in place to mitigate risk of reproductive or developmental exposure to the IMP.

10.2.4 Minimal Risks

Minimum Level Risk does not indicate the likelihood the event will occur but indicates per the RACT assessment that no procedures are needed beyond basic monitoring to ensure screening is adequate to eliminate or manage the risk in the patient population.

10.2.4.1 Common AEs

Common AEs are typically observed during IMP administration, but are transient and diminish as the IMP is metabolized and excreted over the next 72 hours after dosing. Common AEs most frequently reported during Experimental Sessions include muscle tightness in the jaw, lack of appetite, dizziness, and nausea. During the week following treatment, lack of appetite, muscle tightness in the jaw, restlessness, weakness, dry mouth, thirst, impaired gait/balance, and sensitivity to cold may be reported. Severe anxiety, insomnia, fatigue, and depressed mood are commonly reported in PTSD studies in both placebo and MDMA groups. Common AEs are typically self-limiting. Elevations in anxiety and poor sleep respond to management with short-acting low dose benzodiazepines (specifically, lorazepam) or sleep aids as needed, per clinical judgment of the site physician.

10.2.4.2 Potential Neurotoxicity Associated with Ecstasy Use

Some researchers believe that MDMA is neurotoxic in humans even at doses used in clinical trials [113]. However, these claims are based on studies that employed inappropriately high doses of MDMA utilized in animal studies and on human studies comparing the effects of repeated use of Ecstasy, often along with other drugs. Meanwhile, another recently published meta-analysis has taken careful steps to overcome methodological limitations in previous work and found only modest evidence of neurotoxicity [114]. The sponsor has carefully considered the risks of such neurotoxicity and conclude that they are minimal in the proposed study. This conclusion is supported by empirical and toxicokinetic evidence and is consistent with the lack of toxicity reported in previous clinical MDMA studies. It does not appear that MDMA-assisted psychotherapy negatively impacts cognitive function. Neurotoxicity and neurocognitive effects of MDMA were studied in the sponsor's Phase 2 studies and provided sufficient data to conclude that limited exposures to MDMA allowed for adequate time for reversibility of any effects at the synaptic level. Furthermore, limited dosing of MDMA has shown to have no effect on neurocognitive functioning.

10.2.4.3 Abuse Liability

Findings in humans and animals suggests that MDMA possesses moderate abuse potential that is higher than that reported for "classic hallucinogens," like psilocybin, but lower than that reported for psychostimulants, such as cocaine or methamphetamine. In sponsor-supported Phase 2 PTSD studies in 107 participants treated with MDMA-assisted psychotherapy in a controlled clinical setting, 29.9% (32 of 107) of participants had tried Ecstasy at least 6 months prior to enrollment, with U.S. samples demonstrating a higher prevalence of use than international studies. Participants reported using Ecstasy an average of 2.3 (SD:1.43) times. Due to the known association of substance use disorders and PTSD, this sample was likely not representative of the general healthy volunteer population but is congruent with the PTSD population. At long-term follow-up across Phase 2 studies, 8.7% of participants (eight of 92) reported "Ecstasy" use across

studies, with five participants indicating single and not repeated use and three participants indicating two uses. Six of these eight subjects had used Ecstasy prior to study participation. Of these participants, most were attempting to recreate a therapeutic experience, and most did not indicate a desire to repeat this. In addition to self-report data, urine drug screens specific for MDMA were performed at random and 2, 6, and 12 months after the final Experimental Session during one study (MP2, N=12). All were negative, supporting the observation that study participants did not seek out MDMA or Ecstasy after taking part in the study [60]. In addition to data on Ecstasy use at follow-up, AEs were reviewed across Phase 2 studies, the sponsor found an absence of clinically significant AEs supporting drug dependence, intentional drug misuse, and substance abuse, and a low rate (<2%) of secondary terms that reflect acute intoxication.

Based on current information, it does not appear that MDMA-assisted psychotherapy demonstrates signals associated with known abuse liability patterns in a PTSD population when administered in a therapeutic setting under continuous observation in three single-dose sessions. Any abuse potential and diversion is further limited since the IMP is not supplied to the participant to take home and is administered in a restrictive setting. Each investigator responsible for dispensing or administration of the IMP will maintain current registration with authorities with oversight of controlled substances. IMP will be handled following all regulations pertaining to the handling and dispensing of controlled substances within research studies. See Section 11.1.1 for AESI terms.

Within-participant elevation in self-reported Ecstasy use post-treatment will be clinically assessed for signals of abuse and will be monitored during the Long-term Follow-up extension study associated with this protocol.

11.0 Safety

11.1 Adverse Events

In accordance with guidance for the specific study site, Health Canada Clinical Safety Data Management Definitions and Standards for Expedited Reporting International Conference on Harmonisation (ICH) Topic E2A: Guidance for Industry or FDA Safety Reporting Requirements for INDs and BA/BE Studies, an Adverse Event (AE) is defined as any medical occurrence in a participant, including any abnormal sign (e.g., abnormal physical exam or laboratory finding), symptom, or disease, temporally associated with the participant’s involvement in the research, whether or not considered related to participation in the research. This definition includes concurrent illnesses or injuries and exacerbation of pre-existing conditions.

Events related to planned treatments or physician visits for Baseline conditions collected in the medical history will not be collected, unless there is an exacerbation of the condition, in which case they will be actively followed until resolution.

An unexpected AE is one that is not listed in the current IB or an event that is by nature more specific or more severe than a listed event.

The site physician will be responsible for reviewing and confirming all AEs and SAEs collected during the study. The therapy teams will collect AEs during study visits from Enrollment through Study Termination. Participants will be asked directly how they are feeling during each contact, and AEs may be captured spontaneously during psychotherapy sessions, telephone calls, or other correspondence. Completed measures may create suspicion that an AE occurred; in this case, the site staff should follow-up with the participant.

All AEs will be monitored by the therapy team until resolution or, if the AE becomes chronic, a cause can be identified. If an AE is unresolved when a participant terminates from the study, a clinical assessment will be made by the site physician, investigator, and/or Medical Monitor as to whether continued follow-up of the AE is warranted.

The severity of events reported on the "Adverse Events" eCRF will be determined by the site physician as:

- Mild: No limitation in normal daily activity
- Moderate: Some limitation in normal daily activity
- Severe: Unable to perform normal daily activity

The relationship of each AE to the IMP will be determined via analysis and the opinion of the investigator will not be collected, with the exception of SAEs.

11.1.1 Adverse Events of Special Interest

In accordance with guidance for the specific study site, Health Canada Clinical Safety Data Management Definitions and Standards for Expedited Reporting ICH Topic E2A: Guidance for Industry or FDA Safety Reporting Requirements for INDs and BA/BE Studies, the sponsor will pay special attention to a subset of AEs involving cardiac function that could be indicative of QT interval prolongation or cardiac arrhythmias, including *Torsade de pointes*, sudden death, ventricular extrasystoles, ventricular tachycardia, ventricular fibrillation and flutter, non-postural syncope, and seizures. The subset of AEs involving suicide risk under the following terms are also of special interest: suicides, suicide attempts, self-injurious behavior associated with suicidal ideation, and suicidal ideation judged to be serious or severe in the opinion of the investigator. These AEs will be marked in the eCRF with the denotation "Adverse Event of Special Interest" (AESIs) whether serious or non-serious.

In order to assess signals of abuse potential for the IMP in the intended patient population:

- AESIs involving the terms of Behavioral addiction, Drug abuser, Substance abuser, Dependence, Intentional product misuse, Overdose (accidental, intentional, or prescribed), or Drug diversion will be collected and coded as AESIs in the eCRF.
- Cases of noncompliance, protocol violations, participants lost to follow-up, and any other reasons why participants dropped out of the study will be assessed for presence of AESIs.
- Qualitative urine drug test data will be collected prior to each Experimental Session. Any positive findings that cannot be attributed to pre-approved concomitant medications or diet will be reviewed by the Medical Monitor to assess compliance with ongoing eligibility criteria and for presence of AESIs.

If an AESI is a SAE (Serious Adverse Event) or if it involves suicide risk, it should be reported to the sponsor with a narrative via the eCRF within 24 hours of the site's awareness of the event.

11.1.2 Serious Adverse Events

In accordance with guidance for the specific study site, Health Canada Clinical Safety Data Management Definitions and Standards for Expedited Reporting ICH Topic E2A: Guidance for Industry or FDA Safety Reporting Requirements for INDs and BA/BE Studies, an SAE is defined as any untoward medical occurrence that at any dose:

- Results in death.

- Is life-threatening (i.e., the participant was, in the opinion of the investigator, at immediate risk of death from the event as it occurred); it does not refer to an event which hypothetically might have caused death if it were more severe.
- Requires or prolongs inpatient hospitalization.
- Results in persistent or significant disability/incapacity (i.e. the event causes substantial disruption of a person's ability to conduct normal life functions).
- Results in a congenital anomaly/birth defect.
- Requires intervention to prevent permanent impairment or damage.
- Is an important and significant medical event that may not be immediately life-threatening or resulting in death or hospitalization, but based upon appropriate medical judgment, may jeopardize the participant or may require intervention to prevent one of the other outcomes listed above.

AEs which do not fall into these categories are defined as non-serious. It should be noted that a severe Adverse Event need not be serious in nature and that a SAE need not, by definition, be severe.

In addition, a pre-existing event or condition that results in hospitalization should be recorded on the medical history. The hospitalization would not result in the event or condition being reported as a study-related SAE, unless, in the view of the site physician, hospitalization was prolonged as a result of participation in the clinical trial or was necessary due to a worsening of the pre-existing condition. This is because the onset of the event (the reason for the procedure) occurred before the participant was entered in the trial. Hospitalization for cosmetics, non-emergency prophylaxis, or elective abortion does not result in an SAE report, unless, in the view of the site physician, hospitalization for these procedures was prolonged as a result of participation in the clinical trial.

All SAEs will be collected from enrollment through Study Termination. All SAEs which occur during the course of the trial, whether considered to be associated with IMP or not, must be reported to the sponsor within 24 hours of the site staff's awareness of occurrence. Reporting procedures will be provided to the site. All SAEs will be assessed for relationship, expectedness and any required actions to address safety at the time of reporting of the event. SAEs will be evaluated by the site physician and Medical Monitor to determine if it is appropriate for the participant to continue treatment or enter follow-up.

11.2 Other Significant Events

Significant life events that may occur during the course of the study, including death of a loved one, loss of employment, or other hardship, may have an impact on treatment outcome. The sponsor will capture these life events using the LEC-5 measure. Such events will be entered as Comments in the eCRF and if appropriate, described in the Case Study Report for data outliers, if any.

11.3 Pregnancy

11.3.1 Definition of Childbearing Potential

A participant is considered of childbearing potential if they were assigned female at birth and are post-menarche. A participant is considered not of childbearing potential if they are premenarchal, surgically sterile (documented hysterectomy, bilateral salpingectomy, bilateral oophorectomy, and/or tubal ligation), postmenopausal, or assigned male at birth.

11.3.2 Contraception Guidelines

Study participants who were assigned male at birth with partners of childbearing potential will not be required to practice birth control. Adequate birth control methods are required for participants of childbearing potential and include:

- Intrauterine device (IUD)
- Intrauterine hormone-releasing system (IUS)
- Non-oral hormonal methods, including injected, intravaginal, implanted, transdermal
- Oral hormones plus a barrier contraception (condom, diaphragm, or spermicide)
- Double barrier method (at least two of the following: condom, diaphragm, and spermicide)
- Vasectomized sole partner
- Abstinence from penile-vaginal intercourse
    - The reliability of abstinence should be evaluated carefully with the participant in relation to their general lifestyle. An additional acceptable birth control method should be discussed with the participant in case they decide to engage in penile-vaginal intercourse during the course of the study.

For questions about acceptable birth control methods, contact the Medical Monitor.

11.3.3 Follow-up Requirements

Details of all pregnancies in study participants will be collected after Enrollment and collected through 10 days after the last Experimental Session. Pregnancies should be reported to the sponsor via telephone or email within 24 hours of site staff awareness.

In the event of a pregnancy, the participant will discontinue Experimental Sessions but may continue with non-drug Integrative Sessions, the next CAPS-5 assessment, and Study Termination procedures. At a minimum, prior to withdrawal from the study, efforts should be made to assess the final CAPS-5 immediately and complete Study Termination procedures. The participant will be emergency unblinded if the participant wishes as described in Section 9.4 Blinding, after the final CAPS-5 assessment.

The investigator will collect follow-up information on the participant and neonate and forward to the sponsor until the outcome of the pregnancy, which will be reported on an optional Pregnancy eCRF. Any termination, elective or spontaneous, will be reported. Abnormal pregnancy outcomes, such as spontaneous abortion, fetal death, stillbirth, congenital abnormalities, or ectopic pregnancy, will be reported as SAEs.

11.4 Medical Monitor

The Medical Monitor is:

Michael C. Mithoefer, M.D.

Alia Lilienstein, M.D., MPH

12.0 Concomitant Medications

12.1 Tapering Instructions

The site physician will record concomitant medications during Screening. If the prospective participant is being treated with prohibited psychiatric medications at enrollment, the prospective participant will be encouraged to discuss medication tapering with their outside treating physician, if any, and will be required to give the site physician permission to do so as well. The medications will then be tapered in an appropriate fashion to avoid withdrawal effects and will be discontinued at least five half-lives plus one additional week for stabilization before the first Experimental Session to avoid the possibility of any interaction.

The site physician will consult the prescribing physician to initiate medication tapering for participants, as they must refrain from taking psychiatric medications throughout the study, with some exceptions (see Section 12.2 Allowed Concomitant Medications). The prescribing physician's opinion about medication discontinuation will be documented either in writing from the prescribing physician, or in writing by the site physician documenting phone contact with the prescribing physician. Tapering will follow a time course appropriate for the medication based on its half-life, with the Baseline CAPS-5 scheduled to occur after complete washout (five half-lives plus at least 1 week for stabilization).

The therapy team will request information about any changes in medication at each contact. The site physician will be responsible for reviewing and confirming all medications collected during the study.

All medications, non-prescription and prescription, will be collected from Screening through 7 days after the last Experimental Session. From 7 days after the last Experimental Session through Study Termination, only prescription or non-prescription medications taken to treat AEs will be collected. Throughout the protocol, all medications used to treat AEs will be collected, and all changes including discontinuations or additions to medications will be collected. The study team will also inquire about concomitant medication adherence and document all information on the Concomitant Medications eCRF.

Participants may return to taking psychiatric medications and discontinue birth control after the final Study Termination visit if necessary.

12.2 Allowed Concomitant Medications

The site physician may prescribe necessary and appropriate medications in accordance with local and state regulations during the study to treat AEs that do not respond to other management outlined in the Treatment Manual. Examples include concomitant benzodiazepines for uncontrolled anxiety (for example, lorazepam at modest doses and occasional use only to avoid withdrawal effects of discontinuation between Experimental Sessions) or sleep aids (excluding trazodone) in compliance with Section 12.3 Prohibited Medications. Sublingual nitroglycerin will be available on site in the case of emergency.

Gabapentin or certain opiates will be allowed when prescribed for pain management.

All psychoactive medications, herbal supplements, nonprescription medications, and prescription medications must be reviewed by the research team at least 2 weeks prior to each Experimental Session. Failure to comply with protocol requirements for concomitant medications may result in withdrawal from treatment, depending on the investigator and Medical Monitor judgment.

12.3 Prohibited Medications

To be enrolled in the study, participants must:

- Be willing to comply with all medication requirements per protocol. Medications will only be discontinued after enrollment per clinical judgment of the site physician in consultation with the prescribing physician.

Diphenhydramine is excluded from this study unless prior approval is granted by the site physician.

If an SSRI, SNRI, MAOI, or other antidepressant is used between Experimental Session 1 and Study Termination, the participant will discontinue study treatment and continue in follow-up. Participants whose Experimental Sessions are delayed by the COVID-19 pandemic may start any of these medications during the delay, as clinically indicated. When study visits resume, they will be given the option to resume Experimental Sessions after tapering off of these medications, per the tapering plan described above.

13.0 Clinical Laboratory Assessments

The site physician will confirm laboratory assessments gathered in screening for assessing eligibility. The site physician will use a list of normal ranges to conclude whether participants are eligible for the protocol and will indicate justification for admitting participants with abnormal values after consultation with the Medical Monitor.

The following laboratory assessments will be performed as a part of Screening:

- Serum electrolytes and metabolic profile
    - Alanine aminotransferase (ALT)/serum glutamic pyruvic transaminase (SGPT)
    - Albumin:Globulin (A:G) ratio
    - Albumin, serum
    - Alkaline phosphatase, serum
    - Aspartate aminotransferase (AST)/serum glutamic oxaloacetic transaminase (SGOT)
    - Bilirubin, total
    - Blood urea nitrogen (BUN):creatinine ratio
    - Calcium, serum
    - Carbon dioxide
    - Chloride, serum
    - Creatinine, serum
    - C-reactive protein (CRP)
    - Globulin, total
    - Glucose, serum
    - Potassium, serum
    - Protein, total, serum
    - Sodium, serum
- CBC
    - Hematocrit
    - Hemoglobin
    - Mean corpuscular volume (MCV)
    - Mean corpuscular hemoglobin (MCH)
    - Mean corpuscular hemoglobin concentration (MCHC)

    - Red cell distribution width (RDW)
    - Percentage and absolute differential counts
    - Red blood cell (RBC) count
    - White blood cell (WBC) count
- Urinalysis
    - Color
    - Appearance
    - Specific gravity
    - pH
    - Protein
    - Glucose
    - Ketones
    - Occult blood
    - Leukocyte esterase
    - Nitrite
    - Bilirubin
    - Urobilinogen
- Thyroid function
    - Thyroid-stimulating hormone (TSH) high sensitivity (if abnormal, free T3 and T4 will also be tested)
- HCV if indicated
- HIV serology
- %Carbohydrate deficient transferrin (%CDT) to detect heavy alcohol use
- Urine-dip pregnancy test for participants of childbearing potential will be performed at the site
- Urinary drug test will be performed at the site

Laboratory assessments, with the exception of urine pregnancy and drug tests, will be performed at the nearest clinical laboratory to the site. Clinical laboratories for each site will be specified in a separate document. Certificates and normal ranges will be stored in the site's Investigator Site File (ISF).

14.0 Statistical Considerations

Key personnel, MAPS, and the biostatistician will agree on a Statistical Analysis Plan at the beginning of the study, which will provide more detail about analyses than provided in this protocol.

15.0 Study Governance

The sponsor, MAPS, holds the IND for MDMA and is responsible for funding the Clinical Development Program. The sponsor has delegated the primary responsibility of trial organization to MPBC, including designing, initiating, managing, coordinating, continuing, and concluding the clinical trials within the Clinical Development Program. MPBC is tasked with maintaining the quality of study conduct through ongoing monitoring of data and participating in writing study publications. MPBC contracts with independent entities who represent clinical sites to accomplish these goals. Collectively, MAPS and MPBC are referred to as sponsor throughout this document.

15.1 Ethics

This clinical study was designed and shall be implemented and reported in accordance with the International Conference on Harmonisation (ICH) Guidelines for Good Clinical Practice (GCP),

with applicable local regulations (including European Directive 2001/20/EC, US Code of Federal Regulations Title 21) and with the ethical principles laid down in the Declaration of Helsinki.

The protocol and the ICF must be reviewed and approved by a properly constituted institutional review board (IRB) or ethics committee and national regulatory agency (FDA, Health Canada, or Israeli Ministry of Health) before study start. Signed and dated documentation of approvals must be provided to the sponsor. Prior to study start, the investigator is required to sign a signature page confirming their agreement to conduct the study in accordance with these documents and all of the instructions and procedures found in this protocol and to give access to all relevant data and records to the sponsor.

15.1.1 Financial Disclosure

Investigators will adequately and accurately disclose financial interests to the sponsor prior to study start, during the study if financial interests change, and 1 year after study completion. The sponsor will submit necessary disclosures to the appropriate regulatory bodies.

15.1.2 Informed Consent

The investigator and therapy team are responsible for obtaining informed consent in adherence to GCP and according to applicable regulations prior to entering the participant into the trial. Potential participants maybe sent the ICF to review after the initial phone screen. Preferably, informed consent will be obtained by the therapy team that will treat the participant. Information about the study must be given orally and in an understandable written ICF. The informed consent discussion must be conducted by a person who is qualified according to federal, state, or local regulations. The participant should have the opportunity to inquire about details of the study and to consider participation.

The therapy team may meet with the potential participant via telemedicine for ICF review and signing prior to in person screening if necessary for scheduling of screening activities. If this is completed by telemedicine visit, the team will ensure the ICF is thoroughly explained and reviewed just as it would be at an in-person visit. If the potential participant is still interested after review, they will sign the consent during that telemedicine visit. The participant will then bring their signed copy of the ICF to their next in person visit where study staff will then counter sign the ICF, copy the ICF for the participant and file the original at the site.

In addition to the explanation of study visits, the information should include that access to original medical records and processing of coded personal information must be authorized. A written release is needed to give permission to site staff to request and view the participant's medical records to assess protocol eligibility, if needed. Information necessary for protocol participation includes past medical history, psychiatric interview, physical examination, and clinical laboratory tests.

Eligible participants may only be included in the study after signing the IRB approved ICF. Informed consent must be obtained before conducting any study-specific procedures (i.e., all of the procedures described in the protocol beyond phone screening). The process of obtaining informed consent should be documented in the participant's source records. The study staff will provide a copy of the signed ICF to the participant and will maintain the original in the ISF.

The written ICF and any other written information to be provided to participants should be revised whenever important new information becomes available that may be relevant to the participant's consent. Any revised ICF and written information should receive approval from an

IRB before use. The participant should be informed in a timely manner if new information becomes available that may affect the decision to take part or continue in the study. The communication of this information should be documented. Participants can withdraw consent at any time without prejudice. If a participant withdraws consent but does not revoke the Health Insurance Portability and Accountability Act (HIPAA) authorization, the study team will have full access to their medical records, including Study Termination visit information. If a participant revokes only the HIPAA authorization, the study team will have full access to all medical records prior to the date and time of revocation.

If a participant fails Screening and is rescreened at a later date, a new copy of the ICF should be signed.

15.2 Study Monitoring, Auditing, and Documentation

Investigators, therapy teams, and all study staff will be trained prior study start for each site. Study sites will be monitored by site visits and telephone calls by representatives of the sponsor. In addition, critical data and systemic issues will be subject to centralized monitoring via the EDC system to develop and evaluate strategies for correction across sites. Sites will be monitored as appropriate for the rate of enrollment to comply with GCP guidelines and to ensure validity of study data. During each monitoring visit, source data verification will be performed to ensure compliance, including accurate and complete recording of data on eCRFs, source records, and IMP accountability records. An eCRF collation will be completed for each participant enrolled within the EDC system.

CAPS-5 results will not be sent to site staff but instead will be sent to the IR Coordinator. The IR Coordinator will be responsible for review and data entry of the CAPS-5 source records into CAPS-5 eCRFs which will be blinded to sponsor and site personnel during the study.

Videos from selected sessions will be reviewed for adherence to the Treatment Manual as described in Section 6.4. Findings from video reviews may be discussed with therapy teams as needed to ensure continued adherence to the protocol.

During or after the study, the regulatory authorities, the IRB, and/or representatives of the sponsor may request access to all source documents, eCRFs, and other protocol documentation for on-site audit or inspection. Monitoring and auditing procedures will be supplied in a separate document.

15.2.1 Source Records

Source records contain all primary evidence of existence of the participant and document all study procedures. Source records include but are not limited to medical records, measures, checklists, notes, emails, and laboratory reports. All data reported in the eCRF are transcribed from primary source documents and must be consistent. These documents are maintained at the study site securely. Source records of CAPS-5 assessments will be stored in dedicated limited access files during the study.

15.3 Confidentiality

Every effort will be made to strictly safeguard the confidentiality of participants. Despite this, privacy cannot be guaranteed. Removing identifying information from data and restricting access to researchers directly involved in assessing the participants should prevent the dissemination of confidential data. Except for the Screening Log, the Informed Consent, previous medical records,

emails with the participant, and a Contact Information Sheet that will be stored separately from other documents, all source data will be identified only by the participant's initials and SUBJID. If past medical records are needed, participants will sign forms for the release of information upon consent to permit screening for protocol enrollment. In accordance with the guidance for a specific study site location, Health Canada Guidance for Records Related to Clinical Trials (GUIDE-0068) or FDA E6(R2) Good Clinical Practice: Integrated Addendum to ICH E6(R1), all assessment records will be kept in a locked file drawer or cabinet in a locked office, and access to measures will be limited to regulatory agencies, researchers, and individuals analyzing data. Researchers, other than the investigators who are directly involved in the protocol, with access to data will not be provided with any information that would identify participants by name or by other means, such as social security number.

Maintaining data in a secure environment will prevent the accidental or deliberate examination or removal of data. The sponsor will utilize confidentiality procedures to assure participant privacy. Audiovisual recordings are necessary for sponsor oversight of therapy processes. Any requests for use of audiovisual recordings outside of research and training requests will result in participants receiving information on the request. Participants will have control over any presentation of audiovisual recordings beyond viewing by authorized researchers, sponsor staff, or regulatory agencies. The sponsor uses encrypted, secure technology to transfer and store recordings, but there is always a risk of a security breach. The sponsor is committed to taking preventative measures to avoid such an event. In the case of a security breach, the participant will be notified, and all efforts will be made to minimize the dissemination of recorded content.

Clinical trial data other than video data will be hosted on an EDC system that is FDA-compliant. All data entered into this system will be de-identified. Participants will only be referred to by numbers and a secondary identifier code. Source Records and identifying information will be retained at clinical sites per GCP. The sponsor will train the study staff on EDC procedures. Each study staff member with access to the data will be given an individual password.

The sponsor has developed a feature that will allow participants to create a password and enter their self-report questionnaire data directly into Medrio using the electronic Participant Reported Outcome (ePRO) feature. Participants will be reminded by email to enter the data. Participant emails will be treated as Protected Health Information (PHI) in the database. Participants will receive a welcome email and reminder emails to ensure that they provide all necessary data.

15.4 Costs to Participants

There will be no costs to the study participants for participation. The sponsor will cover all direct costs of study procedures required for participation, including any assessments or tests performed solely for the purpose of establishing eligibility for participation. Charges for treatment of a participant's condition that are unrelated to the research study or any unrelated procedures will not be covered by the sponsor. Patients who previously received therapy from a therapy team member prior to the study, and who will continue to receive ongoing treatment outside of the study from that therapist, are responsible for those non-study related costs. Participants may be reimbursed for reasonable expenses incurred for study participation, such as local travel to the treatment site; this will be specified in each site's consent.

15.5 Treatment and Compensation for Study Related Injury

Some study-related emergencies can be treated by the site physicians. If the site physicians cannot treat a study-related emergency, then there are contingency plans for the transport of participants to the nearest hospital. Treatment of a study-related emergency would first be billed

to a participant's health insurance provider. If the participant's private or employer health insurance plan does not cover clinical trial-related claims, then the sponsor will cover any treatment costs directly related to the study. The sponsor will not cover costs of ongoing treatment unrelated to the study due to pre-existing conditions, or the cost of the participant's time spent obtaining treatment for pre-existing conditions before receiving treatment in the study. In the event of a suit against the sponsor, the sponsor carries third-party insurance that will cover bodily injury claims and will pay for applicable legal defense if needed/warranted.

15.6 Record Retention

Investigators must retain all study records required by the sponsor and applicable ICH-GCP and FDA regulations, and Health Canada Guidance for Records Related to Clinical Trials (GUIDE-0068) in a secure and safe facility. The investigator must consult a representative of the sponsor before disposal of any study records. "Essential documents" are defined as documents that individually and collectively permit evaluation of the conduct of a trial and the quality of the data produced. These documents will be filed according to ICH-GCP regulations in the ISF. It is the responsibility of the sponsor to inform the investigator or institution when these documents no longer need to be retained.

15.7 Publication Policy

The sponsor recognizes the importance of communicating medical research and scientific data and their obligations to participants enrolled in a study and therefore, encourage publication of such material in reputable scientific journals and at professional and/or academic seminars or conferences. For multi-center studies, it is intended that the first publication of the study's primary clinical data be co-authored by designated participating centers and the sponsor or designated representatives. Inclusion of Clinical Investigators in the authorship of any multi-center publication will be based upon substantial contribution to the design, analysis, interpretation of data, drafting and/or critically revising any manuscript(s) derived from the Study. All publications will follow ICMJE Recommendations for the Conduct, Reporting, Editing, and Publication of Scholarly Work in Medical Journals, unless other guidelines are required by the journal. It is understood by the Clinical Investigators that the information generated in this study will be used by the sponsor in connection with the development of the IMP and therefore may be disclosed to government agencies in various countries. To allow for the use of information derived from the study, it is understood that the investigators are obliged to provide the sponsor with complete test results, all study data, and access to all study records. It is mandatory that all data analysis is done on the official monitored sponsor database and that the analysis plan is agreed upon with the sponsor statistician.

Any results of medical investigations with the sponsor and/or publication/lecture/manuscripts based thereon shall be exchanged and discussed by the investigator and sponsor prior to submission for publication or presentation. Due regard shall be given to the sponsor's legitimate interests, e.g., manuscript authorship, obtaining optimal patient protection, coordinating and maintaining submissions to health authorities, and coordinating with other ongoing studies in the same field. The full details of the processes of producing and reviewing reports, manuscripts, and presentations based on the data from this trial will be described in the Clinical Trial Agreement.

16.0 References

1. Lanius, R.A., et al., *The dissociative subtype of posttraumatic stress disorder: rationale, clinical and neurobiological evidence, and implications.* Depress Anxiety, 2012. **29**(8): p. 701-8.
2. Lanius, R.A., et al., *Emotion modulation in PTSD: Clinical and neurobiological evidence for a dissociative subtype.* Am J Psychiatry, 2010. **167**(6): p. 640-7.
3. Nicholson, A.A., et al., *The Dissociative Subtype of Posttraumatic Stress Disorder: Unique Resting-State Functional Connectivity of Basolateral and Centromedial Amygdala Complexes.* Neuropsychopharmacology, 2015. **40**(10): p. 2317-26.
4. Hoge, C.W., et al., *Combat duty in Iraq and Afghanistan, mental health problems, and barriers to care.* N Engl J Med, 2004. **351**(1): p. 13-22.
5. *Statement Of Jon A. Wooditch Acting Inspector General Department Of Veterans Affairs*, in *Committee On Veterans' Affairs Subcommittee On Disability Assistance And Memorial Affairs*. 2005: Washington, DC.
6. Solon, O., *My therapist gave me a pill: Can MDMA help cure trauma? https://www.theguardian.com/society/2016/sep/16/mdma-ptsd-therapy-trauma-maps-medical-study#comments*, in *The Guardian*. 2016.
7. Kemp, J. and R. Bossarte, *Suicide Data Report 2012*. 2013, US Department Of Veterans Affairs, Mental Health Services, Suicide Prevention Program http://www.va.gov/opa/docs/Suicide-Data-Report-2012-final.pdf%20.
8. Brady, K., et al., *Efficacy and safety of sertraline treatment of posttraumatic stress disorder: a randomized controlled trial.* JAMA, 2000. **283**(14): p. 1837-44.
9. Davidson, J.R., et al., *Multicenter, double-blind comparison of sertraline and placebo in the treatment of posttraumatic stress disorder.* Arch Gen Psychiatry, 2001. **58**(5): p. 485-92.
10. Friedman, M.J., et al., *Randomized, double-blind comparison of sertraline and placebo for posttraumatic stress disorder in a Department of Veterans Affairs setting.* J Clin Psychiatry, 2007. **68**(5): p. 711-20.
11. Arikian, S.R. and J.M. Gorman, *A Review of the Diagnosis, Pharmacologic Treatment, and Economic Aspects of Anxiety Disorders.* Prim Care Companion J Clin Psychiatry, 2001. **3**(3): p. 110-117.
12. Sidran Institute. *Post traumatic stress disorder fact sheet*. 2016; Available from: https://www.sidran.org/resources/for-survivors-and-loved-ones/post-traumatic-stress-disorder-fact-sheet/ Accessed September 2016.
13. Ursano, R.J., et al., *Practice guideline for the treatment of patients with acute stress disorder and posttraumatic stress disorder.* Am J Psychiatry, 2004. **161**(11 Suppl): p. 3-31.
14. Foa, E.B., et al., *Effective Treatments for PTSD, Practice Guidelines from the International Society for Traumatic Stress Studies*. Second ed. 2009, New York, NY: Guilford Press.
15. Benish, S.G., Z.E. Imel, and B.E. Wampold, *The relative efficacy of bona fide psychotherapies for treating post-traumatic stress disorder: a meta-analysis of direct comparisons.* Clin Psychol Rev, 2008. **28**(5): p. 746-58.
16. Cukor, J., et al., *Emerging treatments for PTSD.* Clin Psychol Rev, 2009. **29**(8): p. 715-26.
17. Basoglu, M., E. Salcioglu, and M. Livanou, *A randomized controlled study of single-session behavioural treatment of earthquake-related post-traumatic stress disorder using an earthquake simulator.* Psychol Med, 2007. **37**(2): p. 203-13.
18. Gerardi, M., et al., *Virtual reality exposure therapy using a virtual Iraq: case report.* J Trauma Stress, 2008. **21**(2): p. 209-13.

19. Heresco-Levy, U., et al., *Pilot-controlled trial of D-cycloserine for the treatment of post-traumatic stress disorder.* Int J Neuropsychopharmacol, 2002. **5**(4): p. 301-7.
20. Freudenmann, R.W., F. Oxler, and S. Bernschneider-Reif, *The origin of MDMA (ecstasy) revisited: the true story reconstructed from the original documents.* Addiction, 2006. **101**(9): p. 1241-5.
21. Shulgin, A.T., *The background and chemistry of MDMA.* J Psychoactive Drugs, 1986. **18**(4): p. 291-304.
22. Farre, M., et al., *Pharmacological interaction between 3,4-methylenedioxymethamphetamine (ecstasy) and paroxetine: pharmacological effects and pharmacokinetics.* J Pharmacol Exp Ther, 2007. **323**(3): p. 954-62.
23. Liechti, M.E. and F.X. Vollenweider, *The serotonin uptake inhibitor citalopram reduces acute cardiovascular and vegetative effects of 3,4-methylenedioxymethamphetamine ('Ecstasy') in healthy volunteers.* J Psychopharmacol, 2000. **14**(3): p. 269-74.
24. Liechti, M.E. and F.X. Vollenweider, *Which neuroreceptors mediate the subjective effects of MDMA in humans? A summary of mechanistic studies.* Hum Psychopharmacol, 2001. **16**(8): p. 589-598.
25. Tancer, M. and C.E. Johanson, *The effects of fluoxetine on the subjective and physiological effects of 3,4-methylenedioxymethamphetamine (MDMA) in humans.* Psychopharmacology (Berl), 2007. **189**(4): p. 565-73.
26. Hysek, C.M., et al., *The norepinephrine transporter inhibitor reboxetine reduces stimulant effects of MDMA ("ecstasy") in humans.* Clin Pharmacol Ther, 2011. **90**(2): p. 246-55.
27. Hysek, C.M., et al., *Pharmacokinetic and pharmacodynamic effects of methylphenidate and MDMA administered alone or in combination.* Int J Neuropsychopharmacol, 2014. **17**(3): p. 371-81.
28. Liechti, M.E. and F.X. Vollenweider, *Acute psychological and physiological effects of MDMA ("Ecstasy") after haloperidol pretreatment in healthy humans.* Eur Neuropsychopharmacol, 2000. **10**(4): p. 289-95.
29. Huizink, A.C., et al., *Symptoms of anxiety and depression in childhood and use of MDMA: prospective, population based study.* Bmj, 2006. **332**(7545): p. 825-8.
30. Lieb, R., et al., *Mental disorders in ecstasy users: a prospective-longitudinal investigation.* Drug Alcohol Depend, 2002. **68**(2): p. 195-207.
31. von Sydow, K., et al., *Use, abuse and dependence of ecstasy and related drugs in adolescents and young adults-a transient phenomenon? Results from a longitudinal community study.* Drug Alcohol Depend, 2002. **66**(2): p. 147-59.
32. Foa, E.B., *Prolonged exposure therapy for PTSD: emotional processing of traumatic experiences: therapist guide*. 2007, New York: Oxford University Press.
33. Wilbarger, P. and J. Wilbarger, *Sensory defensiveness and related social/emotional and neurological problems*. 1997, Van Nuys, CA.: Avanti Education Program.
34. Siegel, D.J., *The Developing Mind*. 1999, New York: Guilford Press.
35. Ogden, P., K. Minton, and C. Pain, *Trauma and the body: A sensorimotor approach to psychotherapy (norton series on interpersonal neurobiology)*. 2006: WW Norton & Company.
36. Bedi, G., et al., *Effects of MDMA on sociability and neural response to social threat and social reward.* Psychopharmacology (Berl), 2009. **207**(1): p. 73-83.
37. Gamma, A., et al., *3,4-Methylenedioxymethamphetamine (MDMA) modulates cortical and limbic brain activity as measured by [H(2)(15)O]-PET in healthy humans.* Neuropsychopharmacology, 2000. **23**(4): p. 388-95.
38. Rasmusson, A.M. and D.S. Charney, *Animal models of relevance to PTSD.* Ann N Y Acad Sci, 1997. **821**: p. 332-51.

39. Davis, M. and C. Shi, *The extended amygdala: are the central nucleus of the amygdala and the bed nucleus of the stria terminalis differentially involved in fear versus anxiety?* Ann N Y Acad Sci, 1999. **877**: p. 281-91.
40. Phelps, E.A., et al., *Activation of the left amygdala to a cognitive representation of fear.* Nat Neurosci, 2001. **4**(4): p. 437-41.
41. Metzner, R. and S. Adamson, *Using MDMA in healing, psychotherapy and spiritual practice*, in *Ecstasy, A Complete Guide: A Comprehensive Look at the Risks and Benefits of MDMA.*, J. Holland, Editor. 2001, Inner Traditions: Rochester VT. p. 182-207.
42. Stolaroff, M., *The Secret Chief Revealed: Conversations with a pioneer of the underground therapy movement.* 2004, Sarasota FL: Multidisciplinary Association for Psychedelic Studies.
43. Mithoefer, M.C., et al., *The safety and efficacy of {+/-}3,4-methylenedioxymethamphetamine-assisted psychotherapy in subjects with chronic, treatment-resistant posttraumatic stress disorder: the first randomized controlled pilot study*. J Psychopharmacol, 2011. **25**(4): p. 439-52.
44. Bouso, J.C., et al., *MDMA-assisted psychotherapy using low doses in a small sample of women with chronic posttraumatic stress disorder.* J Psychoactive Drugs, 2008. **40**(3): p. 225-36.
45. Greer, G.R. and R. Tolbert, *A method of conducting therapeutic sessions with MDMA.* J Psychoactive Drugs, 1998. **30**(4): p. 371-379.
46. Shulgin, A.T. and D.E. Nichols, *Characterization of three new psychotomimetics*, in *The Pharmacology of Hallucinogens*, R.C. Stillman and R.E. Willette, Editors. 1978, Pergamon: New York.
47. Anderson, G.M.d., et al., *Absolute configuration and psychotomimetic activity.* NIDA Res Monogr, 1978. **22**: p. 8-15.
48. Cami, J., et al., *Human pharmacology of 3,4-methylenedioxymethamphetamine ("ecstasy"): psychomotor performance and subjective effects.* J Clin Psychopharmacol, 2000. **20**(4): p. 455-66.
49. Harris, D.S., et al., *Subjective and hormonal effects of 3,4-methylenedioxymethamphetamine (MDMA) in humans.* Psychopharmacology (Berl), 2002. **162**(4): p. 396-405.
50. Tancer, M. and C.E. Johanson, *Reinforcing, subjective, and physiological effects of MDMA in humans: a comparison with d-amphetamine and mCPP*. Drug Alcohol Depend, 2003. **72**(1): p. 33-44.
51. Vollenweider, F.X., et al., *Psychological and cardiovascular effects and short-term sequelae of MDMA ("ecstasy") in MDMA-naive healthy volunteers.* Neuropsychopharmacology, 1998. **19**(4): p. 241-51.
52. Grinspoon, L. and J.B. Bakalar, *Can drugs be used to enhance the psychotherapeutic process?* Am J Psychother, 1986. **40**(3): p. 393-404.
53. Adamson, S., *Through the gateway of the heart: Accounts of experiences With MDMA and other empathogenic substances*. 1985, San Francisco CA: Four Trees Publications.
54. Shulgin, A. and A. Shulgin, *Pihkal: A Chemical Love Story*. 1st ed. 1991, Berkeley, CA: Transform Press. 1-978.
55. Downing, J., *The psychological and physiological effects of MDMA on normal volunteers.* J Psychoactive Drugs, 1986. **18**(4): p. 335-40.
56. Greer, G. and R. Tolbert, *Subjective reports of the effects of MDMA in a clinical setting.* J Psychoactive Drugs, 1986. **18**(4): p. 319-27.
57. Grob, C.S., et al., *Psychobiologic effects of 3,4-methylenedioxymethamphetamine in humans: methodological considerations and preliminary observations.* Behav Brain Res, 1996. **73**(1-2): p. 103-7.
58. Grob, C., *MDMA research: preliminary investigations with human subjects.* Int J Drug Policy, 1998. **9**(2): p. 119-124.

59. Mithoefer, M.C., et al., *Durability of improvement in post-traumatic stress disorder symptoms and absence of harmful effects or drug dependency after 3,4-methylenedioxymethamphetamine-assisted psychotherapy: a prospective long-term follow-up study*. J Psychopharmacol, 2013. **27**(1): p. 28-39.
60. Oehen, P., et al., *A randomized, controlled pilot study of MDMA (+/- 3,4-Methylenedioxymethamphetamine)-assisted psychotherapy for treatment of resistant, chronic Post-Traumatic Stress Disorder (PTSD).* J Psychopharmacol, 2013. **27**(1): p. 40-52.
61. Chabrol, H. and P. Oehen, *MDMA assisted psychotherapy found to have a large effect for chronic post-traumatic stress disorder.* J Psychopharmacol, 2013. **27**(9): p. 865-6.
62. Weathers, F.W., et al., *The Clinician-Administered PTSD Scale for DSM-5 (CAPS-5)*. 2013, Washington DC: National Center for PTSD.
63. Sheehan, D., *The Anxiety Disease*. 1983, New York, NY: Scribner's.
64. Leon, A.C., et al., *Assessing psychiatric impairment in primary care with the Sheehan Disability Scale.* Int J Psychiatry Med, 1997. **27**(2): p. 93-105.
65. Posner, K., et al., *Columbia Classification Algorithm of Suicide Assessment (C-CASA): classification of suicidal events in the FDA's pediatric suicidal risk analysis of antidepressants.* Am J Psychiatry, 2007. **164**(7): p. 1035-43.
66. Posner, K., et al., *The Columbia-Suicide Severity Rating Scale: initial validity and internal consistency findings from three multisite studies with adolescents and adults.* Am J Psychiatry, 2011. **168**(12): p. 1266-77.
67. Sheehan, D.V., et al., *The Mini-International Neuropsychiatric Interview (M.I.N.I.): the development and validation of a structured diagnostic psychiatric interview for DSM-IV and ICD-10.* J Clin Psychiatry, 1998. **59 Suppl 20**: p. 22-33;quiz 34-57.
68. Lecrubier, Y., et al., *The Mini International Neuropsychiatric Interview (MINI). A short diagnostic structured interview: reliability and validity according to the CIDI.* Eur Psychiatry. **12**(5).
69. Sheehan, D.V., et al., *The validity of the Mini International Neuropsychiatric Interview (MINI) according to the SCID-P and its reliability*. Eur Psychiatry, 1997. **12**(5): p. 232-241.
70. First, M.B., et al., *User’s Guide for the SCID-5-PD (Structured Clinical Interview for DSM-5 Personality Disorder).* SCID 5 2015, Arlington, VA: American Psychiatric Association.
71. Weathers, F.W., et al., eds. *The Life Events Checklist for DSM-5 (LEC-5). Instrument obtainable from National Center for PTSD www.ptsd.va.gov*. 2013, National Center for PTSD: www.ptsd.va.gov.
72. Weathers, F.W., Litz, B.T., , et al. *The PTSD Checklist for DSM-5 (PCL-5). Available at www.ptsd.va.gov*. 2013.
73. Ross, C.A., et al., *Differentiating Multiple Personality Disorder and Dissociative Disorder Not Otherwise Specified.* Dissociation, 1992. **5**(2): p. 87-90.
74. Eidhof, M.B., et al., *Dissociative Subtype of PTSD Interview (DSP-I) FOR DSM-5*. 2017, self-published by authors.
75. Steuwe, C., R.A. Lanius, and P.A. Frewen, *Evidence for a dissociative subtype of PTSD by latent profile and confirmatory factor analyses in a civilian sample.* Depress Anxiety, 2012. **29**(8): p. 689-700.
76. Wolf, E.J., et al., *The dissociative subtype of PTSD: a replication and extension.* Depress Anxiety, 2012. **29**(8): p. 679-88.
77. Wolf, E.J., et al., *A latent class analysis of dissociation and posttraumatic stress disorder: evidence for a dissociative subtype.* Arch Gen Psychiatry, 2012. **69**(7): p. 698-705.

78. Felitti, V.J., et al., *Relationship of childhood abuse and household dysfunction to many of the leading causes of death in adults. The Adverse Childhood Experiences (ACE) Study.* Am J Prev Med, 1998. **14**(4): p. 245-58.
79. Anda, R.F., et al., *Childhood Abuse, Household Dysfunction, and Indicators of Impaired Adult Worker Performance.* Perm J, 2004. **8**(1): p. 30-8.
80. Dong, M., et al., *Insights into causal pathways for ischemic heart disease: adverse childhood experiences study.* Circulation, 2004. **110**(13): p. 1761-6.
81. Dube, S.R., et al., *The impact of adverse childhood experiences on health problems: evidence from four birth cohorts dating back to 1900.* Prev Med, 2003. **37**(3): p. 268-77.
82. McBeth, J., et al., *Associations between adverse events in childhood and chronic widespread pain in adulthood: are they explained by differential recall?* J Rheumatol, 2001. **28**(10): p. 2305-9.
83. Beck, A.T. and C.H. Ward, *Dreams of depressed patients. Characteristic themes in manifest content.* Arch Gen Psychiatry, 1961. **5**: p. 462-7.
84. Beck, A.T. and R.A. Steer, *Internal consistencies of the original and revised Beck Depression Inventory.* J Clin Psychol, 1984. **40**(6): p. 1365-7.
85. Beck, A.T., et al., *Comparison of Beck Depression Inventories -IA and -II in psychiatric outpatients.* J Pers Assess, 1996. **67**(3): p. 588-97.
86. Storch, E.A., J.W. Roberti, and D.A. Roth, *Factor structure, concurrent validity, and internal consistency of the Beck Depression Inventory-Second Edition in a sample of college students.* Depress Anxiety, 2004. **19**(3): p. 187-9.
87. Dixon, D., B. Pollard, and M. Johnston, *What does the chronic pain grade questionnaire measure?* Pain, 2007. **130**(3): p. 249-53.
88. Smith, B.H., et al., *The Chronic Pain Grade questionnaire: validation and reliability in postal research.* Pain, 1997. **71**(2): p. 141-7.
89. van Reenen, M. and B. Janssen, *EQ-5D-5L User Guide: Basic information on how to use the EQ-5D-5L instrument*. 2015.
90. Herdman, M., et al., *Development and preliminary testing of the new five-level version of EQ-5D (EQ-5D-5L).* Qual Life Res, 2011. **20**(10): p. 1727-36.
91. EuroQol, G., *EuroQol--a new facility for the measurement of health-related quality of life.* Health Policy, 1990. **16**(3): p. 199-208.
92. Golicki, D., et al., *Comparing responsiveness of the EQ-5D-5L, EQ-5D-3L and EQ VAS in stroke patients.* Qual Life Res, 2015. **24**(6): p. 1555-63.
93. Briere, J. and M. Runtz, *The Inventory of Altered Self-Capacities (IASC): a standardized measure of identity, affect regulation, and relationship disturbance.* Assessment, 2002. **9**(3): p. 230-9.
94. Marx, B.P. and E. al., *Development of a functional evaluation scale for active duty service members and veterans*, in *International Society for Traumatic Stress Studies*. 2009: Atlanta, GA.
95. Rodriguez, P., D.W. Holowka, and B.P. Marx, *Assessment of posttraumatic stress disorder-related functional impairment: a review.* J Rehabil Res Dev, 2012. **49**(5): p. 649-65.
96. Neff, K., *The Development and validation of a scale to measure self-compassion.* Self and Identity, 2003. **2**: p. 223-250.
97. Neff, K.D. and R. Vonk, *Self-compassion versus global self-esteem: two different ways of relating to oneself.* J Pers, 2009. **77**(1): p. 23-50.
98. Bagby, R.M., J.D. Parker, and G.J. Taylor, *The twenty-item Toronto Alexithymia Scale--I. Item selection and cross-validation of the factor structure.* J Psychosom Res, 1994. **38**(1): p. 23-32.
99. Bagby, R.M., G.J. Taylor, and J.D. Parker, *The Twenty-item Toronto Alexithymia Scale--II. Convergent, discriminant, and concurrent validity.* J Psychosom Res, 1994. **38**(1): p. 33-40.

100. Saunders, J.B., et al., *Development of the Alcohol Use Disorders Identification Test (AUDIT): WHO Collaborative Project on Early Detection of Persons with Harmful Alcohol Consumption--II.* Addiction, 1993. **88**(6): p. 791-804.
101. Allen, J.P., et al., *A review of research on the Alcohol Use Disorders Identification Test (AUDIT).* Alcohol Clin Exp Res, 1997. **21**(4): p. 613-9.
102. Berman, A.H., et al., *Evaluation of the Drug Use Disorders Identification Test (DUDIT) in criminal justice and detoxification settings and in a Swedish population sample.* Eur Addict Res, 2005. **11**(1): p. 22-31.
103. Koslowsky, M., et al., *The factor structure and criterion validity of the short form of the Eating Attitudes Test.* 1992(0022-3891 (Print)).
104. Kessler, R.C., et al., *Using the World Health Organization Health and Work Performance Questionnaire (HPQ) to evaluate the indirect workplace costs of illness.* J Occup Environ Med, 2004. **46**(6 Suppl): p. S23-37.
105. Kessler, R.C., et al., *The World Health Organization Health and Work Performance Questionnaire (HPQ).* J Occup Environ Med, 2003. **45**(2): p. 156-74.
106. Mithoefer, M., *A Manual for MDMA-Assisted Psychotherapy in the Treatment of Posttraumatic Stress Disorder; Version 8*. 2016: http://www.maps.org/research/mdma/mdma-research-timeline/4887-a-manual-for-mdma-assisted-psychotherapy-in-the-treatment-of-ptsd.
107. *Practice advisory: thrombolytic therapy for acute ischemic stroke--summary statement. Report of the Quality Standards Subcommittee of the American Academy of Neurology*. Neurology, 1996. **47**(3): p. 835-9.
108. Adams, H.P., Jr., et al., *Guidelines for the early management of adults with ischemic stroke: a guideline from the American Heart Association/American Stroke Association Stroke Council, Clinical Cardiology Council, Cardiovascular Radiology and Intervention Council, and the Atherosclerotic Peripheral Vascular Disease and Quality of Care Outcomes in Research Interdisciplinary Working Groups: the American Academy of Neurology affirms the value of this guideline as an educational tool for neurologists.* Stroke, 2007. **38**(5): p. 1655-711.
109. Ryan, T.J., et al., *1999 update: ACC/AHA guidelines for the management of patients with acute myocardial infarction. A report of the American College of Cardiology/American Heart Association Task Force on Practice Guidelines (Committee on Management of Acute Myocardial Infarction).* J Am Coll Cardiol, 1999. **34**(3): p. 890-911.
110. Liechti, M.E., A. Gamma, and F.X. Vollenweider, *Gender differences in the subjective effects of MDMA.* Psychopharmacology (Berl), 2001. **154**(2): p. 161-8.
111. McElhatton, P.R., et al., *Congenital anomalies after prenatal ecstasy exposure [letter].* Lancet, 1999. **354**(9188): p. 1441-2.
112. Bateman, D.N., et al., *A case control study to examine the pharmacological factors underlying ventricular septal defects in the North of England.* Eur J Clin Pharmacol, 2004. **60**(9): p. 635-41.
113. McCann, U.D. and G.A. Ricaurte, *Caveat emptor: editors beware.* Neuropsychopharmacology, 2001. **24**(3): p. 333-6.
114. Rogers, G., et al., *The harmful health effects of recreational ecstasy: a systematic review of observational evidence.* Health Technol Assess, 2009. **13**(6): p. iii-iv, ix-xii, 1-315.

Appendix A: Sub-study without Overnight Stays

Rationale

This sub-study to the MAPP1 protocol "A Randomized, Double-Blind, Placebo-Controlled, Multi-Site Phase 3 Study of the Efficacy and Safety of Manualized MDMA-Assisted Psychotherapy for the Treatment of Severe Posttraumatic Stress Disorder" allows participants to be discharged in the evening after the Experimental Session is over at two investigational study sites. In MAPS' Phase 2 study MAA1, which tested the safety and efficacy of MDMA-assisted psychotherapy for social anxiety among autistic adults, an overnight stay was not required. There were no safety concerns due to travel to home or hotel, and this approach was found to be feasible.

Study site and participants will follow all protocol procedures described in the main protocol, except those related to overnight stays as described below. The overnight stay is included in the main study protocol primarily as an opportunity for rest and integration in a relaxed and comfortable environment away from the distractions of home, and to facilitate the logistics of participation in the integrative visit at the site on the day following the Experimental Session. The overnight stay is not required for medical reasons, but in case of emergencies, the therapists and study physician are on-call for all sites.

In the main study protocol, a Night Attendant accompanies the participant during the overnight stay. The Night Attendant's primary function is to ensure the participant is comfortable and has a meal, to provide minimal support, and to alert qualified site staff in case of need expressed by the participant or determined by observation. All Night Attendants are interviewed, trained, and approved by therapy teams to ensure they are comfortable providing the necessary support and know whom to call if medical support is needed from the therapy team or study physician.

Medically-trained Night Attendants have not been required for any of the five Phase 2 studies of MDMA-assisted psychotherapy for PTSD since MP-1 was completed. In MP-1, the first Phase 2 study, the FDA required that the Night Attendant be a registered nurse. Since MP-1, Night Attendants were not required to have medical training in subsequent studies. Among 189 participants treated under this IND, no medical complications have arisen during overnight stays under the watch of Night Attendants.

The sponsor and investigators agree that allowing participants who are screened for stable domestic circumstances to return home will not increase the risks of participation in the study. The study protocol discusses the risk of psychological distress in a PTSD population and mitigation in Section 10.2.2.2. Therapists and site physicians are aware of these risks, including anxiety and depressed mood occasionally reported 1 to 3 days after MDMA administration. In previous Phase 1 and 2 studies, these reactions have been transient and have responded well to reassurance from the therapy team, with occasional use of benzodiazepines for anxiety. If needed, the study physician may prescribe a short-acting, low-dose benzodiazepine (specifically, lorazepam) or sleep aids (excluding trazodone) as needed.

Consistent with the main study protocol and the sub-study, proper preparation and follow-up support will reduce the difficulties participants might have with acute or sub-acute reactions. The potential for destabilizing psychological distress will be minimized by:

- Excluding people who might be more vulnerable to it
- Excluding people who do not have a stable living situation or supportive family/network
- Preparatory Sessions of non-drug psychotherapy before the Experimental Session

- Creating an atmosphere of trust during the Experimental Session
- Close monitoring
- Phone contact with participants during the week after the Experimental Session
- Conduct of Integrative Session on the morning after the Experimental Session

Specific to sub-study:

- Therapist phone contact with participants on the night of the Experimental Session.
- A support person identified by the participant and agreed upon by the therapist must stay with the participant overnight on the evening following the Experimental Session.
- The therapists will meet with the support person prior to the first Experimental Session to assess their ability to act as an appropriate support person and to give them instructions about what to expect following Experimental Sessions and how to contact study personnel if needed.
- A support person must assist the participant during transfer from study site after the Experimental Session and to the study site on the following morning for the Integrative Session. This Integrative Session will start later in the day to allow for logistics of transportation. Participants can arrange their own transportation after the Integrative Session.

Implementation of Sub-study

All eligibility criteria from the main study protocol will apply, except for the following inclusion criteria listed as a Lifestyle Modification:

Post Experimental Session

- Are willing to remain overnight at the study site after each Experimental Session until after the Integrative Session the next morning
- Are willing to be driven home on the morning after the Experimental Sessions after the Integrative Session, either by a driver arranged by the participant, site personnel or taxi.

Sub-study participants will be required to be willing to:

Post Experimental Session

- Identify a support person willing to accompany them overnight at a safe location of their choice after each Experimental Session.
- Have the support person escort them away from the study site in the evening after the Experimental Sessions and to the site on the following morning for the Integrative Session.

Screening & Preparatory Periods

During the Screening and Preparatory Period, the participant will identify an appropriate support person to stay with the participant on the evening of the Experimental Session. Participants will be escorted to and from the study site after Experimental Sessions. The support person is not required to be the same as the escort for the participant. There may be more than one support person(s), and they will be responsible for providing companionship as needed overnight and contacting the study team with any questions or concerns. Any support person spending the night with the participant will meet the therapist team, during the Preparatory Period or in advance of

the appropriate Experimental Session in-person to be oriented to the role. An escort who is responsible only for transportation need not meet with the therapist team to perform that role. The participant will provide contact information for the appropriate support person(s) in advance of each Experimental Session.

Orienting the Support Person

In advance of the appropriate Experimental Session, the support person staying with the participant overnight will be required to meet with the therapist team in person. The support person will receive printed instructions, including contact information of the study physician, therapists, Principal Investigator, and Study Coordinator and what to do in the case of an emergency.

The support person will not provide psychotherapy to the participant. Minimal discussion is acceptable, but only if initiated by the participant. The support person should not interpret the participant's experience or act as therapist. The therapy team will discuss this explicitly with the support person and include this information in the written instructions.

The main roles of the support person include:

- Ensuring the participant has a comfortable place to sleep and discuss the plan for wakeup, breakfast, and travel time for the next day's Integrative Session
- Seeing to the participant's needs for food and liquids, including providing dinner and breakfast in accordance with the participant's dietary preferences
- Eating with the participant unless they ask to be left alone
- Cleaning up after the participant, including doing the dishes and handling bedding
- Keeping all participant information confidential
- Ensuring the participant is safely transported to and from the study site as appropriate (may be completed by a separate support person)
- Ensuring that the participant does not leave the overnight site without accompaniment and does not leave the participant alone at the overnight site. The participant may have privacy at the overnight site.
- Supervising the participant to ensure they do not consume drugs or alcohol on the night of the Experimental Session and that phone/computer time is limited
- Remaining sober throughout the entire overnight stay
- Remaining available to participant's needs throughout the night, but may sleep if the participant is sleeping

End of Experimental Sessions

Consistent with the main protocol, at the end of each Experimental Session, the therapy team and site physician will assess the participant to decide if they are physically and emotionally stable. If the participant is not stable, the therapy team and/or site physician will stay with the participant until stable or escalate for further care as appropriate. If it is in the best interest of the participant for them to spend the night at the study site, the therapists will ensure an appropriate stay is provided.

In the sub-study, if a participant is deemed medically and psychologically stable by the therapy team at the end of the Experimental Session, the participant will be escorted home via car, rideshare, or public transportation and will not remain overnight at the study site. The support person will be provided with an instruction sheet including how to contact the Clinical

Investigator, Site Physician, and/or therapy team to report any issues. The support person will stay overnight with the participant and be instructed to call 911 in the case of a psychiatric or medical emergency.

Each participant will be instructed to call the therapy team when they arrive where they will stay for the night. If the therapy team has not received a call within 2 hours after the participant has left the site, the therapists will call the participant and support person to confirm arrival. The therapy team will ask about the participant's emotional well-being and invite them to begin the process of integration through self-reflection, journaling, meditation, or other quiet activities. The therapy team will remind the participants that this evening should be an opportunity to rest and integrate and that they should avoid unnecessary stresses, chores, etc. The therapy team will also discuss this with the participant during the Preparatory and Experimental Sessions. The therapy team and site physician will remain on-call overnight to attend to any medical or psychological issues that may come up for the participant.

On the morning after the Experimental Session, the participant will be escorted to the study site via car, rideshare, or public transportation, as participants are not allowed to drive within 24 hours of administration of MDMA.

In the sub-study, if a participant is deemed to be not psychologically stable at the end of the Experimental Session, they will remain under the care of their therapy team. The therapy team will continue to evaluate the participant to make further disposition recommendations depending on the participant's psychological condition. This could include: continued observation, psychological support and/or psychopharmacologic interventions; or further evaluation for potential transfer as appropriate per site Standard Operating Procedures (SOPs). If the therapy team determines that the participant needs ongoing observation and psychological care at the same location as the Experimental Session, there will be the option for them to remain overnight with the therapy team in accordance with site SOPs. If the participant will not be leaving the site for the night, the therapy team will call the support person(s) to inform them and arrange appropriate transportation following the Integrative Session on the following day if appropriate.

Sub-Study Objective & Evaluation

The primary objective of this sub-study will be to evaluate the feasibility of conducting MDMA-assisted psychotherapy for PTSD among participants who do not remain at the study site overnight after Experimental Sessions.

This sub-study will include all participants enrolled and treated at two study sites. No formal analysis is planned to support this objective due to small sample size. However, case reports and AEs collected on the night of the Experimental Session will be evaluated to support feasibility. These reports will be used to explore feasibility of expanding the sub-study to include additional Phase 3 clinical sites and to support generalizability of potentially delivering this treatment to varied treatment settings post-approval.
