# Supplementary material for: Effects of MDMA-assisted therapy for PTSD on self-experience
Source: PLoS One. 2024 Jan 10;19(1):e0295926. doi: 10.1371/journal.pone.0295926 (PMC10781106; doi:10.1371/journal.pone.0295926)
Supplement: S1 File — The MAPP1 Statistical Analysis Plan summarizes the statistical analyses used to evaluate the MAPP1 study findings. (PDF) [file pone.0295926.s004.pdf]

MAPP1 Statistical Analysis Plan

Version 1: 19 January 2017
Version 2: 9 June 2017
Version 3: 23 July 2017
Version 4: 26 July 2017
Version 5: 25 September 2017
Version 6: 23 January 2018
Version 7: 22 May 2020

SPONSOR	Multidisciplinary Association for Psychedelic Studies (MAPS)<br>1115 Mission Street<br>Santa Cruz, CA 95060
SPONSOR REPRESENTATIVE	Rick Doblin, Ph.D.<br>Executive Director, MAPS
SPONSOR DELEGATE & TRIAL ORGANIZER	MAPS Public Benefit Corporation (MPBC)<br>1115 Mission Street<br>Santa Cruz, CA 95060
SPONSOR DESIGNEE	Amy Emerson<br>Executive Director, MPBC<br>Phone number: 510-393-7224<br>Email: amy@mapsbcorp.com
MEDICAL MONITOR(S)	Michael Mithoefer, M.D.
STATISTICIAN(S)	Scott Hamilton, Ph.D.
PREPARED BY	Scott Hamilton, Ph.D.<br>Berra Yazar-Klosinski, Ph.D.<br>Julie Wang, MPh, Ph.D.
USE	In conjunction with relevant FDA, HC, MOH guidance

Table of Contents

List of Tables **3**
List of Abbreviations **4**
1.0 Definitions of Terms **6**
2.0 Introduction **7**
3.0 Study Objectives **7**
3.1 Primary Objective 7
3.2 Secondary Objective 7
3.3 Safety Objectives 7
3.4 Exploratory Objectives 8
4.0 Measures **9**
5.0 Study Design **11**
6.0 Randomization and Blinding **15**
7.0 Sample Size and Power Considerations **15**
8.0 Analyses **16**
8.1 Analysis Sets 16
8.2 Missing Data Handling 16
8.2.1 Partial or Missing Dates 17
8.3 Protocol Deviations 17
8.4 Baseline Values 17
8.5 Participant Disposition and Dosing Summary 18
8.6 Demographics and Baseline Characteristics 18
8.7 Efficacy Analyses 18
8.7.1 Primary Efficacy Analyses 18
8.7.2 Supportive Analyses: 19
8.7.3 Key Secondary Efficacy Analyses 22
8.7.4 Responder Analyses 24
8.8 Safety Analyses 26
8.8.1 Analysis of Exposure 26
8.8.2 Analysis of Adverse Events 26
8.8.3 Concomitant Medications 26
8.8.4 Analysis of Suicidal Ideation and Behavior 27
8.8.5 Analysis of Vital Signs 27
8.9 Administrative Interim Analysis 27
9.0 Data Monitoring Committee **29**
10.0 Timing of Analyses **29**

List of Tables

Table 1: Protocol Objectives and Assessment Tools....9
Table 2: Phase 3 Dose Regimen of MDMA or Placebo....11
Table 3: Time and Events....12
Table 4: Proposed Estimands and Key Attributes....19
Table 5: Inference of Treatment Effect for Missing Data from mITT Set Based on Reasons for Post-Randomization Withdrawal....21
Table 6: Observed Effect Sizes and Corresponding Sample Size for 90% Conditional Power Guidance at the Interim Analysis (N=60)....28

List of Abbreviations

ACE	Adverese Childhood Experiences Questionnaire
AE	Adverse Event
AESI	Adverse Event of Special Interest
ANCOVA	Analysis of Covariance
AUDIT	Alcohol Use Disorders Identification Test
BDI-II	Beck Depression Inventory-II
BMI	Body Mass Index
BOCF	Baseline Observation Carried Forward
BP	Blood Pressure
CAPS-4	Clinician Administered PTSD Scale for DSM-4
CAPS-5	Clinician Administered PTSD Scale for DSM-5
CPGS	Chronic Pain Grade Scale
C-SSRS	Columbia Suicide Severity Rating Scale
DDIS	Dissociative Disorders Interview Schedule
DMC	Data Monitoring Committee
DSM-4	Diagnostic and Statistical Manual of Mental Disorders, 4th edition
DSM-5	Diagnostic and Statistical Manual of Mental Disorders, 5th edition
DSPI	Dissociative Subtype of PTSD Interview
DUDIT	Drug Abuse Disorders Identification Test
EAT-26	Eating Attitudes Test
EQ-5D-5L	EuroQol Five Dimensions – Five Levels Questionnaire
ES	Effect Size
GCP	Good Clinical Practice
HPQSF	Health and Work Performance Absenteeism and Presenteeism Short Form
IASC	Inventory of Altered Self-Capacities
IP	Investigational Product
IPF	Inventory of Psychological Functioning
IR	Independent Rater
ISG	Independent Statistical Group
IWRS	Interactive Web Randomization System
LEC-5	Life Events Checklist
LOCF	Last Observation Carried Forward
LS	Least Squares
MAPS	Multidisciplinary Association for Psychedelic Studies
MAR	Missing at Random
MDI	Multiscale Dissociation Inventory
MDMA	3,4-methylenedioxymethamphetamine
MedDRA	Medical Dictionary for Regulatory Activities
Mg	Milligram
*m*ITT	Modified Intent-to-Treat
MMRM	Mixed Model Repeated Measure
MNAR	Missing Not at Random
MPBC	MAPS Public Benefit Corporation
NPP	Not Per Protocol
PCL-5	PTSD Checklist for DSM-5
PMM	Pattern Mixture Model
PP	Per Protocol
PSQI	Pittsburgh Sleep Quality Index
PT	Preferred Terms
PTSD	Posttraumatic Stress Disorder

REML	Restricted Maximum Likelihood
SAE	Serious Adverse Event
SAP	Statistical Analysis Plan
SCS	Self-compassion Scale
SDS	Sheehan Disability Scale
SEM	Standard Error of Measurement
SOC	System Organ Class
SRNU	Self-reported Nicotine Use
TEAE	Treatment Emergent Adverse Event
TAS-20	Toronto Alexithymia Scale
UFEC	Utilization of Facility-based and Emergent Care
WHO DDE	WHO Drug Dictionary EnhancedTM
WOCF	Worst Observation Carried Forward

1.0 Definitions of Terms

Categorical data: refers to discrete (indivisible) variables, such as gender or ethnicity; data will be presented as total numbers of each category as needed to describe the sample

Completers: are defined as participants who complete all three planned experimental sessions and the CAPS-5 outcome assessment 18 weeks after randomization (Visit 19).

Descriptive data: includes mean, median, standard deviation, minimum, and maximum of numerical data used as needed to describe the sample

Difference scores: consist of scores computed by subtracting one value from another, as subtracting Baseline from follow-up score, used to test for differences between and within groups to determine change as a function of experimental treatment over time

Dropouts: are defined as participants who withdraw consent due to any reason after randomization and no longer participate in the study, i.e. no further contact with investigators or site staff.

Efficacy: type of analysis used to assess therapeutic effects or benefits

Exploratory analyses: inferential or descriptive analysis of the data to determine trends that might lead to hypotheses for further study

Frequency listing: tabular listing of numbers and/or percentages of events used as needed to describe the sample or data characteristics

Outcome measures: primary and secondary study measures that are used to test the study hypotheses

Post-randomization Early Terminators: are defined as participants who discontinue study treatment but continue to participate in study evaluations and outcome assessments.

Pre-randomization Early Terminators: are defined as participants who discontinue participation after enrollment but before randomization during the Preparatory Period and never receive study drug.

Process measures: study measures or qualitative observations collected during the study that may increase depth of understanding of the condition and treatment, although not necessarily related to safety or efficacy

Protocol deviation: event that represents significant divergence from the intended study design as described in the protocol

Safety: assessment of indicators of potential risks and adverse events

Safety measures: study measures that assess safety of the Investigational Product (IP), such as heart rate monitoring, blood pressure, body temperature

Study design: all elements of a research project that define the study question, experimental methods, study procedures including randomization and blinding, measurement techniques, data workflow, and statistical analysis

Tabular listing: list of each variable or item for each individual participant either in total or by treatment group in a table format

2.0 Introduction

This document contains a Statistical Analysis Plan (SAP) for the study, "A Randomized, Double-Blind, Placebo-Controlled, Multi-Site Phase 3 Study of the Efficacy and Safety of Manualized MDMA-Assisted Psychotherapy for the Treatment of Severe Posttraumatic Stress Disorder."

The Multidisciplinary Association for Psychedelic Studies (MAPS) is a non-profit research and educational organization working to obtain approval for the prescription use of 3,4-methylenedioxymethamphetamine (MDMA) in conjunction with psychotherapy in persons with posttraumatic stress disorder (PTSD). MAPS has delegated trial organization activities to its wholly owned subsidiary, MAPS Public Benefit Corporation (MPBC).

To confirm the efficacy and safety of this treatment for participants with severe PTSD, the sponsor is conducting this Phase 3 randomized, placebo-controlled, two-arm, double-blind, multi-site study with three monthly Experimental Sessions of psychotherapy combined with either MDMA or placebo, along with 12 non-drug psychotherapy sessions including preparatory and integrative sessions. The Primary Outcome measure, the Clinician Administered PTSD Scale (CAPS-5), evaluates PTSD symptom severity and is assessed by a blinded centralized Independent Rater (IR) pool.

3.0 Study Objectives

3.1 Primary Objective

The primary objective of this study is to evaluate the *de jure* efficacy of MDMA-assisted psychotherapy for PTSD compared to identical psychotherapy with inactive placebo, as measured by the reduction in CAPS-5 Total Severity Score from Visit 3 (Baseline) to Visit 19 (18 weeks post Baseline).

3.2 Secondary Objective

The key secondary objective of this study is to evaluate the efficacy of MDMA-assisted psychotherapy for PTSD compared to identical psychotherapy with inactive placebo in clinician-rated functional impairment, as measured by the mean change in Sheehan Disability Scale (SDS) item scores from Visit 3 (Baseline) to Visit 19 (18 weeks post Baseline).

3.3 Safety Objectives

The overall safety objective is to assess differences between groups in severity, incidence and frequency of Adverse Events (AEs), Treatment Emergent AEs (TEAEs), AEs of Special Interest (AESIs), and Serious Adverse Events (SAEs), concomitant medication use, suicidal ideation and behavior, and vital signs to support the package insert for MDMA-assisted psychotherapy. The following safety objectives will evaluate the safety of MDMA-assisted psychotherapy compared to identical psychotherapy with inactive placebo:

1. Compare relative incidence of AEs during Experimental Sessions that may be indicative of a medical complication of the Investigational Product (IP) such as clinical signs and

symptoms of chest pain, shortness of breath, or neurological symptoms or any other signs or symptoms that prompt additional vital sign measurements.

2. Compare relative incidence of AEs by severity.
3. Compare relative incidence of TEAEs, to determine relationship to the IP based on relative incidence in the MDMA group.
4. Compare relative incidence of TEAEs by severity reported during an Experimental Session, 1 and 2 days after IP administration.
5. Compare relative incidence of AESIs, defined as AEs specified in the protocol related to cardiac function and abuse liability.
6. Compare relative incidence of AEs by severity categorized as leading to discontinuation of IP, resulting in death or hospitalization, and continuing at Termination
7. Compare relative incidence of SAEs
8. Compare relative incidence of concomitant medications taken during an Experimental Session, 1 and 2 days after IP administration.
9. Compare relative incidence of psychiatric concomitant medications taken during the Treatment Period.
10. Compare relative incidence of positive or serious ideation and suicidal behavior assessed with the Columbia Suicide Severity Rating Scale (C-SSRS) by treatment group.
11. Compare mean changes in blood pressure, heart rate, and body temperature from pre-IP administration to end of each Experimental Session by treatment group.

3.4 Exploratory Objectives

The additional exploratory objectives of this study involve comparing participants receiving active dose MDMA to those receiving placebo to support the primary objective:

- Assess differences between treatment groups in change of dissociative symptoms associated with PTSD, as measured by the *de jure* estimand of change in Dissociative Subtype of PTSD Interview (DSPI) Total Score from Baseline (Visit 3) after 18 weeks (Visit 19) post-randomization
- Explore the correlation of dissociation symptoms as measured by the *de jure* estimand of change in DSPI with the change in CAPS-5 Total Severity scores from Baseline (Visit 3) after 18 weeks (Visit 19) post-randomization
- Characterization of CAPS-5 Total Severity score at weeks 6, 10, and 18 to explore onset of treatment efficacy with a descriptive time course plot.
- Explore the effect of adverse childhood experiences on treatment outcome, as measured by the Adverse Childhood Experiences Questionnaire (ACE) at screening, on the *de jure* estimand of change in CAPS-5 Total Severity scores from from Baseline (Visit 3) after 18 weeks (Visit 19) post-randomization
- Assess differences between treatment groups in change in depression symptoms, as measured by the *de jure* estimand of change in the Beck Depression Inventory-II (BDI-II) total score from Baseline (Visit 4) after 18 weeks (Visit 20) post-randomization
- Assess differences between treatment groups in change of sleep quality, as measured by the *de jure* estimand of change in Pittsburgh Sleep Quality Index (PSQI) total score from Baseline (Visit 4) after 18 weeks (Visit 20) post-randomization
- Assess differences between treatment groups in change in chronic pain, as measured by the *de jure* estimand of change in Chronic Pain Grade Scale (CPGS) total score from Baseline (Visit 4) after 18 weeks (Visit 20) post-randomization
- Explore the effect of presence of secondary traumatic stressors during the last month on the CAPS-5 Total Severity analyses, as measured by the Life Events Checklist (LEC-5) from Baseline (Visit 4) after 18 weeks (Visit 20) post-randomization

- Explore changes within-participants in PTSD symptom clusters of re-experiencing, avoidance, negative alterations in cognition and mood, and hyperarousal, as measured by the *de jure* estimand of change in CAPS-5 subscale scores from Baseline (Visit 3) after 18 weeks (Visit 19) post-randomization
- Assess differences between treatment groups in change in functioning in relation to self and others, as measured by the *de jure* estimand of change in Inventory for Altered Self-Capacities (IASC) subscale scores from Baseline (Visit 4) after 18 weeks (Visit 20) post-randomization
- Assess differences between treatment groups in change in alexithymia, as measured by the *de jure* estimand of change in Toronto Alexithymia Scale (TAS-20) total score from Baseline (Visit 4) after 18 weeks (Visit 20) post-randomization
- Assess differences between treatment groups in change in self-compassion, as measured by the *de jure* estimand of change in Self-Compassion Scale (SCS) total score from Baseline (Visit 4) after 18 weeks (Visit 20) post-randomization
- Assess differences between treatment groups in change in quality of life, as measured by the *de jure* estimand of change in EuroQol Five Dimensions – Five Levels Questionnaire (EQ-5D-5L) from Baseline (Visit 4) after 18 weeks (Visit 20) post-randomization
- Assess differences between treatment groups in change in eating habits, as measured by the Eating Attitudes Test (EAT-26) from Baseline (Visit 4) after 18 weeks (Visit 20) post-randomization
- Assess differences between treatment groups in change in drug use, as measured by the Drug Use Disorders Identification Test (DUDIT) total score from Screening to 18 weeks (Visit 20) post-randomization
- Assess differences between treatment groups in change in alcohol use, as measured by the Alcohol Use Disorders Identification Test (AUDIT) total score from Screening to 18 weeks (Visit 20) post-randomization
- Assess differences between treatment groups in change in nicotine use, as measured by the Self-reported Nicotine Use (SRNU) from Baseline (Visit 4) after 18 weeks (Visit 20) post-randomization
- Assess differences between treatment groups in change in workplace productivity, as measured by the Health and Work Performance Absenteeism and Presenteeism Short Form (HPQSF) from Baseline (Visit 4) after 18 weeks (Visit 20) post-randomization

4.0 Measures

Table 1: Protocol Objectives and Assessment Tools

**Objectives**	**Measure**	**Measure Type**	**Administration**
Eligibility			
Assess psychiatric disorders	MINI	Eligibility	Site
Assess personality disorders	SCID-5-PD with SCID-5-SPQ	Eligibility	Telemedicine (IR)/ self-report measure at Site
Confirm PTSD diagnosis and severity	PCL-5 with LEC-5	Eligibility	Site
Identify dissociative disorders	DDIS	Eligibility	Site

**Objectives**	**Measure**	**Measure Type**	**Administration**
Primary			
Assess changes in PTSD symptom severity from Visit 3 to Visit 19 compared between groups	CAPS-5	Outcome	Telemedicine (IR)
Secondary			
Assess changes in clinician-rated functional impairment from Visit 3 to Visit 19 compared between groups	SDS	Outcome	Telemedicine (IR)
Safety			
Compare relative incidence of positive or serious ideation and suicidal behavior between groups	C-SSRS	Safety	Site
Exploratory			
Characterization of CAPS-5 Total Severity score at weeks 6, 10, and 18 to explore onset of treatment efficacy with a descriptive time course plot	CAPS-5	Outcome	Telemedicine
Explore changes in PTSD symptom clusters of re-experiencing, avoidance, negative alterations in cognition and mood, and hyperarousal as measured by changes in CAPS-5 subscale scores	CAPS-5	Outcome	Telemedicine
Assess changes in severity of dissociative symptoms associated with PTSD from Visit 3 to Visit 19 compared between groups	DSP-I	Outcome	Telemedicine (IR)
Explore correlation of dissociative symptoms associated with PTSD with the CAPS-5 Total Severity analyses	DSP-I<br>CAPS-5	Outcome	Telemedicine (IR)
Explore the effect of presence of secondary traumatic stressors during the assessment period as a covariate on the CAPS-5 Total Severity analyses	LEC-5<br>CAPS-5	Outcome	Site or Telemedicine
Explore the effect of adverse childhood experiences on PTSD treatment outcomes as a covariate on the CAPS-5 Total Severity analyses	ACE<br>CAPS-5	Outcome	Site or Telemedicine
Assess changes in depression symptoms from Visit 4 to Visit 20 compared between groups	BDI-II	Outcome	Site or Telemedicine
Assess changes in chronic pain from Visit 4 to Visit 20 compared between groups	CPGS	Outcome	Site or Telemedicine
Assess changes in quality of life from Visit 4 to Visit 20 compared between groups	EQ-5D-5L	Outcome	Site or Telemedicine
Assess changes in functioning in relation to self and others from Baseline to Visit 20 compared between groups	IASC	Outcome	Site or Telemedicine
Assess changes in self-reported psychosocial functioning from Visit 4 to Visit 20 compared between groups	IPF	Outcome	Site or Telemedicine
Assess changes in self-compassion from Visit 4 to Visit 20 compared between groups	SCS	Outcome	Site or Telemedicine
Assess changes in alexithymia from Visit 4 to Visit 20 compared between groups	TAS-20	Outcome	Site or Telemedicine

Objectives	Measure	Measure Type	Administration
Assess changes in alcohol use from Screening to Visit 20 compared between groups	AUDIT	Healthcare cost	Site or Telemedicine
Assess changes in drug use from Screening to Visit 20 compared between groups	DUDIT	Healthcare cost	Site or Telemedicine
Assess changes in nicotine use from Visit 4 to Visit 20 compared between groups	SRNU	Healthcare cost	Site or Telemedicine
Assess changes in disordered eating from Visit 4 to Visit 20 compared between groups	EAT-26	Healthcare cost	Site or Telemedicine
Assess changes in workplace productivity from Visit 4 to Visit 20 compared between groups	HPQSF	Healthcare cost	Site or Telemedicine
Assess facility-based healthcare utilization at Screening	UFEC	Healthcare cost	Site or Telemedicine

5.0 Study Design

Table 2: Phase 3 Dose Regimen of MDMA or Placebo

Experimental Session	Initial Dose	Supplemental Dose*	Min-Max Cumulative Dose
1	80 milligrams (mg)	40 mg	80 mg to 120 mg
2	80 or 120 mg	40 or 60 mg	80 mg to 180 mg
3	80 or 120 mg	40 or 60 mg	80 mg to 180 mg
		**Total Cumulative Dose**	240 mg to 480 mg

* Unless contraindicated

Table 3: Time and Events

	**Screening Period** (up to 28 days)				**Preparatory Period w/ Enrollment Confirmation** (1-11 weeks)			
	**Screening**			**Enrollment**	**Preparatory**		**Baseline CAPS-5 T1**	**Baseline & Enrollment Confirmation**
**Visit**	Phone Screening	Screening	IR Screening	V0	V1	V2	V3	V4
**Visit Description**	Phone Calls	In-person Visits & Labs	Telemedicine	Enrollment	Prep. 1	Prep. 2	Telemedicine	Prep. 3 & Enrollment Confirmation
**Visit Timing**	Prior to Initial Screening	Over 1 to 4 wks	1 to 9 days after initial eligibility met	2 to 14 days post IR Screening	0 to 12 days after V0	2 to 21 days after V1	Post V2 & Taper	3 to 6 days after V3; 1 to 4 days before V5
Initial Phone Screen	□							
Informed Consent	Send Copy	□						
Follow-up Phone Screen	□							
Assess Eligibility	□	□		□	□	□		□
Medical/Psychiatric History	□ [A]	□			□	□		□
Past/Current Medication & Adherence	□	□			□	□		□
Weight, Resting Vitals		□						
Physical Exam		□						
ECG & Rhythm Strip		□						
Clinical Lab Tests		□						
Drug Screen		□						□
Pregnancy Screen		□						□
Enter Participant in eCRF [B]		□						
Record			□		□	□	□	□
Medication Taper				□	□	□		
Study Enrollment				□				□ Confirmed
All AEs [C]				□	□	□		□
90-min Preparatory Session					□	□		□
Phone Call Follow-up [D]						□		

[A] At Screening, collect data on previous hospitalizations and healthcare utilization. Request participants to obtain medical/psychiatric records to bring to the in-person screening.
[B] Participants will be entered into the eCRF after the IR visit is scheduled
[C] All Adverse Events (AEs) includes collecting Serious Adverse Events, AEs of Special Interest, AEs of Psychiatric Status, AEs requiring medical advice or attention, AEs that indicate withdrawal of a participant, and all other AEs
[D] If needed, call participant to confirm medication tapering and stabilization is complete prior to Visit 3

	**Treatment Period** ~12 weeks (+/-3 weeks)														**Follow-up Period & Study Termination** (~4 wks)	
	**Treatment 1**					**Treatment 2**					**Treatment 3**				**Primary Outcome**	**Study Termi-nation**
**Visit**	V5	V6	V7	V8	V9	V10	V11	V12	V13	V14	V15	V16	V17	V18	V19	V20
**Visit Description**	Exp. 1	Int. 1.1	Int. 1.2 [G]	CAPS-5 T2: Tele-medicine	Int. 1.3 [G]	Exp. 2	Int. 2.1	Int. 2.2 [G]	CAPS-5 T3: Tele-medicine	Int. 2.3 [G]	Exp. 3	Int. 3.1	Int. 3.2 [G]	Int. 3.3 [G]	CAPS-5 T4 Outcome: Tele-medicine [F]	Study Termi-nation
**Visit Timing**	4 to 7 days after V3; 1 to 4 days after V4	Morn-ing after V5	3 to 14 days after V5	18 to 30 days after V5	20 to 34 days after V5; 1 to7 days before V10	21 to 25 days after V5	Morn-ing after V10	3 to 14 days after V10	18 to 30 days after V10	20 to 34 days after V10; 1 to 7 days before V15	21 to 35 days after V10	Morn-ing after V15	3 to 14 days after V15	21 to 35 days after V15	105 to 147 days after V3; 42 to 70 days after V15	105 to 147 days after V3; 1 to 9 days after V19
Past/Current Medication & Adherence	☐	☐	☐		☐	☐	☐	☐		☐	☐	☐	☐	☐		☐
Drug Screen	☐					☐					☐					
Pregnancy Screen	☐					☐					☐					
Record	☐	☐	☐	☐	☐	☐	☐	☐	☐	☐	☐	☐	☐	☐	☐	
All AEs [A]	☐	☐	☐		☐	☐	☐	☐		☐	☐	☐	☐	☐		☐
Randomization [B]	☐															
Container Assignment [B]	☐					☐					☐					
Administer IMP	☐					☐					☐					
8-hour Exp. Session	☐					☐					☐					
BP, Pulse, Temperature [C]	☐					☐					☐					☐ [D]
Overnight Stay	☐					☐					☐					
90-min Integrative Session		☐	☐		☐		☐	☐		☐		☐	☐	☐		
Phone Call Follow-up [E]		☐					☐					☐				
Weight																☐

[A] All Adverse Events (AEs) includes collecting Serious Adverse Events, AEs of Special Interest, AEs of Psychiatric Status, AEs requiring medical advice or attention, AEs that indicate withdrawal of a participant, and all other AEs

[B] Randomize 24 to 48 hours prior to first Experimental Session; obtain container assignment 24 to 48 hours prior to each Experimental Session

[C] During Experimental Sessions, vitals are measured before Investigational Product administration, immediately before the supplemental dose is administered (or would be, if supplemental dose not given), and approximately 8 hours after initial dose, and as needed

[D] At Study Termination, only blood pressure needs to be measured

[E] 14 days of phone call follow-up: Days 2, 4, 6, 7, 8, 10, 12, 14 after each Experimental Session

[F] All visits must be scheduled to ensure that the Primary Outcome CAPS-5 T4 assessment is within the overall window provided: 105 to 147 days post Visit 3

[G] All Integrative Sessions must be at least 2 days apart. An Integrative Session and CAPS-5 assessment may take place on the same day (in the appropriate order, per protocol).[H] Four days of phone call follow-up: Day 2 and 7 after the Experimental Session, with two additional calls in between

[I] All visits must be scheduled to ensure that the CAPS-5 T4 assessment is within the window provided

		**Screening**		**Baseline & Enrollment Confirmation**		**Treatment 1**				**Treatment 2**				**Treatment 3**			**Follow-up & Study Termination**	
	**Visit #**	Site [A]	IR Screening	IR V3	V4	V5	V6&7	IR V8	V9	V10	V11&12	IR V13	V14	V15	V16&17	V18	IR V19	V20
**Visit Description**	**~Time to Complete Measure (minutes)**	Site Visit	Tele-medicine	Tele-medicine	Site Visit	Exp. Session 1	Int. Sessions 1.1 & 1.2	CAPS-5 T2: Tele-medicine	Int. Session 1.3	Exp. Session 2	Int. Sessions 2.1 & 2.2	CAPS-5 T3: Tele-medicine	Int. Session 2.3	Exp. Session 3	Int. Sessions 3.1 & 3.2	Int. Session 3.3	CAPS-5 T4: Outcome: Tele-medicine	Study Termi-nation
**CAPS-5**	90 (Baseline) 60 (all others)			□				□				□					□	
**SDS**	2			□				□				□					□	
**DSP-I**	15			□				□				□					□	
**C-SSRS** [B]	10	□	□		□	□[C]	□		□	□[C]	□		□	□[C]	□	□		□
**MINI**	15		□															
**SCID-5-SPQ**	20	□																
**SCID-5-PD**	60		□															
**LEC-5**	5	□							□				□			□		□
**PCL-5**	8	□																
**DDIS** [D]	5		□															
**ACE**	4				□													
**BDI-II**	10 (Baseline) 5 (all others)				□													□
**CPGS**	5				□													□
**EQ-5D-5L**	3				□													□
**IASC**	15				□													□
**IPF**	10				□													□
**SCS**	6				□													□
**TAS-20**	5				□													□
**AUDIT**	3	□																□
**DUDIT**	3	□																□
**SRNU**	3				□													□
**EAT-26**	6				□													□
**HPQSF**	5				□													□
**UFEC**	3				□													
**~Total Time of Completing Measures (minutes)**		**49**	**90**	**107**	**85**	**10**	**10**	**77**	**15**	**10**	**10**	**77**	**15**	**10**	**10**	**15**	**77**	**87**

[A] Ensure that LEC-5 and SCID-5-SPQ results are sent to the Independent Rater who will be conducting the SCID-5-PD

[B] First C-SSRS is a Lifetime assessment, other assessments are Since Last Visit

[C] Conducted pre- and post-Investigational Product administration, and at phone calls on Days 2, 4, 6, 7, 8, 10, 12, and 14 after Experimental Sessions

[D] The relevant questions (117-130) from the DDIS will be asked by the Independent Rater during the SCID-5-PD assessment. The entire measure will never be administered.

6.0 Randomization and Blinding

Eligible participants will be enrolled in the study and sequentially assigned an identification number. Participants will be assigned to MDMA or placebo treatment groups via an Interactive Web Randomization System (IWRS) based on a randomization schedule developed by an independent third-party vendor to maintain blinding. Randomization will be stratified by clinical site.

To minimize bias, protect the study's double-blind and to ensure data quality, the Sponsor plans to use a second Electronic Data Capture (EDC) database that is dedicated to the collection of critical primary and key secondary outcome measures, including the Total Severity Score on the CAPS-5 and item scores on the Sheehan Disability Scale (SDS), administered by the centralized blinded Independent Rater (IR) Pool through live video. This second database is termed the Independent Rater Database (IRDB) and it will be separate from the blinded, clinical EDC database in order to ensure that site and sponsor staff engaged in study conduct are masked from study outcomes. The IRDB will only be accessible by: (1) qualified, observer-blind individuals who are in the established IR Pool, (2) the Senior IR responsible for oversight and data quality of the IR Pool, and (3) the IR Coordinator responsible for data entry based on paper Source Records completed by the IR Pool. All CAPS-5 and SDS scores and supporting documentation will be reviewed by the IR Coordinator prior to data entry.

As a site-independent review method that evaluates and confirms diagnoses, symptom severity, and subject validity prior to randomization, the Senior IR will review endpoint data quality. Once data is entered into the IRDB, the Senior IR will oversee the data quality by centrally monitoring the critical data using a risk-based approach. The risk-based monitoring methodology will utilize the following strategies: 1) To perform remote, source data review of the IRDB to check logic and to confirm reliability between the IR's, 2) To perform remote, source data verification to check the IRDB for administrative, data entry, or transcription errors made by the Independent Rater Coordinator, and 3) To perform systematic line listing reviews for data trends and/or to trigger implementation of pre-defined, risk-mitigation strategies when specific data thresholds are exceeded.

7.0 Sample Size and Power Considerations

The statistical power calculations were made by fitting an MMRM model to CAPS-4 data from the Phase 2 study MP1 data to obtain covariance parameter estimates. CAPS-4 data was also converted to the CAPS-5 scale, by dividing the CAPS-4 score by 34 and multiplying by 20 to obtain the CAPS-5 score, and both methods came up with the same level of statistical power. The following inputs created the 90% statistical power estimate using the PASS 14 Mixed Models Repeated Measures module.

a. N=47 per group (allowing for 3 dropouts per treatment group)
b. Alpha = 0.0499
c. 1000 simulations
d. Change from baseline means of 5.5, 12.8, and 20.5 for placebo. Change from baseline means of 17.2, 31.1, and 37.4, for MDMA at visits 1, 2, and 3, respectively
e. Two levels of treatment factor (MDMA, Placebo)
f. Three within subject levels (3 treatment visits)
g. Subject level variance = 335
h. Variance of R (diagonal elements) = 280
i. Rho = 0.5
j. Unstructured covariance

Additional participants may be added to the sample size at the recommendation of the Data Monitoring Committee (DMC) from the results of an administrative interim analysis (Section 8.9) occurring after 60% of the *m*ITT participants have completed a final assessment of the Primary Outcome (Visit 19) and terminated treatment.

8.0 Analyses

Every effort will be made to ensure complete, accurate and timely data collection and to avoid missing data, to ensure the completeness of the data which can impact the integrity and accuracy of the final study analysis. The statistical analyses will be reported using summary tables, figures, and data listings. All analyses and tabulations will be performed using SAS® Version 9.4 or higher, and S-PLUS. In general, nominal variables will be described in terms of frequencies and percentages and analyzed using chi square analysis. Ordinal and non-normal continuous variables will be described using sample median and range and analyzed by non-parametric statistical tests. Approximately normal variables will be described using sample mean and standard deviations and analyzed by parametric statistical tests. Except for the primary efficacy analysis, all statistical tests will be two-sided and a difference resulting in a p-value of less than or equal to 0.05 will be considered statistically significant. All p-values will be rounded to and displayed in four decimals. If a p-value less than 0.0001 occurs it will be shown in tables as <0.0001. Data not subject to analysis according to this plan will not appear in any tables or graphs, but will be included in the data listings. Selected results may be presented graphically using standard graphical software.

Post-hoc exploratory analyses not identified in this SAP may be performed to further examine the study data. These analyses will be clearly identified as such in the final clinical study report.

8.1 Analysis Sets

- *Modified Intent-To-Treat* (*m*ITT): all randomized participants who receive IP in at least one blinded Experimental Session (Visit 5) and have at least one follow-up CAPS-5 assessment post-treatment
- *Per Protocol* (PP): all randomized participants who meet eligibility criteria, who receive IP in three Experimental Sessions, and have three follow-up CAPS-5 assessments post-treatment
- *Not Per Protocol* (NPP): all participants who are included in the *m*ITT set but not the PP set
- *Safety*: all participants who receive any IP
- *All Enrolled:* all participants who sign informed consent and are initially enrolled

8.2 Missing Data Handling

All possible procedures within Good Clinical Practice (GCP) will be used to minimize Post-randomization Early Termination. Based on Phase 2 data it is expected that up to 5% of enrolled and randomized participants will terminate early. Post-randomization Early Terminators will be compared to the Completers using baseline demographics and CAPS-5 Total Severity Score at baseline. For the Post-randomization Early Terminators, data collection by IRs will continue on the same schedule as planned through Study Termination visit procedures in order to limit missing data. All observed CAPS-5 data up to the point of discontinuation of treatment will be included in the MMRM model of the *de jure* estimand from Post-randomization Early Terminators. Participants will not be replaced, enrollment and treatments will continue until N=100 participants are obtained in the *m*ITT set. All observed CAPS-5 data from Post-

randomization Early Terminators collected prior to and after early termination will be included in the supportive effectiveness analysis of the *de facto* estimand.

8.2.1 Partial or Missing Dates

The following conventions will be used to impute missing portions of dates for AEs and concomitant medications. Note that the imputed values outlined here may not always provide the most conservative date. In those circumstances, the imputed value may be replaced by a date that will lead to a more conservative analysis.

Start Dates:

1. If the year is unknown, then the date will not be imputed and will be assigned a missing value.
2. If the month is unknown, then:
    a. The month and day of the first dose date will be imputed if the year matches the first dose date year.
    b. Otherwise, 'January' will be assigned.
3. If the day is unknown, then:
    a. The day of the first dose date will be imputed if the month and year match the first dose date month and year.
    b. Otherwise, the first day of the month will be assigned.

Stop Dates:

1. If the year is unknown, then the date will not be imputed and will be assigned a missing value.
2. If the month is unknown, then 'December' will be assigned.
3. If the day is unknown, then the last day of the month will be assigned.

8.3 Protocol Deviations

A major deviations will be defined as participants enrolled and randomized but did not meet eligibility critera during the course of the study. The number of participants in each protocol deviation category listed below will be summarized by analysis set and treatment group. Individual participants will appear in a listing.

Possible protocol deviations include the following categories:

- Participant entered study but did not meet criteria
- Participant developed withdrawal criteria but was not withdrawn
- Participant received excluded concomitant treatment
- Protocol procedure not performed per protocol
- Participant received incorrect treatment or incorrect dose
- Protocol procedure performed out of range
- Miscellaneous

8.4 Baseline Values

Baseline values are from Baseline Visits for all measures, except C-SSRS, AUDIT, DUDIT, resting blood pressure (BP), Body Mass Index (BMI). For C-SSRS, the initial screening visit will be conducted with the 'Lifetime' version of the measure. The Independent Rater Screening assessment of 'Since Last Visit' suicidal ideation and behavior will be used as 'Baseline.' For the

AUDIT and DUDIT measures and resting BP, BMI, results collected at Screening will be used as the Baseline value.

8.5 Participant Disposition and Dosing Summary

The All Enrolled Set will be included in the summary of participant disposition and accountability. The tabulation of number of participants in each treatment group and overall will be displayed for the Safety Set and in the *m*ITT Set. The number and percent of participants who completed or discontinued the study will be displayed for each treatment group and overall together with reasons for early termination, where the percent is with respect to the total number of randomized participants in that treatment group. The timepoint of doses and total MDMA (mg) administered will be summarized by treatment group for the Safety and *m*ITT Sets.

8.6 Demographics and Baseline Characteristics

Participant demographic data and Baseline characteristics will be summarized descriptively by treatment group and overall. The demographic data and baseline characteristics will be summarized for the *m*ITT and Safety Sets.

8.7 Efficacy Analyses

For all primary, secondary, and exploratory endpoints descriptive statistics (n, mean, standard deviation, median, range, or counts and percentages where appropriate) will be provided by treatment group. Longitudinal CAPS-5 Total Severity Score and mean SDS item scores will be plotted across visits to characterize the onset of treatment effect.

8.7.1 Primary Efficacy Analyses

The *de jure* estimand of treatment efficacy will be used to estimate the causal effect of MDMA-assisted psychotherapy on PTSD symptom severity in the intended population of patients with PTSD from any cause (Estimand 1 in Table 4). All efficacy analyses will be based on the *m*ITT analysis set. The primary treatment comparison will be made at a 2-sided, 0.0499 level of alpha. The primary estimator of effects of initially randomized treatments will be the difference between groups in mean change in CAPS-5 Total Severity Scores from Baseline to 18 weeks after randomization (Visit 19). Least squares means from a restricted maximum likelihood (REML)-based mixed model for repeated measures (MMRM) will be used to compare treatment groups at Visit 19. CAPS-5 data collected after treatment discontinuation will not be included in the *de jure* estimand. Missing data will not be imputed. The model is specified as follows: Let Y_{ij} be the change in the CAPS-5 score from the *i*th participant at the *j*th visit, $i = 1, \ldots, n$ and $j = 1, 2, 3$, corresponding to post-baseline visits 8, 13, and 19, respectively. The treatment variable X_i is the randomized treatment of the *i*th participant. The variables T_1 and T_2, are variables indicating if the data from that record are from visit 13, or 19, respectively. Covariates included in the MMRM model are the continuous baseline CAPS-5 Total Severity Score and the binary variable indicating dissociative subtype at baseline. Investigative site is included as a fixed effect. The regression model is specified as follows:

$$Y_{ij} = \alpha + \beta_1 X_i + \beta_{21} T_1 I(j=2) + \beta_{22} T_2 I(j=3) + \beta_{31} T_1 X_i I(j=2) + \beta_{32} T_2 X_i\, I(j=3)$$
$$+ \beta_4 Baseline\_CAPS_i + \beta_5 DS_i + \varphi Site_i + \varepsilon_{ij},$$

where I(j=J) is the indicator function that equals 1 if j=J and 0 otherwise. We assume that the Y_{ij} follow a Normal distribution with var(Y_{ij}) = $\sigma_j 2$, and cov(Y_{ij}, $Y_{ij'}$) = $\sigma_{jj'}$=$\sigma_{j'j}$ (unstructured correlation). The model above has been parameterized with reference cell coding where the coefficient estimate of β_1 corresponding to the treatment variable, X, captures the mean difference in the change from baseline CAPS-5 for MDMA vs. placebo. The coefficient estimates of β_{21} and β_{22} corresponding to the visit variables, T_1 and T_2 capture the difference in mean change in the CAPS-5 at visits 13 and 19 from the mean scores at visit 8, respectively. The coefficient estimates of β_{31} and β_{32} corresponding to the interaction of the treatment variable and the visit variable capture the incremental mean difference in the change in CAPS-5 for the MDMA group at visits 13 and 19 from visit 8 versus the placebo group. The coefficient estimate of β_4 captures the effect on the change in CAPS-5 post baseline corresponding to the baseline level of CAPS-5 score. The coefficient estimate of β_5 captures the effect on the change in CAPS-5 post baseline corresponding to dissociative subtype at baseline. The parameter φ captures the the differences in mean change in CAPS-5 due to site differences. The following SAS code will implement this model and the statistical test for the MDMA vs. placebo comparison at Visit 19.

```
proc mixed;
  class ID X T Site DS;
    model CAPS = X  T  X*T Baseline_CAPS DS Site / s  DDFM=SATTERTH ;
    repeated T / subject=ID type=un;
    estimate 'MDMA vs. Placebo at Visit 19'  X  1 -1  X*T  0 0 1 0 0 -1 / e;
run;
```

where, the CAPS variable is the change from baseline in the CAPS-5 total severity score at each visit, the ID variable uniquely identifies the participant, Baseline_CAPS is the baseline CAPS-5 Total Severity Score for each participant, DS is the dummy variable indicating dissociative subtype at baseline, and Site indicates the clinical site where the participant was treated. There is a record for each visit for every participant. If the unstructured variance/covariance model assumption doesn't allow the REML algorithm to converge, a heterogeneous Toeplitz structure will be used. If the model doesn't converge under heterogeneous Toeplitz, the homogeneous Toeplitz structure will be used. If that fails to converge, the next variance/covariance attempted will be AR(1). In the event AR(1) structure fails to converge, the compound symmetry assumption will be used.

Table 4: Proposed Estimands and Key Attributes

Estimand	Hypothesis	Inference	Population	Endpoint	Use of data after early termination in analysis
1	*de jure*	Initially randomized treatment	mITT	Planned endpoint	Not included
2	*de facto*	Treatment Policy	mITT	Planned endpoint	Included

8.7.2 Supportive Analyses:

To support the need to provide information on treatment effectiveness in all randomized participants that could be expected in clinical practice, regardless of adherence to the 3-session

course of MDMA-assisted psychotherapy treatment, we propose a *de facto* estimand is included as an exploratory supportive analysis (Estimand 2 in Table 4).

The *de facto* estimand will include all available CAPS-5 outcome data from all participants in the mITT set regardless of adherence to the treatment, including CAPS-5 data collected after treatment discontinuation in case of early termination. This will be estimated in the *m*ITT set by a statistical comparison between treatment groups using the MMRM model with a weighted least squares estimate of the change in CAPS-5 scores from baseline to 18 weeks after randomization across all the post treatment CAPS-5 measures. The sponsor expects no more than 5% of the participants will discontinue treatment after the first experimental session and CAPS-5 outcome assessment, due to the highly-controlled treatment setting which is analogous to residential or in-patient treatment settings. Additionally, some participants may choose to discontinue after the first experimental session because of strong and early positive response. These participants are treatment successes and should not be modeled as treatment failures. Using the least squares estimate of the mean reduction in CAPS-5 scores across all treatment sessions will capture these early successes in the *de facto* estimand. It can be argued that because all participants in the study will have continued data collection and all observed post-treatment CAPS-5 scores included in the *de facto* estimand that there is no missing data in this analysis. Thus the *de facto* estimand will support the primary *de jure* estimand.

1. The choice of primary estimand will be tested with a supportive analysis including a different estimand:
    a. To determine *de facto* effectiveness by comparing MDMA-assisted psychotherapy to identical psychotherapy with inactive placebo for PTSD, as measured by the estimand of change in CAPS-5 Total Severity Score from Baseline after 18 weeks post-randomization, including all observed data after early termination.
2. The effect of departures from choices and assumptions made for the primary analysis will be tested as sensitivity analyses for the primary estimand. No sensitivity analyses will be performed for the secondary efficacy endpoints. Details of the sensitivity analyses with multiple imputation will be provided in the Final SAP.
    a. Distributional assumptions for the full data will be tested, if distribution found not to be normal, we will test a transformation of the data, such as the log transformation, to achieve normality.
    b. If outlying or influential observations are found: (1) a log transformation will be applied to the full data and (2) outliers will be excluded
    c. Effect of covariates will be tested by conducting the primary analysis with and without: (1) dissociative subtype of PTSD based on CAPS-5 and (2) Baseline CAPS-5 Total Severity Score.
    d. To examine the pre and post COVID-19 effect on the treatment effect the full model will be fitted with an additional binary dummy variable indicating whether the CAPS-5 measure was taken pre or post COVID-19. We chose to indicate the date of delineation between pre and post COVID-19 as March 11, 2020 – the day the World Health Organization declared COVID-19 a pandemic, and coincidently, the data cutoff date of the Interim Analysis. The COVID-19 binary variable will be added to the full model specified for the primary analysis in a sensitivity analysis as a fixed effect along with an interaction term with the treatment variable. If the interaction term is not significant at the alpha 0.05 level it will be dropped from the model. The estimate of the COVID-19 dummy variable will be clinically interpreted and included in the interpretation and discussion of the primary efficacy results.
    e. A concordance analysis will be executed to examine the effect of not completing all three (3) treatment sessions (treatment dropouts) on the treatment effect as measured by CAPS-5 [1]. The objective of the concordance analysis will be to address what

the treatment effect would have been if all the treatment dropouts had completed the treatments and CAPS-5 measures. This will be compared to a worst-case scenario.

f. To examine the robustness of the inferences to the MAR assumption, Jump to Reference and Copy Reference multiple imputation methods will be employed to ensure the most appropriate methodology is selected to test assumptions about missing data mechanism [2, 3]. Residual treatment effects are likely to carry over from observed periods during the study to the unobserved period after data collection ends for a dropout in some cases. Imputation method choice will depend on individual reasons for withdrawal (see Table 5). 1000 complete datasets will be generated; each of which will be analyzed using an ANCOVA model with effects for treatment arm, visit, and covariates. The final results will be obtained by combining the least squares (LS) means and LS mean differences from these 1000 analyses using standard multiple imputation methodology, and overall inference will be obtained by applying Rubin's Rules on the estimates obtained from each imputed/complete data set [4].

Table 5: Inference of Treatment Effect for Missing Data from mITT Set Based on Reasons for Post-Randomization Withdrawal

**Post-randomization Events**	**Available Data Post-Withdrawal**	**Treatment Assignment**	**Inference of Treatment Effect if Data is Missing (CAPS-5 Post Session 2 or 3)**
Discontinue Study, Withdraw Consent	No data	MDMA	Assume treatment failure<br>• Jump to Reference Placebo arm
		Placebo	Assume treatment failure<br>• Copy Reference Placebo arm
Discontinue Intervention (or experiences long delay e.g., due to a pandemic) and does not receive Rescue Medication	Data collection continues whenever possible via remote visits	MDMA	Assume treatment success if post-treatment CAPS and reason supports treatment response<br>• Copy Reference MDMA arm<br>Assume lack of tolerability if post-treatment CAPS and reason for dropping out do not support treatment response<br>• Jump to Reference Placebo arm
		Placebo	Assume treatment failure<br>• Copy reference Placebo arm
Discontinue Intervention (or experiences long delay e.g., due to a pandemic) and receives Rescue Medication	Data collection continues whenever possible via remote visits	MDMA	Assume treatment success if reason supports treatment response<br>• Copy Reference MDMA arm<br><br>Assume treatment failure if reason does not support treatment response<br>• Jump to Reference Placebo arm
		Placebo	Assume treatment failure<br>• Copy Reference Placebo arm
Death	No data	MDMA	Assume treatment success if post-treatment CAPS supports treatment response<br>• Copy Reference MDMA arm<br>Assume lack of tolerability if post-treatment CAPS and reason for dropping out do not support treatment response<br>• Jump to Reference Placebo arm
		Placebo	Assume treatment failure<br>• Copy reference Placebo arm
Suicide	No data	MDMA	Assume treatment failure<br>• Jump to Reference Placebo arm

Post-randomization Events	Available Data Post-Withdrawal	Treatment Assignment	Inference of Treatment Effect if Data is Missing (CAPS-5 Post Session 2 or 3)
		Placebo	Assume treatment failure<br>• Copy Reference Placebo arm

In exploratory analyses, additional baseline covariates (age, gender, ethnicity, index trauma, complexity and severity of trauma, medication tapering, diagnosis of comorbid depression, diagnosis of comorbid Axis 2 diagnosis, adverse childhood experiences) may be assessed for inclusion in the model at a $p<0.05$.

8.7.3 Key Secondary Efficacy Analyses

For the key secondary analysis of efficacy, the de jure estimand will be used to estimate the causal effect of MDMA-assisted psychotherapy on PTSD on the SDS in the intended population of patients with PTSD from any cause (Estimand 3 in Table 4). The SDS is a 3-item scale measuring the severity of disability in the domains of work, family life/home responsibilities and social/leisure activities. Each of these three domains is scored on a ten-point Likert scale, where a score of 0 is 'not at all impaired', 5 is 'moderately impaired' and 10 is 'very severely impaired'. The summary measure used to analyze the treatment effect on SDS will be the mean of the 3 item responses at each visit. Any participant who did not work during the reporting period due to reasons related to PTSD will be scored as a 10 on item 1. In cases where participants did not work due to reasons unrelated to PTSD, the score for item 1 (work/school) will be imputed by averaging the scores of items 2 and 3. It is expected that no more than 5% of participants will have item 1 (work/school) imputed, based on prior studies of paroxetine with PTSD. To limit missing data and ensure standardized administration, the SDS will be administered in a clinician-rated format during the same visits as the CAPS-5 by the centralized Independent Rater Pool. The clinician-rated administration will be important in limiting misinterpretation of the Work/school Impairment item that could contribute to elevated rates of skipping out, which can also be completed in reference to school, unpaid or volunteer work, and may not immediately come to mind in a participant-reported format.

If item 1 of the SDS is imputed in more than 5% of the participants, multiple imputation methods will be explored as an additional sensitivity analyses. If more than 5% of Item 1 data is missing, the frequency of participants not working due to reasons unrelated to PTSD and related to PTSD will be tabulated for both overall and within each visit. Additionally, a sensitivity analysis will be conducted to explore the effect of the different distributions for average SDS depending on whether the respective checkboxes for item 1 are checked or not. Separate MMRM models for the two types of responses to item 1 will be run and the treatment effect will be the weighted average of the two groups with the weights calculated as the frequency of checked item 1's.

A hierarchical testing strategy will be employed to control for type-I error. That is, the hypothesis for the Key Secondary Endpoint (SDS) will only be tested if the statistical test for the Primary Efficacy comparison rejects the null hypothesis. If the statistical test for the Primary Efficacy comparison does not reject the null hypothesis, the analysis of the Key Secondary Endpoint (SDS) will be exploratory. The treatment comparison will be made at a 2-sided, 0.0499 level of alpha. The key secondary estimator of effects of initially randomized treatments will be the difference between groups in mean change in the SDS summary measure from Baseline to 18 weeks after randomization (Visit 19). Least squares means from a restricted maximum likelihood (REML)-based mixed model for repeated measures (MMRM) will be used to compare treatment groups at Visit 19. SDS data collected after treatment discontinuation will not be included in the de jure estimand. Missing data will not be imputed and will be considered missing at random. The model is specified as follows: Let Yij be the change in the SDS summary measure from the ith

participant at the jth visit, i = 1, ..., n and j = 1, 2, 3, corresponding to post-baseline visits 8, 13, and 19, respectively. The treatment variable Xi is the randomized treatment of the ith participant. The variables T1 and T2, are variables indicating if the data from that record are from visit 13, or 19, respectively. Covariates included in the MMRM model are the continuous baseline SDS summary measure and the binary variable indicating dissociative subtype at baseline. Investigative site is included as a fixed effect. The regression model is specified as follows:

$$Y_{ij} = \alpha + \beta_1 X_i + \beta_{21} T_1 I(j=2) + \beta_{22} T_2 I(j=3) + \beta_{31} T_1 X_i I(j=2) + \beta_{32} T_2 X_i\ I(j=3)$$

$$+ \beta_4 Baseline\_SDS_i + \beta_5 DS_i + \varphi Site_i + \varepsilon_{ij},$$

where I(j=J) is the indicator function that equals 1 if j=J and 0 otherwise. We assume that the Yij follow a Normal distribution with $var(Y_{ij}) = \sigma j_2$, and $cov(Y_{ij}, Y_{ij'}) = \sigma_{jj'} = \sigma_{j'j}$ (unstructured correlation). The model above has been parameterized with reference cell coding where the coefficient estimate of β1 corresponding to the treatment variable, X, captures the mean difference in the change from baseline SDS measure for MDMA vs. placebo. The coefficient estimates of β_{21} and β_{22} corresponding to the visit variables, T1 and T2 capture the difference in mean change in the SDS summary measure at visits 13 and 19 from the mean scores at visit 8, respectively. The coefficient estimates of β_{31} and β_{32} corresponding to the interaction of the treatment variable and the visit variable capture the incremental mean difference in the change in the SDS summary measure for the MDMA group at visits 13 and 19 from visit 8 versus the placebo group. The coefficient estimate of β_4 captures the effect on the change in the SDS summary measure post baseline corresponding to the baseline level of the SDS summary measure. The coefficient estimate of β_5 captures the effect on the change in SDS summary measure post baseline corresponding to dissociative subtype at baseline. The parameter φ captures the the differences in mean change in SDS summary measure due to site differences. The following SAS code will implement this model and the statistical test for the MDMA vs. placebo comparison at Visit 19.

```
proc mixed;
  class ID X T Site DS;
     model SDS = X  T  X*T Baseline_SDS DS Site / s  DDFM=SATTERTH ;
     repeated T / subject=ID type=un;
     estimate 'MDMA vs. Placebo at Visit 19'  X  1 -1  X*T  0 0 1 0 0 -1 / e;
run;
```

where, the SDS variable is the change from baseline in the SDS summary measure at each visit, the ID variable uniquely identifies the participant, Baseline_SDS is the baseline SDS summary measure for each participant, DS is the dummy variable indicating dissociative subtype at baseline, and Site indicates the clinical site where the participant was treated. In the data used to fit the model, there is a record for each visit for every participant. If the unstructured variance/covariance model assumption doesn’t allow the REML algorithm to converge, a heterogeneous Toeplitz structure will be used. If the model doesn’t converge under heterogeneous Toeplitz, the homogeneous Toeplitz structure will be used. If that fails to converge, the next variance/covariance attempted will be AR(1). In the event AR(1) structure fails to converge, the compound symmetry assumption will be used.

8.7.4 Responder Analyses

The CAPS-5 produces a Total Severity Score based on severity of PTSD symptom domains described in the DSM-5, as well as a categorical rating indicating whether a participant meets PTSD diagnostic criteria. A psychometric validation study found the following severity score ranges for the CAPS-5: Asymptomatic (0-10), Mild (11-22), Moderate (23-34), Severe (35-46), Extreme (47+). Based on these data, a 10-point reduction in CAPS-5 Total Severity Score is clinically meaningful. As an alternate definition of treatment response to assess clinical significance of study outcomes, the sponsor will conduct a responder analysis to support findings from the primary efficacy analysis, and to explore how many single-dose treatments are needed to achieve response (See Figure 1). A similar responder analysis will be conducted for overall change in SDS item scores.

Figure 1: CONSORT for PTSD Responder Analysis After Treatment

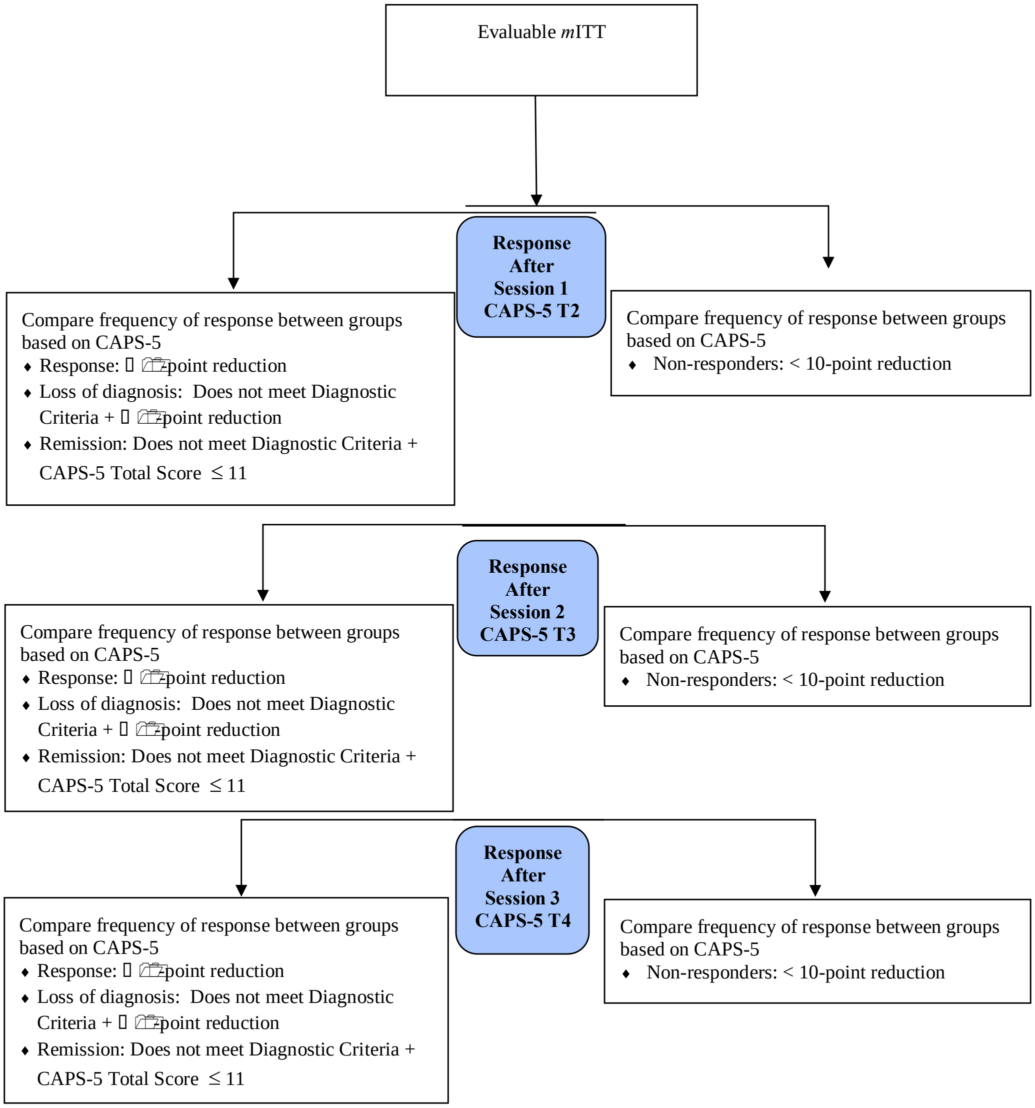

[1] Response at T2, T3, and T4 will be in comparison to Baseline at T1.

The sponsor will measure changes in functional impairment with the SDS throughout treatment at the same visits as the CAPS-5 in this study. The authors of the SDS have provided the following

score ranges to describe functional impairment severity for each of three items describing the functional domains of work, social life, and family life: No impairment (0), Mild impairment (1-3), Moderate impairment (4-6), Marked impairment (7-9), Extreme impairment (10). Based on the psychometric development publication [5] and prior Paroxetine studies for treatment of PTSD, a 4-point mean reduction across SDS items would represent a clinically meaningful treatment response, as this would correspond to downward change from Extreme to Moderate, Marked to Moderate, or Moderate to Mild in severity of any domain of functional impairment. If a participant drops to a mean score of less than 3 across SDS items, this would be defined as a functional remission indicating Mild or No impairment.

Figure 2: CONSORT for Functional Impairment Responder Analysis After Treatment

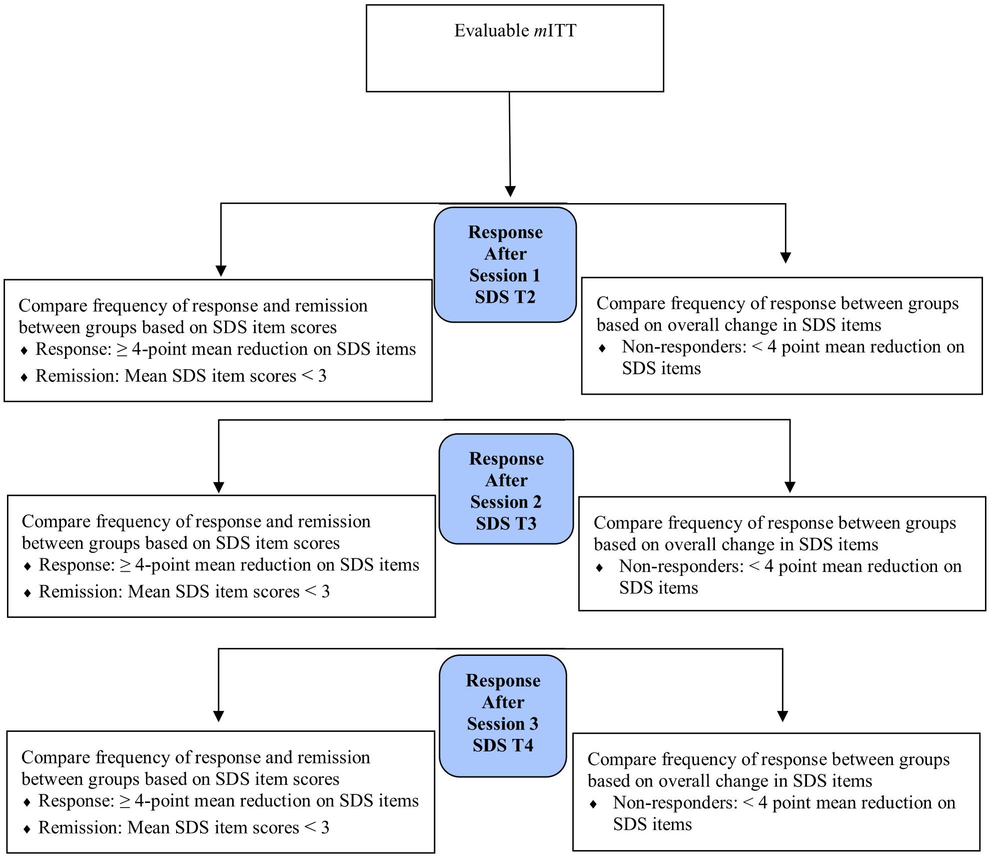

[1] Response at T2, T3, and T4 will be in comparison to Baseline at T1.

As an alternate definition of treatment response to assess clinical significance of study outcomes, the sponsor will conduct an exploratory responder analysis to support findings from the key secondary efficacy analysis, to explore the onset of clinically meaningful functional improvement (Figure 2). To further support the exploration of a clinically meaningful functional improvement in the PTSD population, results will be plotted in a cumulative distribution function plot to examine the magnitude of differences between treatment groups. Distribution-based methods including standard error of measurement (SEM) and one standard deviation away from the mean SDS scores at baseline will be reported. Ceiling effects will be assessed based on 40% of responses at most endorsing Extreme Impairment and floor effects will be assessed based on less than 5% of responses endorsing No Impairment on each item.

8.8 Safety Analyses

Safety assessment analyses for this study will include summaries of unsolicited adverse events, concomitant medications, suicidal ideation and behavior, and vital signs. Qualitative safety analyses will examine safety data with summary tables listing drug exposure, concomitant medications/therapies, unsolicited AEs, and percentages tabulated overall and by group.

8.8.1 Analysis of Exposure

The frequencies and percentages of participants with exposure will be summarized overall and by dose group. Data will be tabulated for the *m*ITT Set.

8.8.2 Analysis of Adverse Events

The primary measure of safety will be the reporting of unsolicited AEs. All AEs collected from Enrollment to Termination will be categorized as follows:

- Pretreatment AEs are defined as AEs that occur during the Preparatory Period prior to the first dose in the first Experimental Session
- Treatment Emergent AEs are defined as AEs that occur during the Treatment Period from the first Experimental Session to the last Integrative Session
- AEs that occur on and two days after MDMA or placebo administration
- AESIs are defined as AEs specified in the protocol related to cardiac function and abuse liability
- Follow-up Period AEs are defined as AEs that occur during the Follow-up Period after the last Integrative Session through Termination
- AEs leading to discontinuation of IP
- AEs resulting in death or hospitalization.
- SAEs
- AEs continuing at Termination.

Verbatim terms on case report forms will be mapped to preferred terms (PT) and system organ class (SOC) using the Medical Dictionary for Regulatory Activities (MedDRA) Version 17.0. Frequency and incidence of AEs will be displayed by PT, sorted by SOC, and summarized by treatment group, analysis set, category (as defined above), severity, and seriousness. AEs will be analyzed and presented as follows:

- If a participant has more than one AE mapped to the same PT, that AE will be reported once using the highest severity
- Relationship will be determined based on relative incidence of TEAEs with at least two-fold difference between MDMA vs. placebo
- Compare relative incidence of AEs during Experimental Sessions such as clinical signs and symptoms, such as chest pain, shortness of breath, or neurological symptoms or any other signs or symptoms that may be indicative of a medical complication of the investigational product
- AEs that occur on day of Experimental Sessions and two days after IP administration will be presented separately.

8.8.3 Concomitant Medications

A secondary measure of safety will be the reporting of concomitant medications. All concomitant medications collected from Screening to Study Termination will be categorized as follows:

- Pretreatment medications are defined as medications taken prior to and after signing informed consent and those taken during the Preparatory Period prior to the first Experimental Session. A stop date is expected prior to the first Experimental Session for any medications requiring a change in dose, a skipped dose, or tapering.
- Treatment Period concomitant medications are defined as those taken or continued during the Treatment Period from the first Experimental Session to the last Integrative Session. A stop date is expected prior to the each Experimental Session for any medications requiring a change in dose, a skipped dose, or tapering.
- Concomitant medications with a start date of the day of and two days after IP administration
- Follow-up Period concomitant medications are defined as those taken or continued during the Follow-up Period after the last Integrative Session through Termination
- Any concomitant medications that are tapered
- Any concomitant medications that are taken to treat an AE
- Any concomitant medications that are taken to treat an SAE
- Any excluded concomitant medications taken as a deviation from the protocol.

Concomitant medications on case report forms will be classified using the WHO Drug Dictionary Enhanced™ (WHO DDE). Frequency and incidence of concomitant medications will be displayed by generic name, sorted by class, and summarized by treatment group, analysis set, and category as defined above. Concomitant medications will be analyzed and presented as follows:

- Concomitant medications taken on the day of and two days after IP administration will be presented separately
- Any psychiatric concomitant medications by period.

8.8.4 Analysis of Suicidal Ideation and Behavior

Suicidal ideation and behavior will be summarized according to suggestions made in the C-SSRS Scoring and Data Analysis Guide [6]. A positive response for suicidal ideation is counted when a participant answers “yes” to any one of the five suicidal ideation questions (Categories 1-5) on the C-SSRS (i.e., a score >0 for suicidal ideation). Serious suicidal ideation is a suicidal ideation score of 4 or 5. A positive response for suicidal behavior occurs when a participant answers “yes” to any one of the five suicidal behavior questions (Categories 6-10) on the C-SSRS (i.e., a score >0 for suicidal behavior). The number and percent of positive responses of Positive Ideation, Serious Ideation, and Positive Behavior will be tabulated by treatment group and time period (lifetime, screening, baseline, each Experimental Session (pre- and post-IP), Integrative Sessions, and endpoints). Frequency and incidence of positive or serious ideation and suicidal behavior will be presented using descriptive statistics in tabular format.

8.8.5 Analysis of Vital Signs

Vital signs (heart rate, BP, and body temperature) for Experimental Sessions will be summarized using descriptive statistics in tabular format listing values at pre-IP, prior to the supplemental dose, and at the end of each Experimental Session by treatment group. Change from Baseline in vital signs will be compared by treatment group with appropriate statistical techniques.

8.9 Administrative Interim Analysis

An unblinded administrative interim analysis is planned to be performed at a single time point after the first 60% of participants in the *m*ITT set have completed the final CAPS-5 follow-up assessment and terminated treatment, including early termination participants who have

completed their final CAPS-5, and 100 participants have been enrolled. The objective of the administrative interim analysis is to conduct a sample size re-estimation. The key secondary endpoint will not be evaluated at the interim analysis. The detailed results of the interim analysis will not be revealed to the site staff, participants, IRs, or sponsor staff/designees except for a single designated sponsor representative from MAPS. The independent DMC statistician will perform the unblinded administrative interim analysis as described in the DMC Charter. In addition, procedures will be developed to ensure the blinding of the participant level data for parties outside of the Independent Statistical Group (ISG).

Sample size re-estimation: Based on the result of calculating conditional power using the estimated effect size from the first 60% of participants in the *m*ITT set, the sample size may be increased. The table below shows the range of possible effect sizes that could be observed at the interim analysis and what the guidance from the DMC could be for the adjusted sample size.

Table 6: Observed Effect Sizes and Corresponding Sample Size for 90% Conditional Power Guidance at the Interim Analysis (N=60)

Effect Size	≤ 0.32	0.32	0.34	0.36	0.38	0.40	0.42	0.44
Mean Change	-	5.76	6.12	6.48	6.84	7.2	7.56	7.92
Conditional Power[1]	< 50%	52%	60%	67%	74%	80%	85%	89%
Increase in Sample Size to Restore 90% Conditional Power	No increase	240	180	140	110	70	40	10

[1] Conditional Power based on the alternative hypothesis effect size as observed in the first 60 subjects.

For Table 6, the conditional power estimates and the numbers to increase the sample size to restore 90% conditional power were calculated in PASS version 14 using the module for two-sample t-tests, which is based on the conditional power definitions from Jennison and Turnbull [7]. Since the minimum effect size for all possible scenarios where the sample size would be increased achieves greater than 50% conditional power for N=60, the type-I error rate is preserved with this administrative sample size re-estimation method [8]. However, the sponsor has chosen to allocate 2% of the alpha (0.0001) for this unblinded administrative interim analysis to account for any possible downward bias in the variance estimate. At the time of the interim analysis, the independent unblinded statistician will fit the cleaned data on the first 60 participants to the primary analysis model to estimate the parameters. These parameter estimates will be input to the PASS 14 Mixed Model Repeated Measures module to re-estimate the power. If the power is less than 90% and more than 50%, the independent statistician will increase the sample size up to the point where the power reaches 90%. These results will be provided by the unblinded independent statistician to the DMC.

The DMC will review the group-unblinded interim analysis results provided by the independent statistician and will give recommendations to the designated sponsor representative from MAPS. The designated sponsor representative from MAPS will provide written information to the trial organizer delegate of MPBC indicating how many participants, if any, to add to the IWRS but will not provide any information on the corresponding conditional power the additional participants are being added to achieve. Only the re-estimation of sample size will be completed at the interim analysis, this will not be used to drop or add treatment arms or doses, change entry criteria, change randomization ratio or change primary endpoint. The sponsor representative from MAPS will not be part of any trial decisions or administration after the sample size re-estimation has been communicated to MPBC who will continue to be the trial organizer. If the DMC reports a sample size increase to the independent statistician DMC member, they will increase the randomization cap from 100 to the new sample size within the IWRS accordingly. Thereafter, the IWRS will cap randomization at the increased sample size resulting from the interim analysis. If the interim analysis results in a sample size increase, it will not be revealed to the investigators, participants, or sponsor.

9.0 Data Monitoring Committee

A DMC with appropriate expertise in the conduct of PTSD clinical trials will act in an advisory capacity to monitor participant safety on a routine basis throughout the trial by reviewing safety and study data provided by an Independent Statistical Group. The DMC will also monitor individual participant tolerability. The composition of the DMC will include two clinician experts in PTSD clinical trials and a biostatistician.

The DMC may:

- Review the study protocol and informed consent documents, and plans for data monitoring
- Evaluate the progress of the trial, study data quality, timeliness, participant recruitment, accrual and retention, participants’ risk versus potential benefit, and other factors that could affect the study outcome
- Implement the adaptive design aspects of the study at the interim analysis by reviewing the interim analysis results and making the appropriate decision to the independent statistician on sample size re-estimation
- Consider relevant information that may have an impact on the safety of the participants or the ethics of the study
- Make other recommendations to MAPS concerning continuation, termination or other modifications of the study based on their observations of the study.

A full description of the DMC duties will be detailed in the DMC Charter.

10.0 Timing of Analyses

The primary efficacy analysis will be conducted after all participants complete Visit 20 and the database is locked.

References

1. Carrasco, J.L., et al., *Estimation of the concordance correlation coefficient for repeated measures using SAS and R.* Comput Methods Programs Biomed, 2013. **109**(3): p. 293-304.
2. Tang, Y., *An efficient monotone data augmentation algorithm for multiple imputation in a class of pattern mixture models.* J Biopharm Stat, 2017. **27**(4): p. 620-638.
3. Molenberghs, G., et al., *Analyzing incomplete longitudinal clinical trial data.* Biostatistics, 2004. **5**(3): p. 445-64.
4. Rubin, D.B., *Inference and missing data.* Biometrika, 1976. **63**(3): p. 581-592.
5. Sheehan, K.H. and D.V. Sheehan, *Assessing treatment effects in clinical trials with the discan metric of the Sheehan Disability Scale.* Int Clin Psychopharmacol, 2008. **23**(2): p. 70-83.
6. Nilsson, M.E., et al., *Columbia Suicide Severity Rating Scale Scoring and Data Analysis Guide*, in *CSSRS Scoring Version 2.0*. 2013: http://www.cssrs.columbia.edu/documents/ScoringandDataAnalysisGuide_Feb2013.pdf. p. 1-13.
7. Jennison, C. and B.W. Turnbull, *Group sequential methods with applications to clinical trials*. 2000, Boca Raton, FL: Chapman & Hall/CRC Press.
8. Chen, Y.H., D.L. DeMets, and K.K. Lan, *Increasing the sample size when the unblinded interim result is promising.* Stat Med, 2004. **23**(7): p. 1023-38.
